# Evolution of selenophosphate synthetases: emergence and relocation of function through independent duplications and recurrent subfunctionalization

**DOI:** 10.1101/gr.190538.115

**Published:** 2015-09

**Authors:** Marco Mariotti, Didac Santesmasses, Salvador Capella-Gutierrez, Andrea Mateo, Carme Arnan, Rory Johnson, Salvatore D'Aniello, Sun Hee Yim, Vadim N. Gladyshev, Florenci Serras, Montserrat Corominas, Toni Gabaldón, Roderic Guigó

**Affiliations:** 1 Bioinformatics and Genomics Programme, Centre for Genomic Regulation (CRG), 08003 Barcelona, Catalonia, Spain;; 2 Universitat Pompeu Fabra (UPF), 08003 Barcelona, Catalonia, Spain;; 3 Institut Hospital del Mar d'Investigacions Mèdiques (IMIM), 08003 Barcelona, Catalonia, Spain;; 4 Division of Genetics, Department of Medicine, Brigham and Women's Hospital, Harvard Medical School, Boston, Massachusetts 02115, USA;; 5 Departament de Genètica, Facultat de Biologia and Institut de Biomedicina (IBUB) de la Universitat de Barcelona (UB), 08028 Barcelona, Catalonia, Spain;; 6 Department of Biology and Evolution of Marine Organisms, Stazione Zoologica Anton Dorhn, Villa Comunale, 80121, Napoli, Italy;; 7 Institució Catalana de Recerca i Estudis Avançats (ICREA), 08010 Barcelona, Catalonia, Spain

## Abstract

Selenoproteins are proteins that incorporate selenocysteine (Sec), a nonstandard amino acid encoded by UGA, normally a stop codon. Sec synthesis requires the enzyme Selenophosphate synthetase (SPS or SelD), conserved in all prokaryotic and eukaryotic genomes encoding selenoproteins. Here, we study the evolutionary history of *SPS* genes, providing a map of selenoprotein function spanning the whole tree of life. SPS is itself a selenoprotein in many species, although functionally equivalent homologs that replace the Sec site with cysteine (Cys) are common. Many metazoans, however, possess *SPS* genes with substitutions other than Sec or Cys (collectively referred to as *SPS1*). Using complementation assays in fly mutants, we show that these genes share a common function, which appears to be distinct from the synthesis of selenophosphate carried out by the Sec- and Cys- *SPS* genes (termed *SPS2*), and unrelated to Sec synthesis. We show here that *SPS1* genes originated through a number of independent gene duplications from an ancestral metazoan selenoprotein *SPS2* gene that most likely already carried the *SPS1* function. Thus, in *SPS* genes, parallel duplications and subsequent convergent subfunctionalization have resulted in the segregation to different loci of functions initially carried by a single gene. This evolutionary history constitutes a remarkable example of emergence and evolution of gene function, which we have been able to trace thanks to the singular features of *SPS* genes, wherein the amino acid at a single site determines unequivocally protein function and is intertwined to the evolutionary fate of the entire selenoproteome.

Selenoproteins are proteins that incorporate the nonstandard amino acid selenocysteine (Sec or U) in response to the UGA codon. The recoding of UGA, normally a stop codon, to code for Sec is arguably the most outstanding programmed exception to the genetic code. Selenoproteins are found, albeit in small numbers, in organisms across the entire tree of life. Recoding of UGA is mediated by RNA structures within selenoprotein transcripts, the SECIS (SElenoCysteine Insertion Sequence) elements. Sec biosynthesis and insertion also require a dedicated system of *trans*-acting factors that include elements that are common and others that are specific to the three domains of life: bacteria (Kryukov and Gladyshev 2004; Yoshizawa and Böck 2009), archaea (Rother et al. 2001), and eukaryotes (Squires and Berry 2008; Allmang et al. 2009).

The very existence of selenoproteins is puzzling. Sec can apparently be substituted by cysteine (Cys)—as often happens during evolution (Zhang et al. 2006; Chapple and Guigó 2008; Mariotti et al. 2012)—with seemingly a small or null impact on protein function. In fact, selenoproteins may be absent in an entire taxonomic group but present in sister lineages. This can be seen most dramatically within fruit flies: Although *Drosophila melanogaster* and most other flies possess three selenoprotein genes, their relative *Drosophila willistoni* has replaced Sec with Cys in them and lost the capacity to synthesize Sec (Chapple and Guigó 2008; Lobanov et al. 2008). Fungi and plants have also lost this capacity (Lobanov et al. 2009). In other cases, however, such as in *Caenorhabditis elegans*, the entire pathway is maintained only to synthesize a single selenoprotein (Taskov et al. 2005). It appears that selective pressure exists to maintain Sec, at least in vertebrates, since strong purifying selection across Sec sites that prevent mutations to Cys has been reported (Castellano et al. 2009). Sec encoding has been hypothesized to be an ancestral trait, already present in the early genetic code, since a number of selenoprotein families are shared between prokaryotes and eukaryotes. However, the evolutionary continuity of the Sec recoding systems across domains of life is not certain, since it would require the translocation of the SECIS element (within the coding region in bacteria but within the 3′ UTR in eukaryotes) as well as the radical alteration of its structure.

Selenophosphate synthetase (*SPS*, also called *SelD* or selenide water dikinase) is unique among the components of the Sec biosynthesis machinery in that it is often a selenoprotein itself. SPS catalyzes the synthesis of selenophosphate from selenide, ATP, and water, producing AMP and inorganic phosphate as products. Selenophosphate is the selenium donor for the synthesis of Sec, which, in contrast to other amino acids, takes place on its own tRNA, *tRNAsec* (Xu et al. 2007a; Palioura et al. 2009).

SPS proteins are conserved from bacteria to human with ∼30% identity and are found in all species known to encode selenoproteins. In prokaryotes, *SPS* (i.e., *SelD*) is found also in species where selenophosphate is used to produce selenouridine in tRNAs (SeU). In these species, it acts as the selenium donor to protein ybbB (selenouridine synthase). The presence of the two traits (SeU and Sec) overlaps, but not completely (Romero et al. 2005). In eukaryotes, SPS is generally found as a selenoprotein, whereas in prokaryotes, homologs with Cys aligned to the Sec position are common. As for all selenoproteins, Sec and Cys homologs of SPS are expected to perform the same molecular function, although catalytic efficiency can vary. Indeed, selenophosphate synthesis activity has been demonstrated experimentally for various Sec- and Cys- SPS proteins, as well as for artificial Cys mutants (Kim et al. 1997; Persson et al. 1997; Xu et al. 2007a).

In vertebrates and insects, two paralogous *SPS* genes have been reported: *SPS2* (i.e., *Sephs2*), which is a selenoprotein, and *SPS1* (i.e., *Sephs1*), which is not and carries a threonine (Thr) in vertebrates and an arginine (Arg) in insects in place of Sec (Xu et al. 2007b). In contrast to Cys conversion, Thr or Arg conversion in SPS1 seems to result in the abolishment of the selenophosphate synthase function. Indeed, murine SPS1 does not generate selenophosphate in vitro and does not even consume ATP in a selenium dependent manner (Xu et al. 2007a). Consistently, selenoprotein synthesis is unaffected in a knockout of *SPS1* in mouse cell lines (Xu et al. 2007b). Similarly, *Drosophila SPS1* (i.e., *ptuf/SelD*) lacks the ability to catalyze selenide-dependent ATP hydrolysis or to complement *SelD* deficiency in *Escherichia coli* (Persson et al. 1997). In insects, *SPS1* is preserved in species that lost selenoproteins (Chapple and Guigó 2008). Although human SPS1 (i.e., SEPHS1) interacts with Sec synthase (SecS) (Small-Howard et al. 2006), these findings taken as a whole suggest that *SPS1* functions in a pathway unrelated to selenoprotein biosynthesis (Lobanov et al. 2008). What the function of *SPS1* may be remains an open question. Human *SPS1* (i.e., *SEPHS1*) has been proposed to function in Sec recycling, since an *E. coli SelD* mutant can be rescued by *SPS1* but only when grown in the presence of L-selenocysteine (Tamura et al. 2004). In *Drosophila*, *SPS1* has been proposed to be involved in vitamin B6 metabolism (Lee et al. 2011) and in redox homeostasis since it protects from damage induced by reactive oxygen species (ROS) (Morey et al. 2003).

Here, we study the evolutionary history of *SPS* genes across the tree of life. We found that the presence of Sec/Cys *SPS* genes, together with a few other gene markers, recapitulates the selenium utilization traits (Sec and SeU) in prokaryotic genomes. Within eukaryotes, specifically within metazoans, we detected a number of *SPS* homologs with amino acids other than Sec or Cys at the homologous UGA position. We found that Cys- or Sec-containing *SPS* genes (*SPS2*) are found in all genomes encoding selenoproteins, whereas genomes that contain only *SPS* genes carrying amino acids other than Sec or Cys at the homologous UGA position (*SPS1*) do not encode selenoproteins. In SPS proteins, thus, it appears that the residue occurring at a single site is a precise marker of function, which can be easily traced in genomes by searching for selenoprotein genes and other markers of selenium utilization. This feature, that may be unique among all protein families, makes *SPS* genes singularly appropriate to investigate the evolution of gene function. Here, thanks to this feature, we have been able to untangle the complex history of *SPS* genes with great detail. Our analysis reveals that *SPS1* genes in different metazoan lineages (including those of human and fly) originated via parallel duplications from an ancestral Sec-carrying *SPS2* gene. Despite their independent origin, *SPS1* genes share similar evolutionary constraints and have a common function, likely present in the ancestral metazoan *SPS2* gene. This indicates selective pressure during metazoan history to segregate different functions to separate loci and constitutes a remarkable example of recurrent escape from adaptive conflict through gene duplication and subfunctionalization (Hittinger and Carroll 2007). Within insects, the *SPS* duplication was followed by the loss of the Sec-encoding *SPS2* gene in several lineages, which therefore lost the capacity to synthesize selenoproteins (Chapple and Guigó 2008; Lobanov et al. 2008)—becoming, together with some nematodes (Otero et al. 2014), the only known selenoproteinless metazoans. Strikingly, *SPS1* conserved the ancestral UGA codon in selenoproteinless *Hymenoptera*. Our analyses point out that UGA readthrough in hymenopterans is enhanced by overlapping RNA structures, also present in other selenoproteins. These structures could be related to bacterial SECIS elements, uncovering a possible evolutionary link between the prokaryotic and eukaryotic Sec-encoding systems. They would have played a key role throughout the evolution of *SPS* genes, particularly in the emergence of the *SPS1* function in the metazoan ancestral Sec-carrying *SPS* gene.

## Results

Selenoprotein genes are usually misannotated in prokaryotic and eukaryotic genomes due to the recoding of UGA, normally a stop codon, to Sec. Therefore, we used Selenoprofiles (Mariotti and Guigó 2010), a computational tool dedicated to the prediction of selenoproteins and selenoprotein homologs. We have run Selenoprofiles to search for *SelD/SPS* genes in all available fully sequenced eukaryotic and prokaryotic genomes, 505 and 8263, respectively. We then utilized a combination of approaches to reconstruct their phylogenetic history. Methods and analyses are fully discussed in Supplemental Material S1–S6.

### SelD as a marker for selenium utilization in prokaryotes

**Figure 1.**
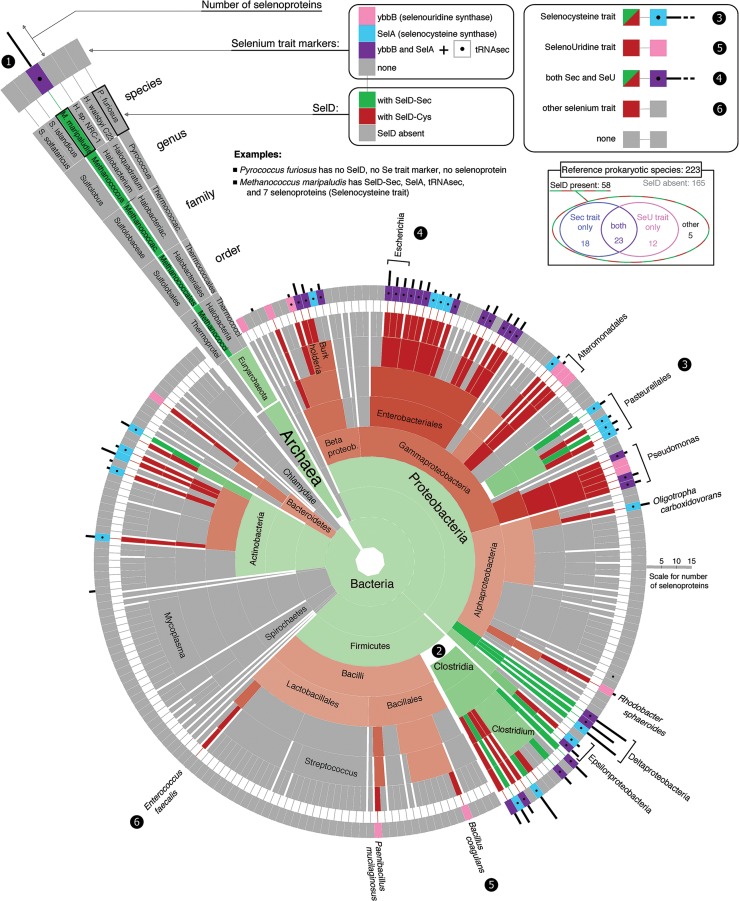
(Enclosed poster) Phylogenetic profile of *SPS* and selenium utilization traits in prokaryotes. The sunburst tree shows the phylogenetic structure of the reference set of 223 prokaryotic genomes (taken from NCBI taxonomy) and the presence of *SelD* genes and other markers of selenium utilization.

Figure 1 (see the enclosed poster that accompanies this issue) shows the distribution of *SelD* (i.e., prokaryotic *SPS*) genes in a reference set of 223 prokaryotic genomes (Pruitt et al. 2012), along with the presence of other selenium utilization gene markers such as the bacterial selenocysteine synthase gene (*SelA*). The occurrence of *SelD* and other Sec machinery components is in good agreement with previous findings (Zhang et al. 2006, 2008; Zhang and Gladyshev 2008, 2010). Supplemental Material S1 contains details of the genes found in each major lineage investigated, both in the reference set and in the extended set of 8263 genomes.

*SelD* genes were found in 26% of the reference prokaryotic genomes. A considerable fraction (19%) of the detected *SelD* genes encoded a protein with a Sec residue (always in the same position), with all the rest containing Cys instead. The occurrence of *SelD* is, in general, consistent with the other gene markers for selenium utilization and also with selenoprotein presence (Fig. 1, poster), with only a few exceptions (Supplemental Material S1). The Sec trait (*SelD*, *SelA*, *tRNAsec*, selenoproteins) was found in a slightly larger group of organisms than the SeU trait (*SelD*, *ybbB*): 18% versus 16%, respectively. The two traits showed a highly significant overlap: 10% of all species had both (*P*-value < 0.0001, one-tailed Fisher's exact test). The Sec and SeU markers showed a scattered distribution across the prokaryotic tree, reflecting the dynamic evolution of selenium utilization. The complexity of this phylogenetic pattern is even more evident when considering the extended set of prokaryotic species (Supplemental Material S1; Supplemental Fig. SM1.1).

In almost every species with *SelD* (93%), genes for *SelA* and/or *ybbB* were identified, supporting the utilization of selenophosphate for Sec and SeU. A notable exception was the *Enterococcus* genus, where many species, including *Enterococcus faecalis*, possessed *SelD* but no other markers of selenium usage. This had already been reported as an indicator of a potential third selenium utilization trait (Romero et al. 2005; Zhang et al. 2008). Selenium is in fact used by these species as a cofactor to molybdenum hydroxylases (Haft and Self 2008; Srivastava et al. 2011).

In *Pasteurellales*, an order within *Gammaproteobacteria*, we identified a bona fide Cys-to-Sec conversion. Most of *Gammaproteobacteria* possess a *SelD-Cys* (or none), and Sec forms are found almost uniquely in *Pasteurellales*. Phylogenetic sequence signal supports codon conversion rather than horizontal transfer as the cause for *SelD-Sec* (Supplemental Material S1). Although cases of conversion of Cys to Sec have been proposed (Zhang et al. 2006), this is the first clearly documented case. Among archaea, *SelD* was found only in *Methanococcales* and *Methanopyri* genomes, whose selenoproteins have been previously characterized (Stock and Rother 2009). The SeU trait was found only in *Methanococcales*, although with a peculiarity: *ybbB* is split in two adjacent genes (Su et al. 2012).

Previous reports have described a number of *SPS* genes fused to other genes (Zhang et al. 2008; da Silva et al. 2013). Thus, we used a computational strategy to identify *SelD* gene fusions or extensions (Methods; Supplemental Material S2). Fusions with a NADH dehydrogenase–like domain (Zhang et al. 2008) are by far the most common, and they are found scattered across a wide range of bacteria (Supplemental Fig. SM2.1). We also detected two instances of fusions with the NifS-like protein—Cys sulfinate desulfinase, proteins that deliver selenium for the synthesis of selenophosphate by SPS2 (Lacourciere et al. 2000). In all cases, the extension/fusion is on the N-terminal side of the *SPS* genes, and these are always *SelD-Cys* with the single exception of *NifS-SPS* in *Geobacter sp. FRC-32*, which contains Sec. Since we found selenoproteins and other Sec machinery genes in all these genomes, we predict that generally these extended *SPS* genes have retained the original selenophosphate biosynthetic activity.

### SPS2 as a marker for Sec utilization in eukaryotes

**Figure 2.**
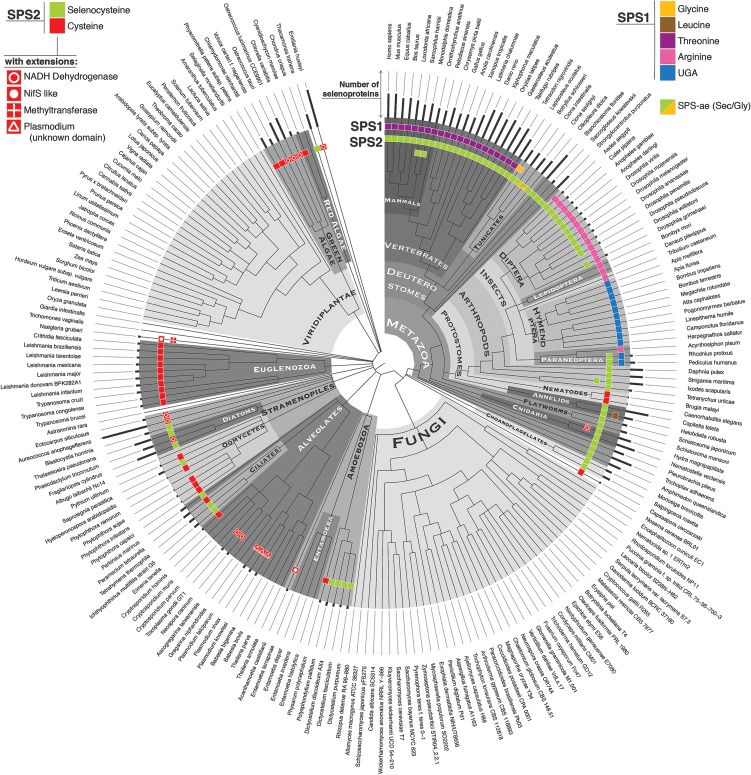
(Enclosed poster) Phylogenetic profile of *SPS* genes and approximate selenoproteome size of eukaryotes. The plot recapitulates the results on 505 genomes analyzed, summarized to 213 displayed here.

Figure 2 (see the enclosed poster that accompanies this issue) shows *SPS* genes and predicted selenoproteins found in a representative set of eukaryotic genomes. The presence of *SPS2* genes (defined as those with Sec or Cys) correlates perfectly with the presence of selenoproteins. Thus, *SPS2* is a marker of Sec utilization in eukaryotes. Our results replicate and substantially expand previous surveys of selenoproteins in eukaryotic genomes (e.g., Lobanov et al. 2007, 2009; Chapple and Guigó 2008; Jiang et al. 2012).

Overall, the Sec trait exhibits a rather scattered distribution in protists reflecting a dynamic evolution similar to bacteria. We found *SPS2* (and therefore selenoproteins) scattered across *Stramenopiles*, *Alveolata*, *Amoebozoa*, and other protist lineages, presumably due to multiple independent events of selenoprotein extinction. In contrast, we found selenoproteins in all investigated *Kinetoplastida* (*Euglenozoa*), including the parasites *Trypanosoma* and *Leishmania* (Cassago et al. 2006; Lobanov et al. 2006). We did not find any bona fide *SPS2* nor selenoproteins in fungi or land plants (*Embryophyta*), despite that many genomes were searched (284 and 41, respectively, a subset of which are shown in Fig. 2, poster). In contrast, green algae genomes contain large numbers of selenoproteins as previously reported (Novoselov et al. 2002; Palenik et al. 2007). The largest number was in the pelagophyte algae *Aureococcus anophagefferens* (heterokont, Stramenopiles), known for its rich selenoproteome (Gobler et al. 2013). All metazoans encode *SPS2* and selenoproteins, with exceptions detected so far only in some insects (Chapple and Guigó 2008; Lobanov et al. 2008) and some nematodes (Otero et al. 2014).

As in prokaryotes, we found a few protist genomes in which *SPS2* is fused to other genes. Fusions with a NADH dehydrogenase–like domain are also the most common and scattered among many taxa (Fig. 2, poster). We detected *NifS-SPS* fusions in the amoeba, *Acanthamoeba castellani*, and the heterolobosean, *Naegleria gruberi*. In these two genomes, we found additional *SPS2* candidates (Supplemental Material S2). In *N. gruberi*, SPS2 is fused to a polypeptide containing a methyltransferase domain (da Silva et al. 2013). Finally, all SPS2 proteins in *Plasmodium* species were found to possess a large polypeptide extension (>500 amino acids). This domain shows no homology with any known protein, and its function remains unknown. We did not find convincing *SPS* fusions in nonprotist eukaryotes (see Supplemental Material S2).

The reconstructed gene tree of the bacterial, archaeal, and eukaryotic *SPS* sequences follows broadly their known phylogenetic relationships (Supplemental Fig. SM3.1) and supports the continuity of the selenoprotein system across the three domains of life. Most likely, thus, the last common ancestor of eukaryotes and prokaryotes possessed selenoproteins and *SPS*, probably as a selenoprotein itself. The continuity in *SPS* phylogenetic signal is apparently broken only in a few protist lineages, which seem to have acquired a bacterial-like *SPS* gene by horizontal transfer (Supplemental Material S3). All the gene fusions shared by protists and bacteria (*NADH-SPS*, *NifS-SPS*) are explained by this process (i.e., they are not independently evolved fusions but the result of a fusion event in bacteria, which was subsequently transmitted horizontally).

### Independent duplications of *SPS2* generates SPS1 proteins in metazoans

**Figure 3.**
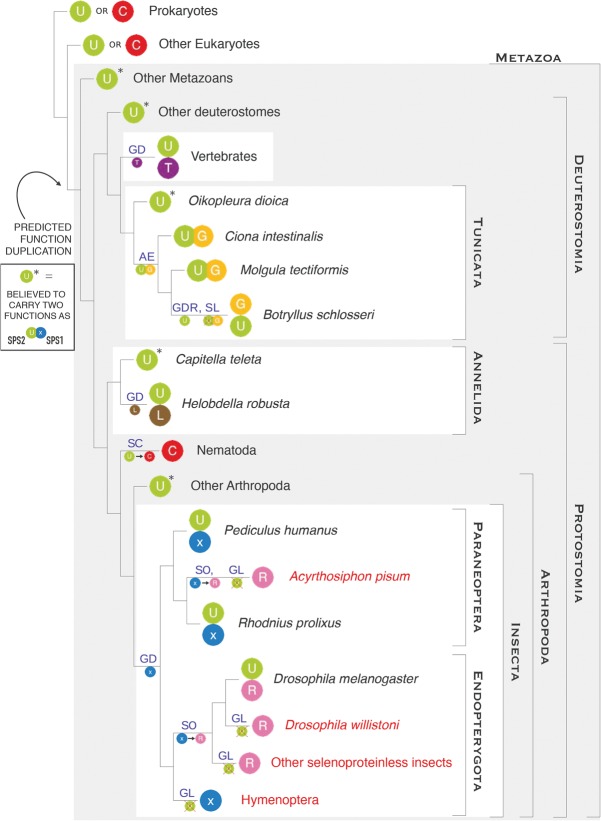
Parallel gene duplications of SPS proteins in metazoa. The plot summarizes the phylogenetic history of metazoan *SPS* genes, consisting of parallel and convergent events of gene duplication followed by subfunctionalization. Each colored ball represents a *SPS* gene, indicating the residue found at the UGA or homologous codon: (U) selenocysteine; (C) cysteine; (T) threonine; (G) glycine; (L) leucine; (R) arginine; (x) unknown residue. The gene structures are schematically displayed in Figure 4. The names of the insect species lacking selenoproteins are in red. The main genomic events shaping *SPS* genes are indicated on the branches: (GD) whole gene duplication; (GDR) gene duplication by retrotransposition; (AE) origin of an alternative exon; (SL) Sec loss; (SC) conversion of Sec to Cys; (SO) conversion of Sec to something other than Cys; (GL) gene loss. In our subfunctionalization hypothesis (see text), we map the origin of a dual function at the root of metazoa. A star (*) marks the metazoan *SPS2-Sec* genes which did not duplicate. These genes are expected to possess dual function.

**Figure 4.**
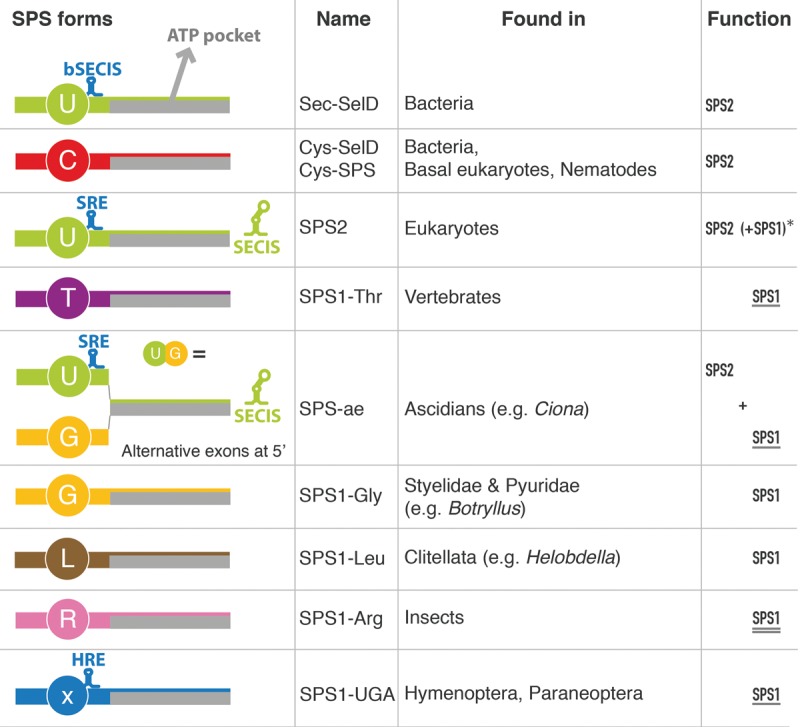
Structure and function of the identified *SPS* genes. SPS proteins are classified according to the residue found at the UGA or homologous position (Fig. 3). The presence of specific secondary structures is also indicated: (bSECIS) bacterial SECIS element; (SRE) Sec recoding element (Howard et al. 2005); (SECIS) eukaryotic SECIS element; (HRE) hymenopteran readthrough element. The *rightmost* column indicates the functions predicted for the SPS proteins. *SPS2* function is the synthesis of selenophosphate. *SPS1* function is defined as the uncharacterized molecular function of *Drosophila SPS1-Arg* (double underlined), which is likely to be similar to that of other *SPS1* genes, as suggested by knockout-rescue experiments in *Drosophila* (underlined). (*) Eukaryotic *SPS2*; the parentheses indicate that some such genes are predicted to possess both *SPS1* and *SPS2* functions, those marked also with a star (*) in Figure 3 (essentially all metazoans with no SPS1 protein in the same genome).

In many metazoan lineages we detected additional *SPS* genes, which are neither selenoproteins nor Cys-homologs. Because within metazoans, *SPS-Cys* are found only in nematodes, and outside metazoans, additional *SPS* genes are absent (Fig. 2, poster), we argue that the last common metazoan ancestor possessed a single *SPS* gene with Sec (i.e., it was *SPS2*). Our results show that the additional *SPS* genes do not have a single origin, but instead they were generated by independent duplications of *SPS2* in a number of metazoan lineages (Supplemental Material S3). We have specifically identified four independent duplications (Figs. 3, 4). One *SPS* duplication occurred at the root of the vertebrates, probably as part of one of the reported rounds of whole genome duplication (Dehal and Boore 2005). Another duplication occurred within tunicates, likely originated by retrotransposition of an alternative isoform of *SPS2*. Another duplication occurred within annelids, at the root of the *Clitellata* lineage. Finally, a duplication occurred at the root of insects. In each of these duplications, a specific substitution of the Sec residue was fixed: threonine in vertebrates, glycine in tunicates, and leucine in annelids. In insects, however, the UGA codon was maintained after duplication and substituted in some lineages by arginine. There were at least two independent UGA to arginine substitutions in *Paraneoptera* and *Endopterygota*. Remarkably, insects without selenoproteins lost the original *SPS2* gene but maintained the duplicated copy.

Therefore, as a universal trend, Cys- or Sec-containing *SPS* genes (to which we refer as *SPS2*) are found in all (prokaryotic and eukaryotic) genomes encoding selenoproteins, whereas the metazoan genomes containing only *SPS* genes carrying amino acids other than Sec or Cys at the homologous UGA position do not encode selenoproteins. Thus, the duplicated non-Cys, non-Sec copies of *SPS2* in human and fly are unlikely to carry a function related to selenoprotein synthesis. Since in human and fly, they are commonly referred to as *SPS1* (Xu et al. 2007b), we will collectively refer to all non-Cys, non-Sec *SPS2* duplications in metazoans as *SPS1* (e.g., *SPS1-Thr* for human *SPS1*).

In the next section, we briefly describe each SPS duplication separately.

### *SPS* phylogeny in vertebrates

All nonvertebrate deuterostomes (except tunicates) and *Cyclostomata* (jawless vertebrates, such as lampreys) encode only one *SPS2* gene, and all *Gnasthostomata* possess *SPS1* in addition. Supported also by a strong phylogenetic signal (Supplemental Fig. SM3.2), we conclude that vertebrate *SPS1* (*SPS1-Thr*) originated from a duplication of *SPS2* concomitant with conversion of Sec to threonine at the root of *Gnasthostomata* (Supplemental Material S3). The conservation of intron positions within the protein sequence is consistent with the duplication involving the whole gene structure; and given its phylogenetic position, this may have occurred through one of the reported rounds of whole genome duplication at the base of vertebrates (Dehal and Boore 2005). As recently reported (Mariotti et al. 2012), in mammals the *SPS2* gene duplicated again, this time by retrotransposition, generating a second *SPS2-Sec* gene almost identical to the parental, except for the lack of introns. In placental mammals, the intronless *SPS2* functionally replaced the parental gene (which was lost), while nonplacental mammals still retain the two copies (e.g., *Monodelphis domestica*) (Fig. 2, poster).

### *SPS* phylogeny in tunicates

**Figure 5.**
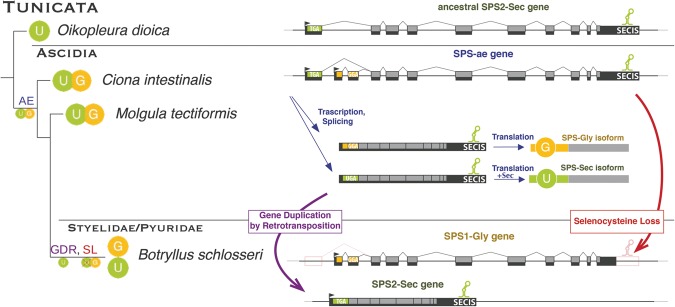
Alternative *SPS1/2* transcript isoforms sorted by retrotransposition within ascidians. This figure expands the section for tunicates in Figure 3. At the root of ascidians, the ancestral *SPS2-Sec* gene acquired a novel *SPS-Gly* transcript isoform through alternative exon usage at the 5′ end (AE). Then, at the root of the ascidian lineage, *Styelidae* and *Pyuridae*, the *SPS-Sec* transcript of this dual *SPS1/SPS2* gene (*SPS-ae*) retrotransposed to the genome creating a novel *SPS2-Sec* gene (GDR). This presumably triggered the loss of Sec from the parental gene, which, because both the SECIS and the UGA containing exon degenerated (SL), specialized only in the production of *SPS1-Gly*.

Tunicates are the closest outgroup to vertebrates (Delsuc et al. 2006), with ascidians (sea squirts) constituting the best-studied and most sequenced lineage. In the ascidian *Ciona*, we identified a single *SPS* gene. This appears to be the direct descendant of the ancestral metazoan *SPS2* and possesses a SECIS element in the 3′ UTR (necessary for the incorporation of Sec). Nonetheless, this gene produces two different protein isoforms, deriving from alternative exon structures at the 5′ end (*SPS-ae*) (Supplemental Material S4). One isoform carries Sec (*SPS-Sec*, corresponding to the *SPS2*), whereas the other one, previously unreported, has a glycine instead (*SPS-Gly*, corresponding to *SPS1*). We mapped the origin of the *SPS-Gly* isoform to the root of ascidians since the non-ascidian tunicate *Oikopleura dioica* appears to have only the *SPS2-Sec* gene with a single isoform, whereas both isoforms (*SPS-Sec* and *SPS-Gly*) are found in the ascidian *Molgula tectiformis* (Fig. 5). We also found both forms in the recently sequenced ascidian species *Botryllus schlosseri* (Voskoboynik et al. 2013) and *Halocynthia roretzi*, belonging to the sister lineages of *Styelidae* and *Pyuridae*, respectively. However, in these species, the two forms mapped to distinct genomic loci, and they correspond therefore to two different genes (Supplemental Material S4). *SPS-Sec* is intronless and contains a SECIS within the 3′ UTR. It corresponds, thus, to *SPS2*. *SPS-Gly* possesses instead the ancestral intron structure (very similar to *O. dioica SPS2*) and has no SECIS. It corresponds, therefore, to *SPS1*. Most likely, the ancestral *SPS-sec* alternative transcript isoform retrotransposed to the genome at the root of *Styelidae* and *Pyuridae*. This generated a copy that soon functionally replaced the *SPS-Sec* isoform of the parental gene, which as a result specialized in the production only of the *SPS-Gly* isoform, as both the Sec coding exon and the SECIS element degenerated. This exemplifies an evolutionary scenario, not frequently reported in the literature, in which alternative transcripts precede gene duplication, providing a possible intermediary step of how a dual-function protein can escape from adaptive conflict (Hittinger and Carroll 2007).

### *SPS* phylogeny in insects

Insects provide a unique framework to study selenoprotein evolution. They have undergone several waves of complete selenoprotein extinction, in which selenoprotein genes were converted to Cys homologs or lost, and the Sec machinery degenerated and/or disappeared. This process occurred in several lineages independently: *Hymenoptera, Lepidoptera, Coleoptera* (or at least *Tribolium castaneum*), in the *Drosophila willistoni* lineage (Chapple and Guigó 2008; Lobanov et al. 2008) within *Endopterygota*, and also in the paraneopteran pea aphid (*Acyrthosiphon pisum*) (The International Aphid Genomics Consortium 2010). Consistent with its function, the *SPS-Sec* (*SPS2*) gene is present in every insect genome coding for selenoproteins, and it is absent in every insect genome not coding for selenoproteins (Fig. 2, poster). *SPS1* genes, in contrast, are present in all insect genomes. Most insect *SPS1* genes (e.g., in *Lepidoptera*, *Coleoptera*, *Diptera*) use arginine at the Sec/Cys site (*SPS1-Arg*) (Figs. 3, 4). Instead, *SPS1* has a UGA codon at this position in *Hymenoptera* and in two nonmonophyletic species within paraneopterans: *Rhodnius prolixus* and *Pediculus humanus*. In these, however, and in contrast to hymenopterans, we found an additional *SPS-Sec* gene with SECIS element (*SPS2*) and consistently, a number of other selenoproteins and the complete Sec machinery. From all these data (Supplemental Material S3), we hypothesize (Fig. 3) that all insect *SPS1* genes derive from the same *SPS2* duplication event that occurred approximately at the root of insects, initially generating a UGA-containing, SECIS-lacking gene (*SPS1-UGA*). In most lineages, the new gene switched the UGA codon to arginine, generating *SPS1-Arg* proteins. This occurred at least twice independently, in the pea aphid and in the last common ancestor of *Coleoptera*, *Lepidoptera*, and *Diptera*. In *Hymenoptera* and most *Paraneoptera*, the gene is still conserved with UGA and no SECIS. The original *SPS2* was lost in all lineages where selenoproteins disappeared.

### SPS1-UGA: non-Sec readthrough

The strong conservation of the UGA codon in hymenopteran/paraneopteran *SPS1-UGA* aligned exactly at the position of the *SPS2* Sec-UGA codon is extremely puzzling. *SPS1-UGA* does not contain a SECIS element. Furthermore, *Hymenoptera* lack most constituents of the Sec machinery, and these organisms cannot synthesize selenoproteins. However, the striking conservation of the insect *SPS1-UGA* sequence strongly indicates that it is translated and functional. Previously, we had hypothesized that *SPS1-UGA* could perhaps be translated by a readthrough mechanism not involving Sec insertion (Chapple and Guigó 2008). In this respect, there is growing evidence for abundant stop codon readthrough in insects, with UGA being the most frequently observed readthrough codon in *Drosophila* (Jungreis et al. 2011).

Here, we have found additional strong evidence in support of the translational recoding of *SPS1-UGA*. First, all 10 recently sequenced hymenopteran genomes show a clear pattern of protein coding conservation across the UGA, resulting in a readthrough protein of an approximate size of SPS.

**Figure 6.**
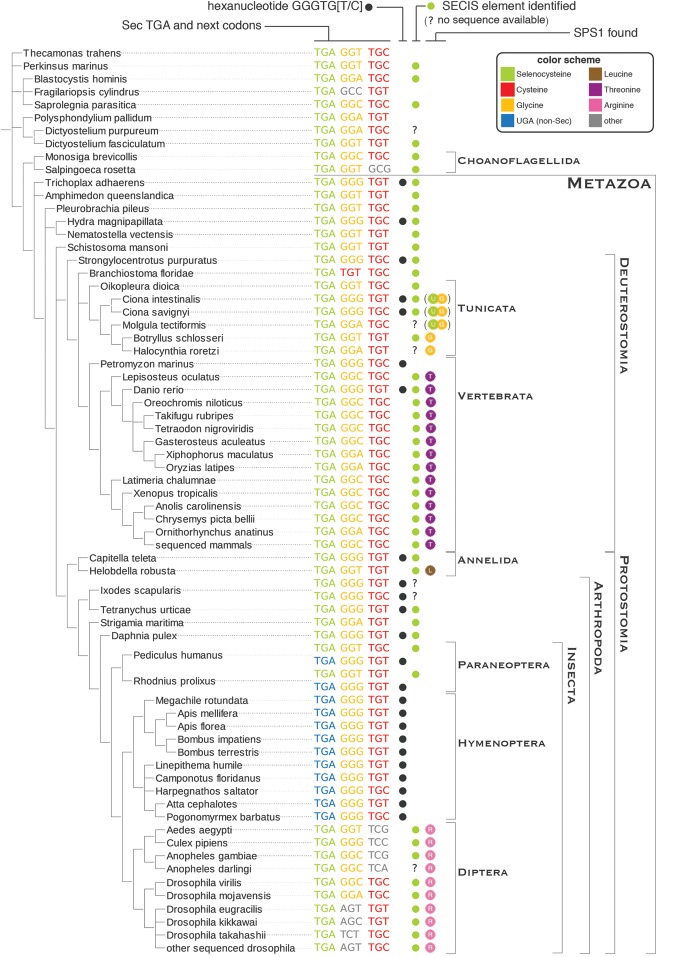
Readthrough-enhancing hexanucleotide in *SPS* genes. The phylogenetic tree on the *left* shows the nucleotide sequence alignment at the UGA (or homologous) site in *SPS* sequences. Only *SPS2* and *SPS1-UGA* genes are shown here. Codons are colored according to their translation, following the same color schema used in Figures 2 and 4 (gray for other amino acids). The presence of the hexanucleotide described in Harrell et al. (2002) is marked with a black dot. Green dots mark the genes for which a bona fide SECIS element was identified. The *last* column indicates the presence of *SPS1* genes.

Second, we found the hexanucleotide GGG-UG[C/U], which is highly overrepresented next to known viral “leaky” UAG stop codons (Harrell et al. 2002) to be ultraconserved subsequent to the UGA in *SPS1-UGA* genes. Although the hexanucleotide is found scattered in some other metazoan *SPS2* sequences—where it could actually contribute to UGA translation—it is absent from all insect *SPS2* genes (Fig. 6). Moreover, the hexanucleotide is also absent from insect *SPS1* genes having Arg instead of UGA.

Third, *SPS1-UGA* contains a conserved secondary structure overlapping the UGA (and therefore the hexanucleotide). We will name this structure HRE, for hymenopteran readthrough element. For its location and structure (Supplemental Material S6; Supplemental Fig. SM6.2), HRE appears to derive from stem–loop structures called SRE (from Sec redefinition elements), previously identified in many selenoprotein genes including *SPS2* (Howard et al. 2005) and that promote readthrough activity. Indeed, the SRE element of human selenoprotein N gene (*SEPN1*) was functionally characterized (Howard et al. 2005, 2007), showing that it promotes recoding of UGA codons. In the presence of a downstream SECIS element, Sec insertion is enhanced. In its absence, a Sec-independent readthrough activity is still observed. We hypothesize that HRE of *SPS1-UGA* has a similar activity. In further support of this, we used RNAz (Gruber et al. 2010) to characterize the secondary structures embedded in the coding sequence of all *SPS* genes (see Methods; Supplemental Material S6). In prokaryotes, this yielded the bacterial SECIS of the Sec containing *SelD* genes (Supplemental Fig. SM6.1). In eukaryotes, we obtained stable stem–loops (SRE) in the same region of all UGA-containing *SPS* genes (Supplemental Fig. SM6.2). Strikingly, the largest and most stable structures were in *SPS1-UGA*, where we predicted HREs as a three-stem clover-like structure with the UGA in the apex of the middle stem.

Overall, these results strongly suggest that the insect *SPS1-UGA* gene is translated. Furthermore, a recent proteomics study in the hymenopteran *Cardiocondyla obscurior* (Fuessl et al. 2014) yielded a few peptides mapping to the *SPS1-UGA* gene—albeit not to the region including the UGA codon. Thus, we cannot unequivocally identify the amino acid that is inserted in response to the UGA codon. Given that we observed two independent UGA to Arg substitutions within insects, Arg could be a potential candidate. Nonetheless, the recognition of a UGA codon by a standard tRNA for Arg would require at best one mismatch in the first position of the codon (third position of the anticodon), which is expected to compromise translation.

### Functional hypothesis: Parallel subfunctionalization generates SPS1 proteins

**Figure 7.**
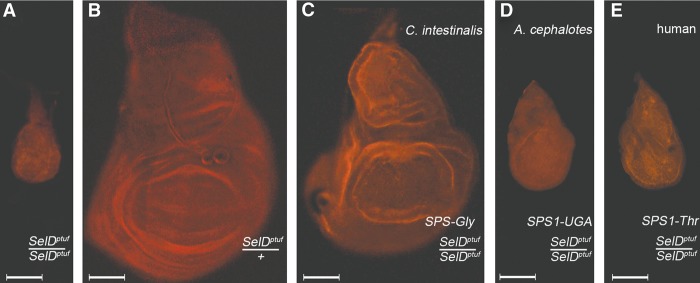
Rescue of the *Drosophila melanogaster SPS1* mutant by heterologous SPS1 proteins. All images show wing imaginal discs dissected from larvae with the indicated constructs and genotypes. (*A*,*B*) The *SPS1* mutant flies (*SelD*^*ptuf*^/*SelD*^*ptuf*^) result in defects in the whole organism, but can be easily monitored in the wing imaginal disc epithelia; the homozygous condition (*A*) strongly impairs size and morphology, whereas the heterozygous (*B*) is very similar to the wild-type condition. (*C*–*E*) The severe homozygous *SelD*^*ptuf*^/*SelD*^*ptuf*^ phenotype is partially rescued by ubiquitous expression of heterologous SPS1 proteins. (*C*) *SelD*^*ptuf*^/*SelD*^*ptuf*^ mutants with *C. intestinalis SPS-Gly*. (*D*) *SelD*^*ptuf*^/*SelD*^*ptuf*^ mutants with *A. cephalotes SPS1-UGA*. (*E*) *SelD*^*ptuf*^/*SelD*^*ptuf*^ mutants with human *SPS1-Thr*. (Scale bars) 50 µm.

Although originating from independent gene duplications of the same orthologous gene (Figs. 3, 4), the pattern of strong sequence conservation suggests that *SPS1* genes share a common function. It has been demonstrated for both insects and vertebrate *SPS1* that their function is different from *SPS2* (Persson et al. 1997; Xu et al. 2007a). We therefore suggest that the ancestral SPS2 protein at the root of metazoans had not only its catalytic activity (i.e., synthesis of selenophosphate from selenide), but also an additional, unknown function. Eventually, several metazoan lineages split these two, with a new duplicated protein, SPS1, assuming this other function. If this hypothesis is true, then SPS1 proteins (although paraphyletic) should have similar functions. To test this hypothesis, we designed rescue experiments in *Drosophila melanogaster.* The *SPS1* mutation (*SelD*^*ptuf*^) is lethal in homozygous larvae and results in very reduced and aberrant imaginal disc epithelia (Fig. 7A; Alsina et al. 1998). Thus, we used the Gal4-UAS system to activate different metazoan *SPS1* genes in *SelD*^*ptuf*^ mutants and tested whether the imaginal disc phenotype could be reverted. We drove expression of either the human *SPS1-Thr*, the *SPS-Gly* isoform from *Ciona intestinalis SPS-ae*, or the *SPS1-UGA* from *Atta cephalotes* (ant, hymenopteran), cloned downstream UAS sequences, using the ubiquitous *armadillo-Gal4* driver (Supplemental Material S7; Supplemental Fig. SM7.1). We focused on the wing imaginal disc and observed significant rescue using the *SPS-Gly* isoform from *C. intestinalis*, both in size and shape (Fig. 7C) and partial rescue in size when using the ant *SPS1-UGA* or the human *SPS1-Thr* (Fig. 7D,E). These experiments suggest that the tested heterologous SPS1 proteins have a similar molecular function, which is as previously noted, distinct from selenophosphate synthesis.

### Unequal selective pressure on *SPS1* and *SPS2* genes after duplication

We observed substantial differences in the rate of nonsynonymous versus synonymous substitution (*K*_a_/*K*_s_) across metazoan *SPS* genes between the branches corresponding to *SPS1* and *SPS2* after duplication (Supplemental Material S5). Thanks to the large number of *SPS* sequences available, we could reliably quantify *K*_a_/*K*_s_ for *SPS1* and *SPS2* in vertebrates and insects, and also for their closest outgroups, which did not duplicate *SPS* (Supplemental Figs. SM5.1, SM5.2). For annelids and ascidians, with fewer sequences available, we obtained less accurate estimates (Supplemental Fig. SM5.3). After duplication, *SPS2* genes display much higher values of *K*_a_/*K*_s_ than *SPS1*. The values for the unduplicated *SPS2* genes are very similar to *SPS1*, and thus lower than *SPS2* after duplication. Although to different degrees, the trend is observed for all *SPS* duplications in metazoans. It is evident both when comparing the extant *SPS1* and *SPS2* sequences with the predicted ancestral sequence before duplication and when comparing extant sequences within the same orthologous group (Supplemental Material SM5). This results in higher sequence divergence of the duplicated SPS2 proteins with respect to SPS1 and also to SPS2 prior to duplication. Indeed, *SPS1* genes show overall higher protein sequence similarity across metazoans than *SPS2* genes after duplication (e.g., 80% versus 70% for the sequence set in Supplemental Fig. SM5.3). Moreover, the extant unduplicated *SPS2* genes exhibit stronger sequence similarity to *SPS1* genes than to *SPS2* genes after duplication. The diverse rate of protein evolution after duplication posed a major challenge for phylogenetic reconstruction, resulting in an artifact known as long branch attraction (Supplemental Material S3). The *K*_a_/*K*_s_ difference in *SPS1* versus *SPS2* is particularly pronounced for insects, likely connected to the documented degeneration of the Sec trait in this lineage (Chapple and Guigó 2008).

## Discussion

Gene duplications are generally assumed to be the major evolutionary forces generating new biological functions (Lynch and Conery 2000). However, the mechanisms by means of which duplicated gene copies are maintained and evolve new functions are still poorly understood (Innan and Kondrashov 2010). First, the evolutionary history of genes is inherently difficult to reconstruct, since numerous factors deteriorate and confound phylogenetic signals (Philippe et al. 2011). Second, assigning functions to genes is difficult. Even the very same concept of function is controversial, and there is no universal definition of what constitutes function (Kellis et al. 2014). From a pragmatic standpoint, function is often equated to some sort of biochemical activity, which in turn is intimately connected to specific experiments to measure it. However, this fails to grasp that genes work in a concerted way within a given biological context, and thus even similar biochemical activities may result in very different biological functions (phenotypes). Also, experimental validation of function is possible only for a few genes, whereas for the great majority, function is solely assigned by inference based on sequence similarity. However, there are no universal thresholds to discriminate different functions from levels of sequence divergence, since cases exist of conserved function with low sequence similarity and conversely, different functions can be carried out by highly similar sequences. For all these reasons, functional evolution of genes is particularly difficult to trace.

Here, we have investigated the evolutionary history of selenophosphate synthetase genes (*SelD/SPS*) along the entire tree of life. *SPS* constitutes a particularly appropriate gene family to investigate evolution of function. Since it is required for selenoprotein synthesis, we can monitor its function as a genomic phenotype, by inspecting genomes for selenoproteins, and for other gene markers of selenium utilization. Moreover, *SPS* is unique among all genes in that it is part of the Sec machinery, and it is often a selenoprotein itself. As a selenoenzyme, we presume that most likely its UGA-encoded Sec residue can be functionally replaced only with Cys, and we thus expect that mutations to codons other than Cys would impair its selenophosphate synthetase function.

Consistent with this, we have found that Cys- or Sec-containing *SPS* genes (to which we refer as *SPS2*) are found in all genomes encoding selenoproteins, and those metazoan genomes containing only *SPS* genes carrying amino acids other than Sec or Cys at the homologous UGA position do not encode selenoproteins. Our results suggest that the non-Cys, non-Sec *SPS* genes share a common function, likely unrelated to selenophosphate synthesis, and we collectively refer to them as *SPS1*. In *SPS* genes, thus, the amino acid inserted at this single codon position seems to determine unequivocally protein function and is intertwined to the evolutionary fate of an entire class of proteins (selenoproteins). Thanks to this feature, which may be unique among all protein families, we have been able to untangle the complex functional evolution of *SPS* genes—in particular within metazoans, where it involves a series of independent gene duplications and the associated subfunctionalization events—from an ancestral Sec carrying *SPS* gene that had both the *SPS1* and *SPS2* functions. In some ascidians, the duplication originated by selective retrotransposition of the *SPS2* isoform of a single gene encoding both *SPS1* and *SPS2* isoforms. Alternative transcript isoforms and gene duplication are considered the main mechanisms contributing to increased protein diversity. They anticorrelate at the genomic scale, so that protein families with many paralogs tend to have fewer transcript variants and vice versa (Talavera et al. 2007). It is therefore expected that some genes shifted from one strategy to the other during evolution: The function of an alternative isoform in one species would be carried by a duplicated copy of the gene in another species. However, other than the ascidian *SPS* genes reported here, only a handful of cases have been so far reported: the eukaryotic splicing factor *U2AF1* in vertebrates (Pacheco et al. 2004), the *ey* gene in *Drosophila* (also known as *Pax6*) (Dominguez et al. 2004), and the *mitf* gene in fishes (Altschmied et al. 2002). Very recently, Lambert et al. (2015) suggested that this phenomenon could be widespread during vertebrate evolution.

Gene duplication and alternative transcription differ in the efficiency with which they separate functions. Duplications fully segregate the functions to different loci, allowing both independent sequence evolution and independent regulation. In contrast, alternative transcription/splicing isoforms typically share regions of identical sequence, and generally, proximal and distal regulatory regions. Thus, when a single gene carries a dual function (even if exerted through alternative isoforms), certain sequence segments are under two simultaneous sources of selection, which may be in competition. Gene duplication and subfunctionalization provide a way to entirely escape from the adaptive conflict (Hittinger and Carroll 2007). The fact that convergent subfunctionalization duplications occurred several times independently within metazoans is suggestive of selective advantage in having the two *SPS* functions carried out by different genes. Here, we have detected duplications occurring at the root of vertebrates, within tunicates, within annelids, and at the root of insects. However the metazoan genome space remains largely unexplored, and other duplications are likely to be uncovered in the future as additional genome sequences become available.

Overall, the history of metazoan *SPS* genes contributes a striking example of how function evolves across orthologs and paralogs through a complex pattern of duplication and loss events (Gabaldón and Koonin 2013). Specifically, it constitutes a prototypical case of a particular “functional evolution path”: an ancestral state of dual/redundant function, followed by subfunctionalization through gene duplication that occurs independently in parallel lineages of descent. Analogous cases have been previously reported (e.g., the *beta catenin/armadillo* gene during insect evolution) (Bao et al. 2012). Parallel gene duplications result in a complex pattern of orthology/paralogy relationship (Gabaldón 2008). Within monophyletic lineages with both *SPS1* and *SPS2* originated by a duplication event (e.g., vertebrates), these genes are always paralogous, but both genes are phylogenetically co-orthologous to *SPS2* in lineages with a single *SPS* (e.g., flatworms). Also, these genes are phylogenetically co-orthologous to both *SPS1* and *SPS2* in other lineages with a distinct, parallel duplication event (e.g., insects). Nonetheless, if we assume that subfunctionalization occurred in the same way in the different lineages, then the *SPS1* genes generated independently can be considered functional orthologous, despite the lack of direct phylogenetic descent. In this view, it may appear counterintuitive that *SPS1* genes have replaced the Sec/Cys site with lineage-specific amino acids, which are so dissimilar. This suggests that the residue occurring at this particular position is irrelevant to SPS1 function.

After each duplication event documented here, the *SPS2* gene increased the rate of nonsynonymous versus synonymous substitution (*K*_a_/*K*_s_). This can be interpreted as a relaxation of the selective constraints acting on the coding sequence. In contrast, *SPS1* genes preserve lower values of *K*_a_/*K*_s_, comparable to the parental gene before duplication (Supplemental Material S5). It is remarkable that this trend is observed consistently throughout all metazoan *SPS* duplications, despite the vast diversity in duplication mechanisms. We must conclude that this evolutionary pattern is intimately connected to the functions of the two *SPS* copies after duplication. Intuitively, the relaxation of constraints in the *SPS2* gene after duplication is well consistent with the scenario of subfunctionalization that we propose. However, we may have expected the duplicated *SPS1* genes to also show relaxation compared to the parental gene before duplication, since the dual-function state has presumably the highest selective constraint. In contrast, the constraints acting on *SPS1* after duplication are very similar to those before duplication. These observations may suggest that the selective constraints acting on the ancestral, dual-function *SPS2* gene are imputable mainly to selection for the *SPS1* function rather than for the canonical selenophosphate synthetase activity.

Our results suggest that readthrough of the *SPS* UGA codon to incorporate amino acids other than Sec played an important role in metazoan *SPS* duplication and subfunctionalization. Indeed, we hypothesize that simultaneous to the production of canonical Sec-coding transcripts, selenoprotein genes (including *SPS2*) could produce truncated SECIS-lacking mRNAs, due to inefficient transcription, or through a regulated process, such as alternative usage of 3′ exons or polyadenylation sites. In fact, translational regulation is known to play an important role for many selenoproteins (Howard et al. 2013). In at least one case (selenoprotein S, *VIMP* gene), this is achieved by the regulated inclusion/exclusion of the SECIS element in the mature transcripts through alternative splicing (Bubenik et al. 2013). In such SECIS-lacking truncated transcripts, no Sec insertion will take place, and termination of translation at the UGA codon is the most likely outcome. However, if alternative non-SECIS-mediated readthrough mechanisms are present, other amino acids could still be incorporated in response to the UGA codon. If this amino acid is other than Sec or Cys, the resulting protein may not be able to perform its original function (as in SPS2/SPS1), but it may develop a novel one. We speculate that this was the case for the ancestral metazoan *SPS* gene, in which possibly the novel *SPS1* function emerged in a secondary, non-Sec isoform maybe produced through regulated SECIS inclusion. The readthrough enhancing stem–loop structures around the UGA codon (SRE and HRE) found in *SPS* genes support this hypothesis. These structures are particularly strong in the SECIS-lacking hymenopteran *SPS1-UGA* genes, which in addition, contain the readthrough enhancing hexanucleotide. Then, when the *SPS* gene duplicates, the SECIS is lost in the *SPS1* copy, leading to complete subfunctionalization. The whole process is best seen within insects. *Hymenoptera*, after the *SPS* duplication, lost *SPS2* when selenoproteins disappeared from the genome, but still conserved *SPS1-UGA*. Paraneopterans with selenoproteins (*R. prolixus* and *P. humanus*) still maintain the two genes (*SPS1* and *SPS2*) with UGA. In pea aphid and in nonhymenopteran *Endopterygota*, the in-frame UGA in *SPS1-UGA* mutated to an Arg codon, becoming the “standard” gene known as *Drosophila SPS1*.

It is tempting to speculate that the SRE/HRE stem–loop structures, which are found not only in *SPS*, but also in some other selenoprotein genes (Howard et al. 2005), are the eukaryotic homologs of the bacterial SECIS element (bSECIS) (Supplemental Figs. SM6.1, SM6.2). Indeed, the bacterial Sec insertion system is different from its eukaryotic counterpart, both regarding the structure and the localization of the SECIS element. In eukaryotes, the SECIS is characterized by a kink-turn core, and it is located in the 3′ UTR. In bacteria, the bSECIS is a simple stem–loop structure (lacking the kink-turn motif), located immediately downstream from the Sec-UGA within the coding sequence. bSECIS elements are read by SelB, a Sec-specific elongation factor with a specialized N-terminal domain. In eukaryotes, SECIS elements are bound by SECIS binding protein 2 (SBP2), which recognizes specific structural features mainly around its kink-turn core (Krol 2002).

The evolutionary history of the SECIS elements across the domains of life remains largely unexplored. The assumption that SECIS and bSECIS are phylogenetically homologous structures requires the relocation of the bSECIS to the 3′ UTR, concomitant with the radical alteration of its structure—for both of which it is difficult to postulate plausible evolutionary mechanisms. In contrast, SRE/HRE are stem–loop structures that localize next to the UGA codon and resemble much more the bSECIS structure than does the SECIS. In addition to structural similarity, bSECIS and HRE/SRE also share functional similarity, since both disfavor termination at Sec-UGA sites during translation. Thus, we hypothesize that the SRE/HRE structures (at least those in *SPS* genes) are derived from bSECIS, and the eukaryotic SECIS is an evolutionary innovation unconnected to the bSECIS. After the emergence of the eukaryotic SECIS system, the ancestral bSECIS function was “downgraded” to helper for Sec insertion (SRE). In ancestral metazoans, it was kept under selection to allow both Sec-insertion and non-Sec readthrough, as the non-Sec isoform acquired the *SPS1* function. Within insects, after the *SPS* duplication, the ancestral bSECIS structure remained in one of the duplicated copies (*SPS1-UGA*), specializing only in non-Sec readthrough. This structure has been conserved in hymenopterans and some paraneopterans, becoming what we named here HRE.

In summary, owing to the singular feature that the amino acid occurring at a single position serves as binary indicator of gene function, we traced the evolutionary history of *SelD/SPS* genes with unprecedented detail, providing at the same time a survey of selenium utilization traits across the entire tree of life. In metazoans, the *SPS* phylogeny constitutes a prototypical case of functional evolution, in which dual function is segregated to different loci through independent gene duplications and subsequent convergent subfunctionalization.

## Methods

### Gene prediction

We performed gene prediction using Selenoprofiles ver. 3.0 (Mariotti and Guigó 2010) (http://big.crg.cat/services/selenoprofiles). This program scans nucleotide sequences to predict genes belonging to given protein families, provided as amino acid sequence alignments (profiles). When searching for a selenoprotein family (at least one sequence with a Sec residue), the program can find both selenoprotein genes and homologs with other amino acids at this position, as long as their full protein sequence is similar enough to the input profile alignment. To ensure maximum sensitivity, two manually curated profiles were used for *SPS*, one containing sequences from all lineages and another only from prokaryotes. Specificity was controlled through a set of filters applied to predicted candidate genes, which include checking similarity to the profile sequences and best matches among all known proteins in the NCBI nonredundant (NR) database (AWSI score and tag score; see Selenoprofiles manual). We built our *SPS* gene data sets (available in Supplemental Material S7) searching a large collection of eukaryotic (505) and prokaryotic genomes (8263 with a nonredundant reference subset of 223), downloaded mainly from NCBI. For eukaryotes and for prokaryotes in the reference set, results were manually inspected and filtered to exclude duplicates, pseudogenes (abundant in vertebrates), and contaminations of the genome assemblies. Eukaryotic SECIS elements were searched using the program SECISearch3 (Mariotti et al. 2013).

We also used Selenoprofiles with profiles derived from protein families that are markers for other selenium utilization traits, *ybbB* and *SelA*. We used the same program with a comprehensive collection of selenoprotein families in a semiautomatic procedure to probe the number of selenoproteins per lineage (as those displayed in Figs. 1, 2). tRNAscan-SE ver. 1.23 (Lowe and Eddy 1997) and Aragorn ver. 1.2.28 (Laslett and Canback 2004) were used to search for *tRNAsec*. We noticed the presence of abundant false positives in prokaryotes, lacking the long extra-arm characteristic of *tRNAsec*. Thus, we focused most of our analysis on the reference set, manually inspecting candidates and filtering out all such cases.

For ciliates, all predictions were manually adjusted, given their nonstandard genetic code. In addition to genomes, the NCBI EST database was also used to investigate certain eukaryotic lineages of interest, such as tunicates, annelids, and birds (Supplemental Materials S3, S4).

### Phylogenetic analysis

Alignments were computed using T-Coffee ver. 8.95 (Notredame et al. 2000) and sometimes complemented by MAFFT ver. 7.017b (Katoh et al. 2005). To deduce the phylogenetic history of *SPS*, we combined three types of information: topology of gene trees reconstructed from protein sequences, phylogenetic tree of investigated species, and positions of introns in respect to protein sequence. Gene trees were computed by maximum likelihood as explained in Mariotti et al. (2012) after Huerta-Cepas et al. (2011), and visualized using the program ETE ver. 2 (Huerta-Cepas et al. 2010). The approximate phylogenetic tree of investigated species was derived from the NCBI taxonomy database (Sayers et al. 2009) and was refined for insects with data from the International Aphid Genomics Consortium (2010). Figures 1 and 2 and Supplemental Figure SM1.1 were generated with the R package ggsunburst, available at http://genome.crg.es/~didac/ggsunburst/. Relative positions of introns were visualized using selenoprofiles_tree_drawer (available within Selenoprofiles) and ETE.

The *SPS* phylogenetic history presented here has been deduced using parsimony as the main driving principle. Supplemental Material S3 contains a detailed description of the process to solve the phylogeny of eukaryotic *SPS*. Supplemental Material S4 is dedicated to *SPS* genes in tunicates.

### Evolutionary analysis

We analyzed the ratio of nonsynonymous versus synonymous substitution rates (*K*_a_/*K*_s_) in metazoan *SPS* genes using the program Pycodeml (M Mariotti, unpubl.) available in Supplemental Material S8, or online at https://github.com/marco-mariotti/pycodeml. This program internally runs CodeML, a program which is part of the PAML package (Yang 2007) to predict the sequence at ancestral nodes. Then, it computes various indexes of sequence evolution related to *K*_a_/*K*_s_, fully explained in Supplemental Material S5. Pycodeml can also produce a graphical output including the sequence alignment, allowing the detailed inspection of any substitution (available at http://big.crg.cat/SPS). We have run Pycodeml on three manually curated alignments of coding sequences: one for the insect *SPS* duplication (Supplemental Fig. SM5.1); one for the vertebrate *SPS* duplication (Supplemental Fig. SM5.2); and one “summary set” containing representatives for all four *SPS* duplications here described (Supplemental Fig. SM5.3). Each alignment was trimmed manually to leave out the N-terminal and C-terminal tails and to remove any insertion that occurs only in any single sequence. Supplemental Material S5 contains the results of the evolutionary analysis, and an explanation of all sequence statistics applied.

### Detection of extensions and fusions

We used two different strategies to detect additional domains in *SPS* genes. First, we searched for annotated *SPS* fusions. We ran our *SPS* gene set with *blastp* (Altschul et al. 1997) against the NCBI NR database. Then we parsed the results, searching for large stretches of sequence of a matched NR protein that were not included in the output BLAST alignment. Second, we looked in genomes for any *SPS* extension. We expanded each predicted *SPS* gene at both sides until a stop codon was reached, and we ran the extensions with *blastp* against the NCBI NR database. All candidates from the two methods were merged, clustered by similarity, and manually inspected. Conservation in multiple species and confirmation with RNA data were used as criteria to exclude artifacts possibly caused by our detection method or by imperfect genome assemblies, thus prioritizing specificity over sensitivity. For the most interesting cases, a new alignment profile was built including the protein sequence of *SPS* and of the additional domain, and used to search again the genome sequences. Supplemental Material S2 contains a description of results.

### Prediction of conserved secondary structures

The program RNAz ver. 2.1 (Gruber et al. 2010) was used to predict conserved secondary structures embedded in *SPS* coding sequences. Initially, we produced a “master alignment” that included the coding sequences of all *SPS* genes in our data set. The nucleotide sequence alignments used were based on the alignment of the corresponding amino acid sequences. Then, the master alignment was used to extract a multitude of “subset alignments,” which included only genes in specific lineages and/or only specific types of *SPS* (based on the residue found at the Sec position). RNAz was run either directly on these subset alignments, or instead the program trimAl ver. 1.4 (Capella-Gutiérrez et al. 2009) was used in advance to reduce the number of sequences. All secondary structures predicted in this way were then manually inspected. For the best candidates, images of consensus structures were generated using the ViennaRNA package (Supplemental Figs. SM6.1, SM6.2; Lorenz et al. 2011). Supplemental Material S6 contains a detailed description of the procedure and of the results, including the list of subset alignments considered.

### Rescue experiments in *Drosophila*

For rescue experiments, we used the Gal4/UAS system (Brand and Perrimon 1993). We obtained cDNA for human *SPS1* (i.e., *SEPHS1*) from the Harvard resource core (http://plasmid.med.harvard.edu/PLASMID/). For *SPS-ae* of *C. intestinalis*, we obtained the cDNA corresponding to the Gly isoform by performing targeted PCR on larvae extracts. We obtained cDNA for *SPS1-UGA* from *A. cephalotes* by performing targeted PCR on extracts provided by James F.A. Traniello. These cDNAs were cloned through the Gibson method (Gibson et al. 2009) into the *pUAST-attB* vector linearized by double digestion with BglII and XhoI and clones verified by Sanger sequencing. Primers used for cloning are reported in Supplemental Material S7. Transgenic flies were obtained following the method described by Bischof et al. (2007). Line y*w M{e.vas-int.DM}ZH-2A; 3: M{RFP.attP}ZH-86Fb* was used to direct the insertion into the 3R Chromosome (86F). Crosses were designed to obtain expression of each transgenic *SPS1* into a *SelD*^*ptuf*^ homozygous mutant background (Supplemental Material S7; Supplemental Fig. SM7.1). We used *armadillo*-Gal4 (*arm-Gal4*) as a driver to activate expression of the *UAS*-cDNA inserts. The final cross was: *SelD*^*ptuf*^/CyOdfYFP; *UAS-*cDNA insert/MKRS × *SelD*^*ptuf*^/CyOdfYFP; *arm-Gal4*/TM6B. Expression of transgenes under *arm-Gal4* promoter was confirmed by RT-PCR (Supplemental Fig. SM7.2). Imaginal wing discs from third instar larvae were dissected in PBS and stained with Rhodamine Phalloidin from Molecular Probes (catalog #R415).

## Supplementary Material

Figures 1 and 2_Poster

Supplemental Material
