# Supplementary material for: Evolution of selenophosphate synthetases: emergence and relocation of function through independent duplications and recurrent subfunctionalization
Source: Genome Res. 2015 Sep;25(9):1256–67. doi: 10.1101/gr.190538.115 (PMC4561486; doi:10.1101/gr.190538.115)
Supplement: Figures 1 and 2_Poster [file supp_25_9_1256__index.html]

Figures 1 and 2\_Poster 

# Evolution of selenophosphate synthetases: emergence and relocation of function through independent duplications and recurrent subfunctionalization

## Figures 1 and 2\_Poster

**Files in this Data Supplement:**

- Figures 1 and 2\_Poster
