# Supplementary material for: Evolution of selenophosphate synthetases: emergence and relocation of function through independent duplications and recurrent subfunctionalization
Source: Genome Res. 2015 Sep;25(9):1256–67. doi: 10.1101/gr.190538.115 (PMC4561486; doi:10.1101/gr.190538.115)

Evolution of selenophosphate synthetases: emergence and relocation of function through independent duplications and recurrent subfunctionalization

Mariotti et al.

http://www.genome.org/cgi/doi/10.1101/gr.190538.115

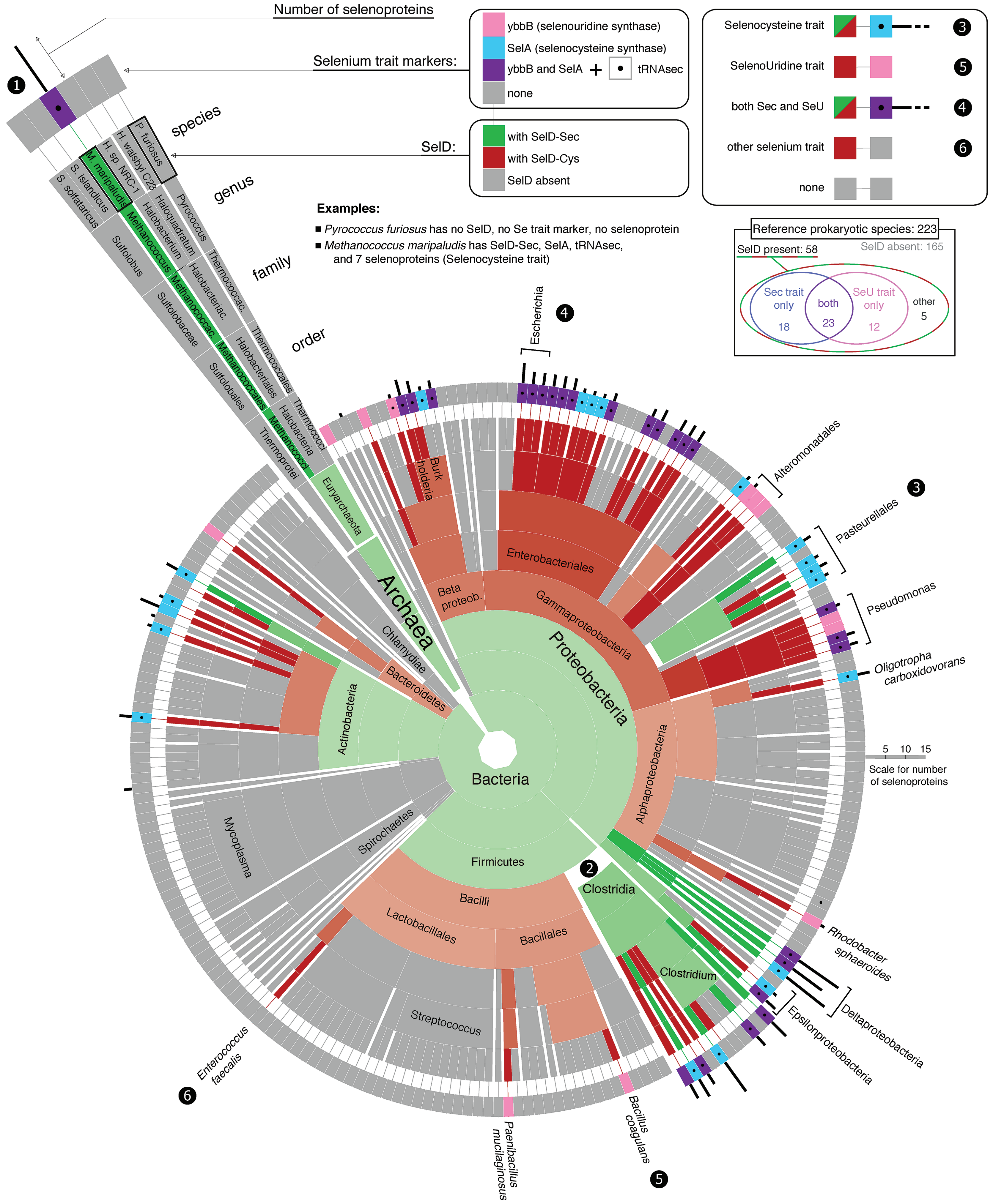

Figure 1. Phylogenetic profile of *SPS* and selenium utilization traits in prokaryotes. The sunburst tree shows the phylogenetic structure of the reference set of 223 prokaryotic genomes (taken from NCBI taxonomy) and the presence of *SelD* genes and other markers of selenium utilization. The section for archaea is zoomed in as a guide to interpret the plot (1). Every ring-shaped section represents a taxonomic rank in NCBI taxonomy (superkingdom, phylum, class, order, family, genus). The last two *outermost* rings display features for each of the species analyzed. The length of the black bars protruding from the *outermost* circle is proportional to the number of selenoproteins detected in each species. The *outermost* ring is color coded for the presence of *ybbB* and *SelA* in the species, with a black dot *inside* for denoting *tRNAsec* presence. The *second outermost* ring is labeled for *SelD* presence and type: "SelD-Sec," "SelD-Cys," "no gene found." The color is propagated to the lower ranks by hierarchy. Transparency is used to display how many species under a lineage have the same label. Assuming no Cys-to-Sec conversion and no horizontal transfer, colors reflect the predicted *SelD* presence at ancestral nodes. This allows for detecting the Sec-to-Cys conversions manually, for example in Clostridia (2). The hierarchical color assignment is violated only in the case of *Gammaproteobacteria*, altered to be red. In fact, although its sublineage *Pasteurellales* contains *SelD-Sec* (3), our analysis points to a Cys-to-Sec conversion instead, which implies that the ancestral state for *Gammaproteobacteria* was *SelD-Cys*. The plot can be used to map the Sec and selenouridine (SeU) traits (*top right* panel). For example, *Escherichia coli* (4) has both traits, *Pasteurellales* only Sec (3), and *Bacillus coagulans* only SeU (5). *Enterococcus faecalis* has a *SelD-Cys* gene but no other selenium utilization marker (6) (Romero et al. 2005; Zhang et al. 2008). Expanded versions of the plot (up to 8263 species) are available in Supplemental Material S1. Note that gene fusions and extensions are not represented in this plot.

Figure 2. Phylogenetic profile of *SPS* genes and approximate selenoproteome size of eukaryotes. The plot recapitulates the results on 505 genomes analyzed, summarized to 213 displayed here. The tree (from NCBI taxonomy) is partitioned in lineages, highlighted in gray tones to help visualization. Near the tips of leaves, the presence of SPS proteins is displayed using colored rectangles. Sec (green) and Cys (red) forms correspond to *SPS2* (*top left*), and the other homologs correspond to *SPS1* (*top right*). The gene extensions found for some *SPS2-Cys* are indicated with symbols inside the rectangle (*top left*). The number of selenoproteins predicted in each genome is indicated with a black vertical bar extending from the tips. As reference points, mammals have approximately 25 selenoprotein genes, *D. melanogaster* has three, and *C. elegans* only one.

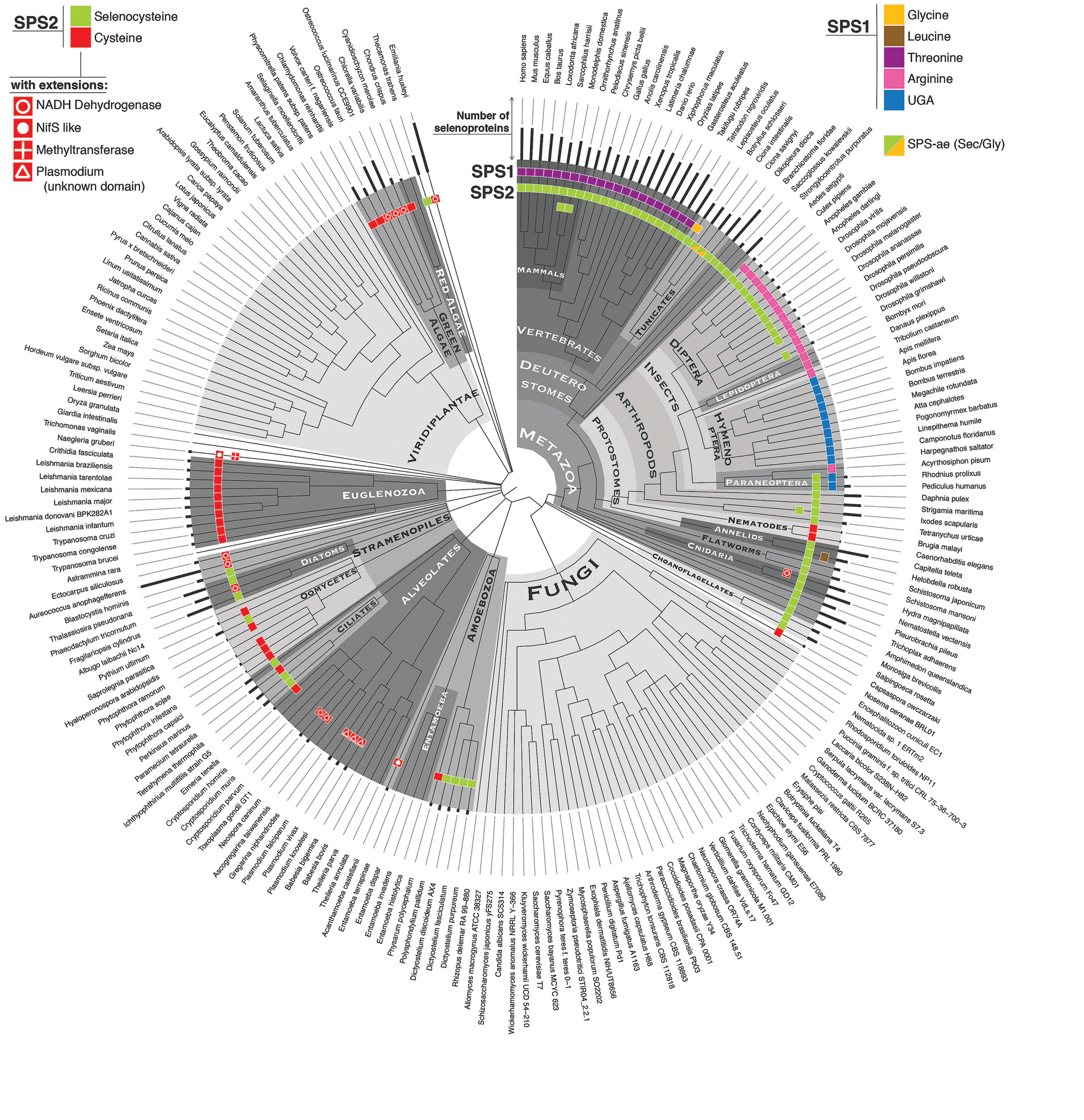

Supplement: Figures 1 and 2_Poster [file supp_gr.190538.115_Figures_1_and_2__Poster.pdf]
