# Supplementary material for: Evolution of selenophosphate synthetases: emergence and relocation of function through independent duplications and recurrent subfunctionalization
Source: Genome Res. 2015 Sep;25(9):1256–67. doi: 10.1101/gr.190538.115 (PMC4561486; doi:10.1101/gr.190538.115)
Supplement: Supplemental Material [file supp_25_9_1256_v2_index.html]

Evolution of selenophosphate synthetases: emergence and relocation of function through independent duplications and recurrent subfunctionalization — Supplemental Material 

# Evolution of selenophosphate synthetases: emergence and relocation of function through independent duplications and recurrent subfunctionalization

## Supplemental Material

**Files in this Data Supplement:**

- Supplemental Material S1.pdf
- Supplemental Material S2.pdf
- Supplemental Material S3.pdf
- Supplemental Material S4.pdf
- Supplemental Material S5.pdf
- Supplemental Material S6.pdf
- Supplemental Material S7.pdf
- Supplemental Legends.docx
- Supplemental Material S8.zip
