# Supplementary material for: Evolution of selenophosphate synthetases: emergence and relocation of function through independent duplications and recurrent subfunctionalization
Source: Genome Res. 2015 Sep;25(9):1256–67. doi: 10.1101/gr.190538.115 (PMC4561486; doi:10.1101/gr.190538.115)

Supplementary Material S1

SelD in prokaryotes

SelD gene finding in sequenced prokaryotes

We downloaded a total of 8263 prokaryotic genomes from NCBI (see Supplementary Material S7). We scanned them with the program selenoprofiles (Mariotti 2010, http://big.crg.cat/services/selenoprofiles) using two SPS-family profiles, one prokaryotic (*seld*) and one mixed eukaryotic-prokaryotic (*SPS*). Selenoprofiles removes overlapping predictions from different profiles, keeping only the prediction from the profile that seems closer to the candidate sequence. As expected, the great majority of output predictions in prokaryotic genomes were from the *seld* profile. We will refer to the prokaryotic *SPS/SelD* genes as *SelD*, following the most common nomenclature in literature.

To be able to inspect results by hand, and also to focus on good-quality genomes, we considered a reduced set of species. We took the prok_reference_genomes.txt list from ftp://ftp.ncbi.nlm.nih.gov/genomes/GENOME_REPORTS/, which NCBI claims to be a "small curated subset of really good and scientifically important prokaryotic genomes". We named this the prokaryotic reference set (223 species - see Supplementary Material S8). We manually curated most of the analysis in this set, while we kept automatized the analysis on the full set.

We detected SelD proteins in 58 genomes (26.0%) in the prokaryotic reference set (figure 1 in main paper), which become 2805 (33.9%) when considering the prokaryotic full set (figure SM1.1). The difference in proportion between the two sets is due largely to the presence of genomes of very close strains in the full set, which we consider redundant. The *Escherichia* genera in particular constitutes alone more than 7% of the genomes in the full set, and since *SelD* and selenoproteins are present in this genera, this inflates the proportion of species with *SelD*.
Generally, a single SelD protein (or none) was detected in each genome, with only a few exceptions of multiple genes (just 2 in the reference set). Only a minority of detected SelD contained selenocysteine (20.7% in the reference set, 19.3% in the full set), with the rest carrying a cysteine instead. No homologues with a different amino acid in this position were detected, at least in the prokaryotic reference set. For the full set, which includes also genomes of mediocre quality, we considered only predictions with Sec or Cys aligned to the Sec position.
In figure SM1.2, we show the reconstructed phylogenetic tree of all *SelD* genes in the prokaryotic reference set. Mostly, the topology is consistent with the phylogenetic tree of species, but with some local inconsistencies (e.g. in Xanthomonadales, Coriobacteridae, Bacillales) that suggest that some punctual events of horizontal transfer occurred; some of these will be discussed in the next chapters.

Searching other markers for selenium utilization traits

Figure 1 in the main paper shows the presence of Sec and Cys SelD proteins in the reference set of species, as a phylogenetic sunburst. As you can see, *SelD* presence is mostly scattered, highlighting a very dynamic process acting on selenium utilization traits.

To link *SelD* to its functional network, we searched our collection of genomes also for other selenium trait markers: ***tRNAsec*** and ***SelA*** (SelenoCysteine synthase/L-seryl-tRNA(Sec) selenium transferase) for the selenocysteine trait, and ***ybbB*** (tRNA 2-selenouridine synthase), for the selenouridine trait. We also predicted the selenoproteins encoded in each genome, to have an estimation of the selenoproteome size. All these searches were carried out using the program Selenoprofiles, building profile alignments on purpose when necessary. The only exception was *tRNAsec*: for this, we ran the programs tRNAscan-SE (Lowe 1997) and Aragorn (Laslett 2004). Both programs are thought for predictions of all tRNAs. Considering only *tRNAsec*, Aragorn appeared to be more sensitive, but less specific than tRNAscan-SE, but really none of the programs gave satisfactory results, mainly for the presence of false positives in many lineages. To have a reliable set of *tRNAsec* annotation we thus restricted our search to the reference prokaryotic set, and we manually inspected and filtered the predictions. We simply excluded all *tRNAsec* candidates lacking the characteristic long variable arm (Palioura 2009). We believe most of these false positives constitute real tRNAs with a UCA anticodon that can read UGA, but which do not load selenocysteine. Such tRNA predictions were present for example in all Mycoplasmas, which are known to use UGA for tryptophan. After this filtering, 43 out of 45 species with a *SelA* prediction have a tRNAsec prediction too.

The majority of investigated prokaryotic species do not possess *SelD*, and thus are expected unable to produce both selenoproteins and selenouridine containing tRNAs. Considering the continuity of the Se traits as the basic scenario (although punctual horizontal transfers have certainly occurred), this means that multiple losses of Se-traits happened along the prokaryotic tree. The selenocysteine and selenouridine trait were found to have a good overlap, with 25 species in the reference set possessing both *ybbB* and *SelA*. Selenocysteine appears to be more common than selenouridine: 20 species were found to possess *SelA* but not *ybbB*, while only 12 species possessed *ybbB* but not *SelA*.

In general, the presence of different gene markers shows consistency, so that, for example, the species for which a *tRNAsec* has been identified contain also *SelA*, *SelD* and selenoproteins. Only a few cases (reported hereafter) are not consistent. These can probably be ascribed to genome assembly errors or incompleteness.

Two species were predicted to possess *tRNAsec* but not *SelA*: *Rhodospirillum rubrum ATCC 11170* (Alphaproteobacteria, Rhodospirillales) and *Cupriavidus necator N-1* (Betaproteobacteria, Burkholderiales). While the former has no other selenium utilization trait, the latter has *SelD*, *tRNAsec* and selenoproteins. Thus, the most likely explanation for *R.rubrum ATCC 11170* is that the tRNAsec is either not a real tRNA for selenocysteine, or it is wrongly included in the assembly. For *C.necator N-1* instead, the most likely explanation is that this species has actually selenoproteins and a full Sec pathway, but *SelA* is missing from the genome for incompleteness of the assembly.

The genomes of *Amycolatopsis mediterranei U32* (Actinobacteria) and *Finegoldia magna ATCC 53516* (Firmicutes, Clostridiales) both contain selenoproteins, *SelA*, *SelD* but no *tRNAsec* could be identified. Similarly, we believe *tRNAsec* is actually present but missed just because of incomplete assemblies.

Finally, a few other species have selenoprotein predictions by Selenoprofiles but are not predicted to possess a complete Sec trait. *Propionibacterium acnes KPA171202, Bifidobacterium bifidum S17* (both Actinobacteria) and *Synechococcus elongates PCC 6301* (Cyanobacteria) have no selenium trait utilization at all, except for some selenoprotein gene candidate. *Marinobacter aquaeolei VT8* (Gammaproteobacteria, Alteromonadales) and *Rhodobacter sphaeroides 2.4.1* (Alphaproteobacteria, Rhodobacterales) instead have selenoproteins and *SelD*, but this appears to be used for

selenouridine utilization: *ybbB* is found, while *tRNAsec* and *SelA* are not. For all these species, we inspected the selenoprotein predictions by eye, and they look convincing. The most likely explanation, then, is that these genes are from contaminants and were wrongly included in the assembly.

We detected at least one selenium utilization trait in almost all reference species with a *SelD* gene, with the exception of: *Enterococcus faecalis* (described below, within Bacilli), *Atopobium parvulum DSM 20469, Clostridium saccharolyticum WM1, Eubacterium rectale ATCC 33656* (all of which with no signs of selenium utilization traits except for *SelD* presence), and the aforementioned *Amycolatopsis mediterranei U32*. Apart from *A.mediterranei,* all other species listed here above are expected to possess *SelD* for a 3rd trait selenium utilization.

Se utilization in Archaea

We had 54 archaeal genomes in our full dataset (6 in the prokaryotic reference set -- see point number 1 in Figure1). *SelD*, *SelA* and selenoproteins were found only in the two lineages: Methanococcales (*Methanocaldococcus jannaschii DSM 2661, Methanococcus aeolicus Nankai-3, Methanococcus maripaludis* strains: *C5*, *C7*, *S2*, *Methanococcus vannielii SB*) and Methanopyri (*Methanopyrus kandleri AV19*). All archaeal *SelD* forms detected were with selenocysteine. Selenoproteins were already described in these species (Rother 2003). In *M. maripaludis* we identified 7 selenoproteins, 4 of which belonging to the formate dehydrogenase family (*fdha*), one coenzyme F420-reducing hydrogenase large subunit (*frha* or *fruA*), one HesB-like selenoprotein, plus the Sec-containing *SelD*. Since for our selenoproteome size estimation we prioritized specificity rather than sensitivity, additional selenoproteins missing in our annotation are expected in archaea, as well as in other prokaryotes.
The archaean *ybbB* gene is split in two genes in comparison to bacteria, one with the rhodanese domain delivering the selenium (N-terminal in bacteria), and one with a P-loop WalkerA motif (C-terminal) (Su et al. 2012). The genes are located adjacent, on the same strand, but with inverted positions (the C-terminal domain gene is upstream). While genes similar to rhodanese-ybbB were found in other archaeal genomes lacking *SelD*, WalkerA-ybbB was found only in Methanococcales. Interestingly, it is missing in Methanopyri, which then appear to utilize selenocysteine but not selenouridine.

Sec / Cys conversions of *SelD*

Sec to Cys conversions are a process peculiar to selenoprotein genes. Cysteine codons are just one point mutation away from TGA, and cysteine and selenocysteine have similar properties. For most selenoproteins, cysteine homologues (orthologues or paralogues) are known (Fomenko 2012). Despite the fact that Sec to Cys conversions have been widely observed (see for example Mariotti 2012), no clear Cys to Sec conversion is documented in literature. Nonetheless, considering that selenoprotein families of prokaryotes and eukaryotes have little overlap (Driscoll 2004), and that some eukaryotic selenoprotein families have homologues with Cys in prokaryotes, it is natural to assume that Cys to Sec conversions have indeed occurred, generating new selenoprotein families from existing protein families (see also Zhang 2006).
In order to identify Sec / Cys conversions, we ran our phylogenetic reconstruction pipeline (see Methods) obtaining phylogenetic trees of all SelD proteins predicted in prokaryotes (figure SM1.2 shows the results on the reference set). This data, together with the species tree, allowed us to reliably trace some of these conversions events.

Dynamic evolution of Se traits in Clostridia

Clostridia are a very diverse lineage when we consider selenium utilization traits. You can see this in Figure 1 in the main paper (point number 2), or in Figure SM1.1.
Some organisms (such as *Desulfitobacterium hafniense*) possess both the selenocysteine and selenouridine trait, others (such as *Clostridium botulinum A str. ATCC 3502*) possess only the selenocysteine trait, and others again (such as *Clostridium thermocellum*) have none. Intermediate states are also sometimes found.
Within this lineage we noticed many Sec-to-Cys conversions. To investigate them in detail, we have extracted all Clostridium predictions from our prokaryotic full set, removing redundancy at the species level.
Figure SM1.3 and figure SM1.4 show respectively their predicted protein tree, and the species tree annotated with the predictions.
These indicate that the last common ancestor of this lineage had a Sec-*SelD* gene, and this was converted to a cysteine homologue many times independently in various lineages. Interestingly some of these conversions must be very recent, as for example some strains of Lachnospiraceae were found with Sec-*SelD*, and others with Cys-*SelD* (figure SM1.4).
We believe that the scattered presence of SelD proteins across all sequenced prokaryotes is the product of the same process we observe in Clostridia, with frequent Sec to Cys conversions from an ancestral Sec-*SelD* form, and also frequent gene losses (concomitant with the loss of Se traits).

Selenocysteine losses in Bacilli

Bacilli constitutes a well studied bacterial lineage (including among others *Staphylococcus*, *Streptococcus* and *Enterococcus*) that together with Clostridia forms the phylum of Firmicutes. Most Bacilli appear to lack *SelD*, and thus the selenium utilization traits. In fact, if we consider just the prokaryotic reference set (Figure 1), there are only three species with *SelD*: *Bacillus coagulans* and *Paenibacillus mucilaginosus*, both possessing the selenouridine trait, and *Enterococcus faecalis*, that do not possess neither *SelA*, *ybbB*, or the *tRNAsec*. The presence of an “orphan” *SelD* gene in this species has been previously noted (Zhang 2008, Haft 2008), and may be explained by the use of Se as cofactor to molybdenum hydroxylases (Srivastava 2011).
When we increase the number of considered species, thus increasing the resolution (see Figure SM1.1), we notice that not all Bacilli lack selenocysteine. There are several species, phylogenetically scattered, that possess either the selenocysteine trait, the selenouridine trait, or even both. The genus *Bacillus*, *Paenibacillus* and *Lactobacillus* exhibit such diversity, roughly analogous to the situation described for Clostridia.
Using the full set of 8263 prokaryotic species, only a few families of Bacilli show no presence of *SelD* at all: Leuconostocaceae, Listeriaceae, Staphylococcaceae.
The case of Streptococcaceae is bizarre, and interesting. In our full prokaryotic set we have 872 genomes belonging to this lineage, and we found *SelD* in a single species: *Streptococcus sobrinus TCI-157.* This is also the only Streptococcaceae species with any other Se marker: a bona fide *ybbB* gene was identified.
This suggests that this species truly possesses and utilizes *SelD* to produce selenouridine containing tRNAs, and that this feature is extremely rare (if not unique) in this lineage. There are two possible explanations: either selenouridine (*SelD* + *ybbB*) was lost independently in the lineages coming out from the Streptococcaceae radiation, and was kept only in this one (extremely unlikely), or most probably it was lost at the root of this family, and re-acquired just in this species by horizontal transfer.

Running blastp using *SelD* and *ybbB* from *S. sobrinus TCI-157,* we see that the most similar proteins annotated are from the genus of *Paenibacillus* or *Bacillus*, which thus are the most likely sources of horizontal transfer.

Se traits in Proteobacteria

Proteobacteria are a major group of Bacteria that contains many lineages of medical interest, as for example *Salmonella, Burkholderia, Campylobacter* and *Escherichia.* Proteobacteria constitutes the most represented phylum in our datasets, constituting 44-47% of the total number of species. The sequenced species belong to the five major classes of alpha, beta, gamma, delta and epsilon proteobacteria, described hereafter. One zetaproteobacteria species was also present in our full dataset (*Mariprofundus ferrooxydans PV-1*); it appears to lack *SelD* as well as selenoproteins and any other Se marker.

Alphaproteobacteria

As for other cases already mentioned, increasing the resolution reveals a more complex pattern in Alphaproteobacteria: compare Figure 1, generated using the reference set, with Figure SM1.1, generated using the full species set. Selenium utilization remains quite uncommon, but scattered through most Alphaproteobacteria sublineages.
The order of Rhodobacterales shows the highest diversity, with species having the Sec trait, SeU trait, both or none. In the rest of the phylum, selenium traits are much less common. Selenouridine was found only scattered through Caulobacterales, and selenocysteine only in the orders of Rhizobiales and Rhodospirillales.

Betaproteobacteria

Sec and SeU are quite common in Betaproteobacteria, although still exhibiting a diversified pattern that testifies the dynamic process acting on these traits. Most Burkholderiales sublineages possess *SelD* and at least one complete Se trait. The genus of Burkholderia itself shows a recent dynamic evolution, with closely related species that differ for the presence of Se utilization traits.
Within the order of Neisseriales, *SelD* and Se traits (both Sec and SeU) are found only in few species in our dataset (*Laribacter hongkongensis HLHK9*, *Chromobacterium sp. C-61, Pseudogulbenkiania ferrooxidans* and *Pseudogulbenkiania sp. NH8B*).

Gammaproteobacteria: a Cys to Sec conversion in Pasteurellales

Gammaproteobacteria are a class of bacteria that contains many important human pathogens, including among others the genus *Escherichia*, *Salmonella* and *Pseudomonas*. This class is well represented in our sequence datasets, with 49 species in our reference set, 2540 in our full set (the best represented proteobacteria order). *SelD* proteins were detected in the majority of Gammaproteobacteria (57% of species in reference set, 63% in full set).
The SeC trait was identified in the vast majority of Enterobacteriales (including *Escherichia, Yersinia, Salmonella, Shigella, Enterobacter*). SeU is also found in the same lineages, with the only notable exception of the *Yersinia* genus, that apparently lost SeU but kept Sec.
The majority of species in the family of Pseudomonadaceae (including *Pseudomonas*) possess *SelD*, with either both Sec and SeU, or just SeU, apparently important for this lineage. In contrast, its sister family Moraxellaceae exhibits no *SelD*, no *ybbB*, no *SelA* and no selenoproteins, indicating a complete loss of known Se utilization pathways.

SelD is quite uncommon in the order of Xanthomonadales, where it was found only among *Stenotrophomonas,* and also in the species *Wohlfahrtiimonas chitiniclastica SH041* and *Dyella japonica A8.*
Intermediate states were found in the orders of Alteromonadales and Oceanospirillales, both exhibiting a diversified, scattered pattern with species possessing mostly SeU, both SeU and SeC, or none.

We were surprised to see a very low number of Sec containing SelD proteins in Gammaproteobacteria (4-7% of total; species are underlined here after). Most of them were found in the family of *Pasteurellales,* where the majority of *SelD* are with Sec, although a very few Cys-*SelD* were also identified (e.g. *Gallibacterium anatis UMN179*). Then, the rest of Gammaproteobacteria Sec-*SelD* were found only in very narrow lineages: in some *Photobacteria* (Vibrionales), in species *Allochromatium vinosum* (Chromatiales), and in species *Wohlfahrtiimonas chitiniclastica* (Xanthomonadales).
Given the rich sampled diversity with the Gammaproteobacteria genomes, and the extremely low number of Sec-*SelD* forms, it is natural to think that their last common ancestor contained a single Cys-*SelD* gene.
Thus, Sec-SelD proteins may have arisen in the lineages mentioned above by one of two possible mechanisms: horizontal gene transfer (HGT) of a Sec-*SelD*, or conversion of Cys-*SelD* to selenocysteine.
To investigate this, we extracted all Gammaproteobacteria *SelD* genes from our full set of predictions. We then narrowed the set by removing sequence redundancy, that is to say, keeping only one representative for each cluster of almost identical (>95%) protein sequences. In this process, we took care that no Sec protein was dropped in favor of a Cys containing representative. We then ran our phylogenetic reconstruction pipeline on this protein dataset. Figure SM1.5 shows the predicted protein tree topology.
Additionally, to control for HGT, we have ran blastp for each Gammaproteobacteria Sec-*SelD*, searching for the closest related sequences outside its taxonomic order. So for example we have run the Sec-*SelD* of *Photobacterium profundum SS9* against the whole set of annotated proteins (nr), excluding those belonging to any Vibrionales. Below, we report our conclusions.

The Sec-SelD proteins found in some *Photobacteria* (*P. profundum SS9, P. profundum 3TCK, P. sp. AK15*) appear to be product of horizontal transfer. In fact, the most similar proteins annotated in nr belong to very distant species (Firmicutes, or Chloroflexi). Most notably, these Sec-*SelD* do no cluster with the rest of Vibrionales sequences (see figure SM1.5), falling very far from the (Cys-containing) *SelD* found in other *Photobacteria.*
Allochromatium vinosum Sec-*SelD* most probably comes from another horizontal transfer.
The most similar sequences returned by blastp all belong to the lineages of Firmicutes. Actually, the whole set of Chromatiales *SelD* sequences do not cluster together, but rather form small genus specific clusters, which suggests that even the Cys-forms may have been acquired by multiple horizontal transfers.
Wohlfahrtiimonas chitiniclastica also appears to have acquired Sec-*SelD* by horizontal transfer. While the rest of Sec-*SelD* sequences nicely cluster together in the protein phylogeny (figure SM1.5), *W. chitiniclastica* Sec-*SelD* clusters instead with Pseudomonadales sequences. Blastp also returns proteins from those lineages as the most similar to this query. There is an apparent paradox with this: we could not find any Sec-containing *SelD* in Pseudomonadales, only Cys forms, despite a good representativity in our dataset. This means that either 1. the source of the horizontal transfer is a species from a unknown, Sec-*SelD* containing bacterial lineage which is not sequenced yet, whose closest relative in our datasets is Pseudomonadales, or 2. the original *SelD* gene

transferred was with cysteine, and was converted to Sec during, or shortly after, the transfer.

Finally, we think that Pasteurellales gained Sec-*SelD* by a cysteine to selenocysteine conversion of their existing Cys-*SelD*. In fact, all their SelD protein sequences (both Sec and Cys containing, found in different species) form a unique similarity cluster (see figure SM1.5). The most similar sequences found in other taxonomic orders (both by blastp and in our protein tree) are from Enterobacteriales, the closest related order to Pasteurellales (Gao 2009). Thus, the most likely scenario involves a Cys to Sec conversion in the *SelD* gene in the last common ancestor of Pasteurellales. Then, the codon switched back to cysteine in several lineages independently (e.g. *Haemophilus parasuis*). The conversion apparently did not alter selenium utilization in this lineage, which appears to be uniformly using selenocysteine only (a few species are exceptions in that they do not use selenium at all, having lost *SelD*).

Concluding, we found in Pasteurellales the first well supported Cys to Sec conversion ever documented. In one such event, it is of key importance that a functional bacterial SECIS element is established at the time of the mutation that originates the TGA. In this case, this was probably favored by the biased sequence composition of this gene region, for it had already contained a bacterial SECIS once (parsimoniously, we assume the presence of a Sec-*SelD* gene in the last universal common ancestor).

Deltaproteobacteria are selenoprotein rich

The majority of Deltaproteobacteria were predicted to possess a Sec-*SelD* gene, a complete Sec machinery, and plenty of selenoproteins. Species *Desulfobacterium autotrophicum HRM2* exhibited the largest predicted selenoproteome among prokaryotes: we found 31 selenoprotein genes, belonging to 18 distinct protein families.
Some Deltaproteobacteria appeared to possess both the Sec and SeU traits (e.g. *Geobacter*). Only a few possessed SeU but not Sec (e.g. *Bdellovibrio*).

Epsilonbacteria

Sequenced Epsilonbacteria belong mainly to two families: Campylobacteraceae and Helicobacteraceae. The former appear to possess both Sec and SeU, and several selenoproteins were predicted in their genome. In contrast, we found two distinct situations for Helicobacteraceae. Certain species possess a complete Sec machinery, with also a few selenoproteins predicted in their genome , and can either have also SeU (e.g. *Helicobacter pullorum*) or not (e.g. *Helicobacter hepaticus*).
The rest of species, which actually form the majority of Helicobacteraceae (including *Helicobacter pylori*), are predicted to possess no selenoprotein and no *SelD*. Surprisingly, for most of them we predicted a *SelA* gene in the genome. Given the absence of *SelD* and of predicted selenoproteins in these genomes, we think that this protein may have been readapted to a different function.

Actinobacteria

This class of bacteria also exhibited a highly scattered pattern of Se traits, testifying a very dynamic evolution. The gene *ybbB* was not found in any species in this lineage, and therefore we expect SeU not to be utilized. *SelD* was found only in ~19% of species in our full dataset, scattered through sublineages (see figure SM1.1); 87% of these species possessed *SelA*, and 94% had at least one selenoprotein predicted in the genome. So, it appears that this pattern is the product of a real process of Sec loss acting on parallel

lineages. The genus *Mycobacterium* showed a remarkable diversity in this, with only ~19% of these species possessing *SelD* and selenoproteins.
On a total of 140 SelD proteins predicted in Actinobacteria, only 11 carried selenocysteine. All Sec-*SelD* were found within the order of Coriobacteriales, with the exception of species *Kineosphaera limosa NBRC 100340* and *Rubrobacter xylanophilus DSM 9941*.

Other bacterial lineages

We here provide a short report on the rest of prokaryotic taxonomic classes present in our dataset.
Cyanobacteria appear to have lost Sec: *SelA* was not found in any of these genomes, and a very limited number of selenoprotein genes were predicted. At manual inspection, most of them appeared to be false positives. Nonetheless, ~39% of Cyanobacteria were predicted to possess *SelD* (always with Cys). *ybbB* was identified in 92% of the *SelD* containing species, indicating that some Cyanobacteria retained *SelD* to produce SeU-containing tRNAs.
Bacteroidetes exhibit a similar pattern, with few species conserving *SelD* as part of the SeU trait. *SelA* is not found in any genome, with the only exception of *Chryseobacterium taeanense*, which carries a gene almost identical to *SelA* as found in the Betaproteobacteria genus *Delftia*. Interestingly a Sec containing formate-dehydrogenase was found in the same genome. This potentially supports a second acquisition of the Sec trait in *C. taeanense* by horizontal transfer; nonetheless, given that we observe this in a single genome, we cannot exclude that the genes are actually from a contamination introduced in the sequencing process.
Spirochaetes show a scarce presence of Se traits. Using the reference set (figure 1) this lineage appeared to completely lack *SelD*, but with more resolution (figure SM1.1) we can notice this is not the case. *SelD* was found in a limited number of species (e.g. *Brachyspira pilosicoli*) apparently to produce selenocysteine. Sec-*SelD* genes were also detected, uniquely in the genus *Treponema*.
Lastly, Chlamydiae were found devoid of *SelD*, *SelA* and *ybbB*, indicating a complete loss of Se utilization. Tenericutes (including Mycoplasmas) are also predicted to lack all Se traits.

Figures in Supplementary Material S1:

NOTE: we suggest to download the large figures from big.crg.cat/SPS and visualize them on screen to allow readability.

Figure SM1.1:

Sunburst tree of *SelD* and other Se-trait markers in the full set of prokaryotes. See caption of Figure 1 in the main paper (reference set) for explanations.

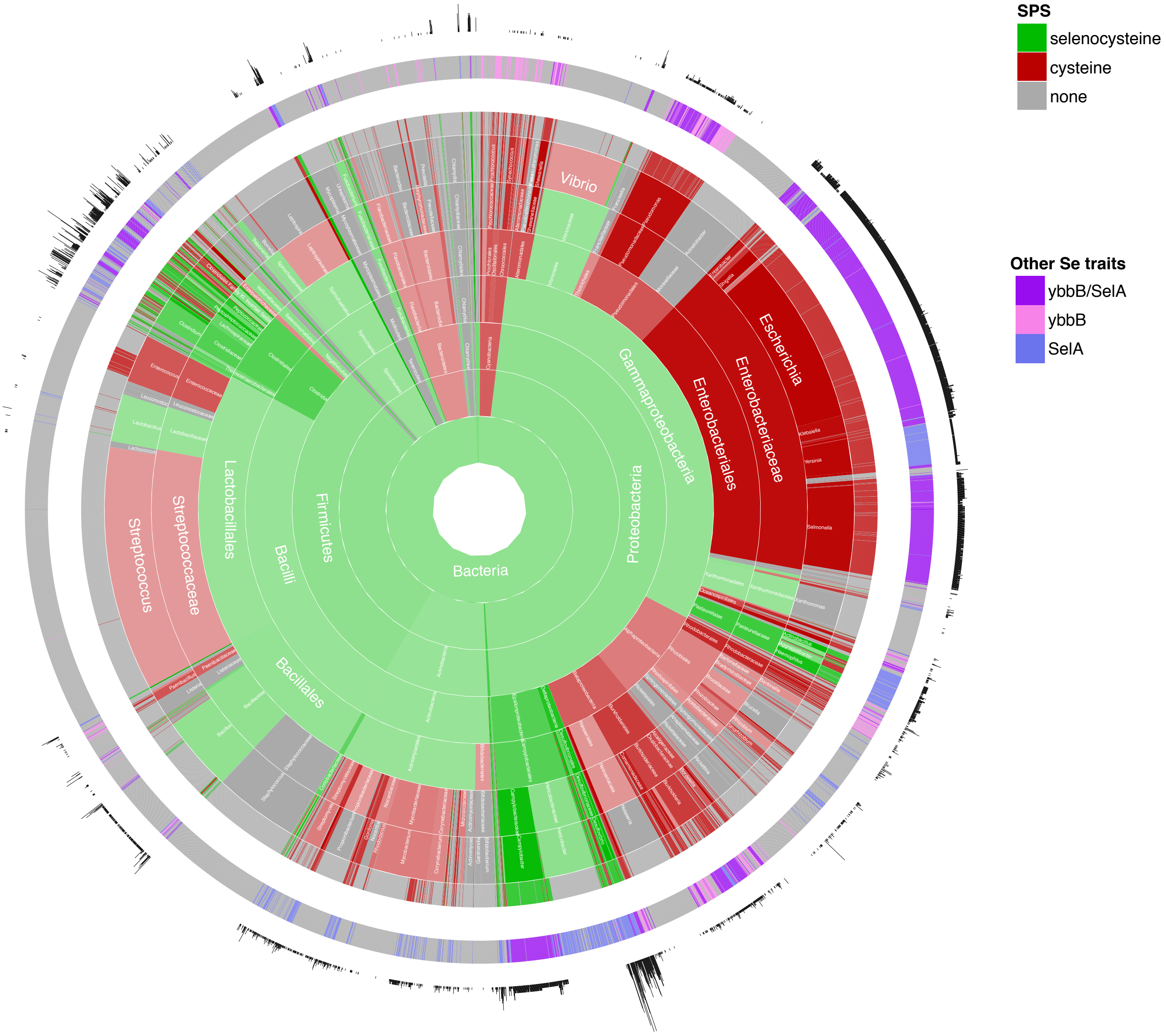

Phylogeny of Selenophosphate synthetases (SPS)

Figure SM1.2:
Reconstructed protein phylogeny of all *SelD/SPS* proteins in the reference prokaryotic set.

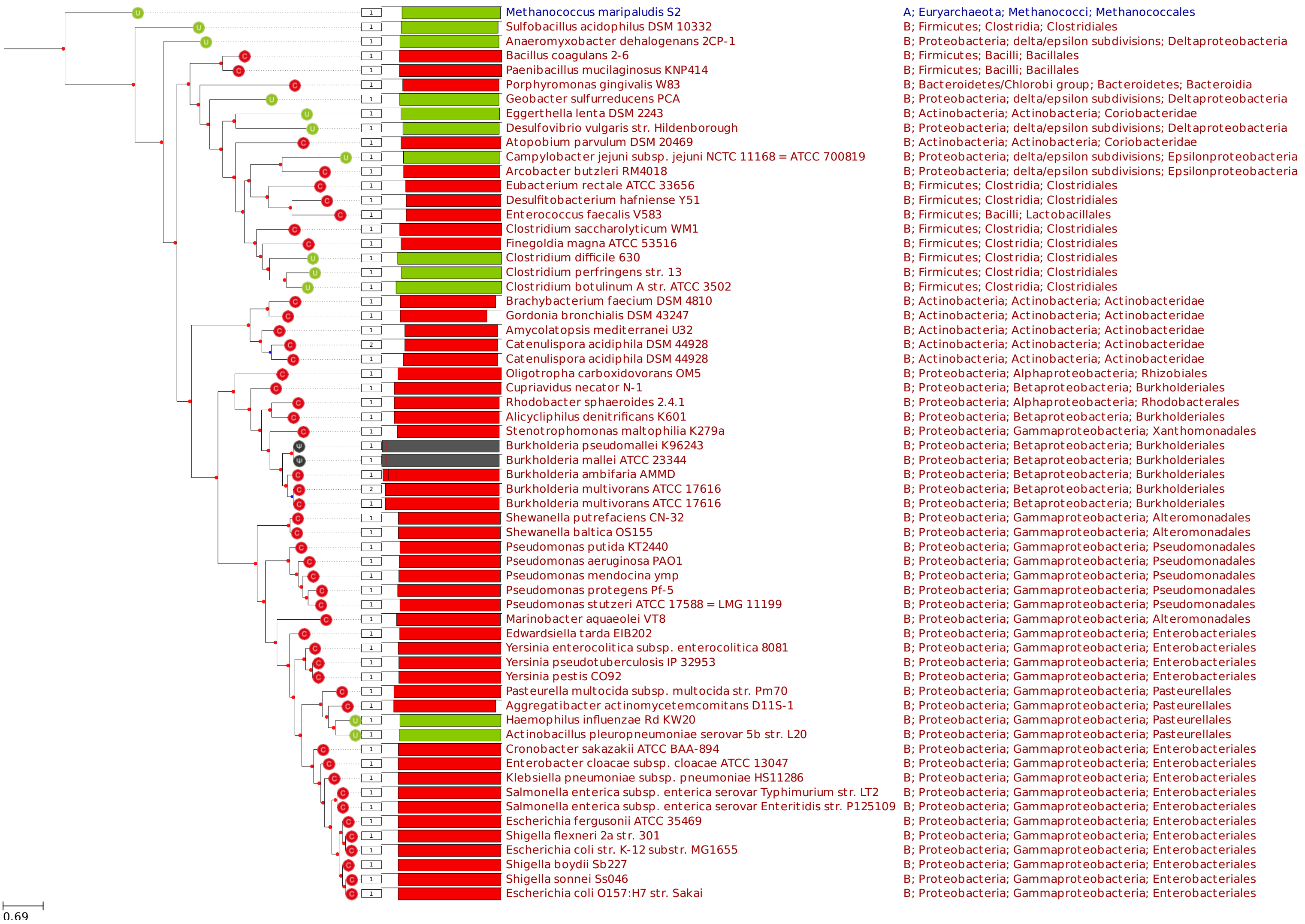

Figure SM1.3: (next page)
Reconstructed protein phylogeny of all *SelD/SPS* proteins predicted in Clostridia.
For full readability download this from [big.crg.cat/SPS](big.crg.cat/SPS) and visualize on screen.

Phylogeny of Selenophosphate synthetases (SPS)

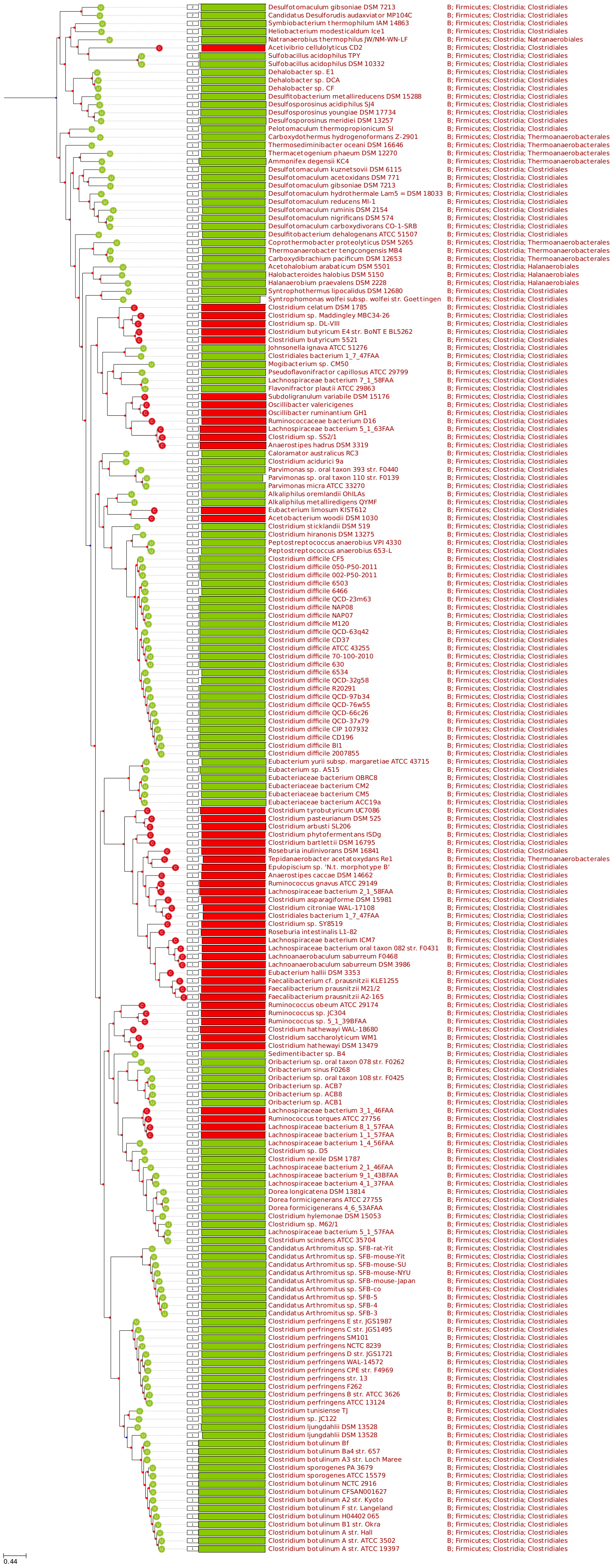

Phylogeny of Selenophosphate synthetases (SPS)

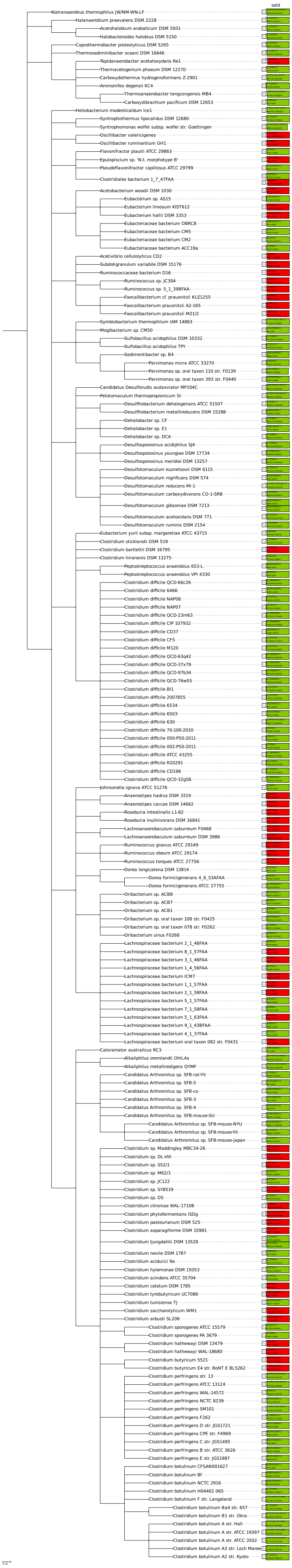

<u>Figure SM1.4:</u>
Clostridia species tree annotated with SelD/SPS proteins identified in each genome. Genomes with no results are not displayed here.
For full readability download this from big.crg.cat/SPS and visualize on screen.

<u>Figure SM1.5:</u> (next page)
Reconstructed protein phylogeny of all SelD/SPS proteins predicted in Gammaproteobacteria. See notes in the text about Sec containing *SelD* genes.
For full readability download this from big.crg.cat/SPS and visualize on screen.

Phylogeny of Selenophosphate synthetases (SPS)

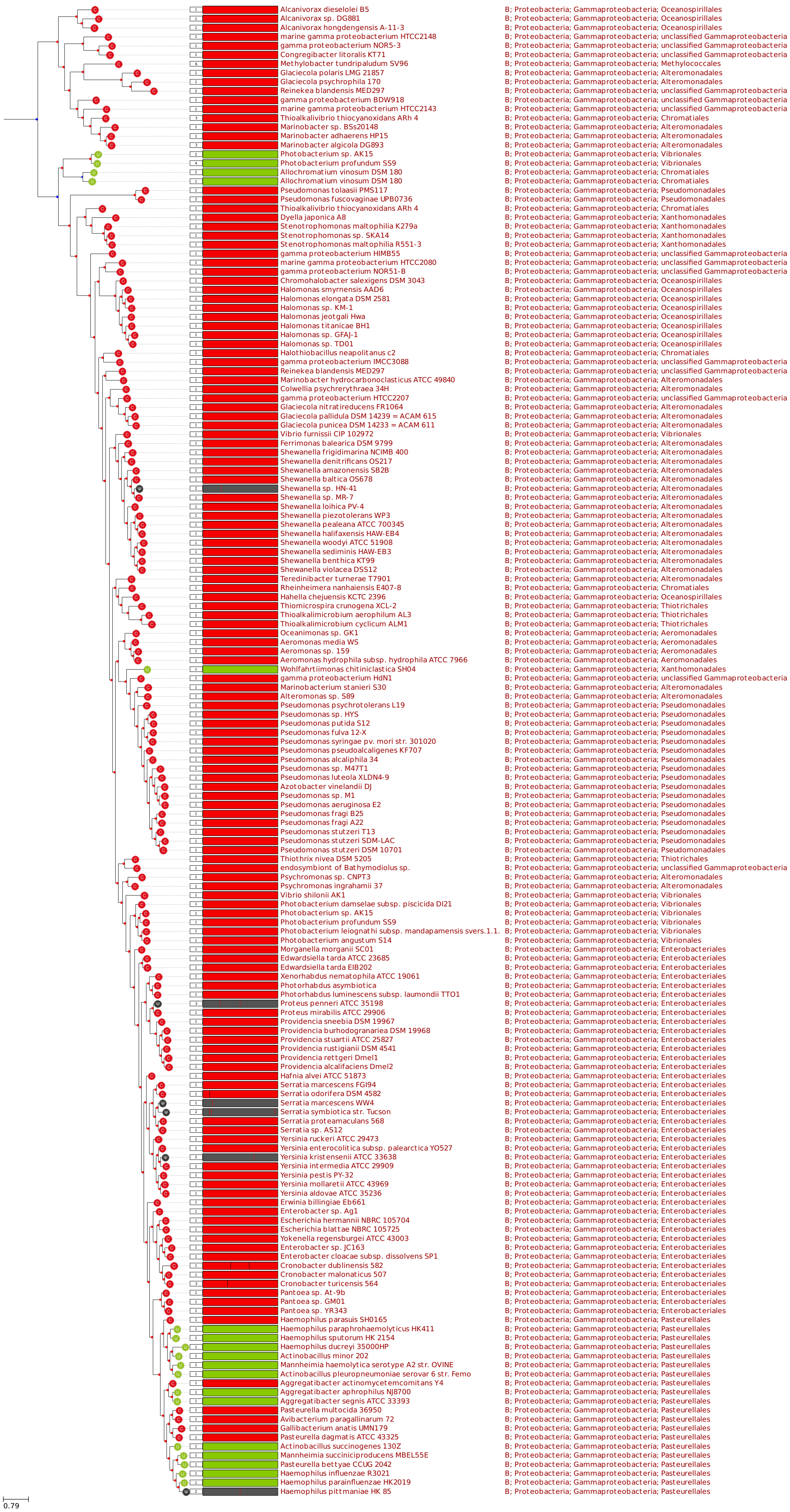

Supplement: Supplemental Material [file supp_gr.190538.115_Supplemental_Material_S1.pdf]
