# Supplementary material for: Evolution of selenophosphate synthetases: emergence and relocation of function through independent duplications and recurrent subfunctionalization
Source: Genome Res. 2015 Sep;25(9):1256–67. doi: 10.1101/gr.190538.115 (PMC4561486; doi:10.1101/gr.190538.115)

Supplementary Material S2:

Gene fusions and extensions

Some gene fusions were previously reported for *SPS*: with a NADH-dehydrogenase domain in bacteria and some protist eukaryotes (Zhang 2008), with a Cys sulfinate desulfinase / NifS protein in *Geobacter sp. FRC-32* (Zhang 2008). Recently, species *Naegleria gruberi* (Da Silva 2013) was reported to possess a gene product of the fusion of a SPS protein with a methyltransferase protein. Through genetic experiments, authors show that the fused protein still performs the canonical *SPS* function (SeP production). The N-terminal probably possess an additional function. It is possible that this is related to a detoxification process, as authors found that this domain conferred partial resistance to selenium toxicity (see Da Silva 2013). Thus, we searched for possible protein extensions or fusions to other genes in our *SPS* prediction dataset (see Methods).

A unique methyltransferase-SPS fusion in *Naegleria gruberi*

The *N.gruberi SPS* gene fusion described in literature was detected by our procedure. The N-terminal showed homology to proteins arsenite methyltransferase, UbiE/COQ5 methyltransferase, methyltransferase type 11. The C-terminus appears to be a complete *SPS* gene, with a cysteine aligned to the usual Sec position.
We were surprised to find this fusion uniquely in species *N.gruberi*. Nonetheless, it must be noted that this taxonomic group (heterolobosea) is scarcely sequenced to date.
Interestingly, we noted that arsenite methyltransferases includes some selenoproteins in bacteria (see Zhang 2008), suggesting a functional link between the two domains.
From experiments in (Da Silva 2013), it is most likely that this fused protein possesses an additional, rather than an alternative, function. In fact, the fused protein (or even only its SPS domain) is able to complement a *SelD* deficiency in *Escherichia coli*. This is consistent with the identification of other selenocysteine machinery proteins in *N. gruberi* by the same authors, which advocates for the ability of *N. gruberi* to code selenocysteine. Nonetheless, a single selenoprotein was identified in (Da Silva 2013): a thioredoxin reductase with homology to mammalian TR3.
Using the selenoprotein prediction tools that we developed in the last years (Mariotti 2010, Mariotti 2013), we could predict two additional selenoproteins in this genome: a second thioredoxin reductase, and a deiodinase-like protein. Also, we found a second cys-containing *SPS* gene in this genome, unreported in (Da Silva 2013). To our increasing surprise, we noted that this gene is also the product of a fusion: the C-terminal has homology to SPS, while the N-terminal is homologous to NifS proteins.

NifS-SPS fusions

The Cys sulfinate desulfinase (*NifS*) proteins process indiscriminately cysteine or selenocysteine, producing alanine and elemental sulfur or selenium respectively (Mihara 1997). They are directly involved in selenium metabolism (as well as in sulfur's), and they were proposed to be a possible selenium donor for SPS proteins (Mihara 2002). Bacterial NifS proteins exhibit sequence homology to metazoan protein selenocysteine lyase, which, nonetheless, appear to be specifically acting only on selenocysteine.
The fusion of NifS and SPS proteins was already observed in (Zhang 2008), uniquely in species *Geobacter sp. FRC-32*, a deltaproteobacteria classified among Desulfuromonales.

Our procedure recovered the known, fused protein in *Geobacter* species. Interestingly, the SPS domain contains a selenocysteine, in the usual site, a case unique among all SPS fusions detected: all others are with cysteine at this site. Notably, we identified an additional SPS protein in *Geobacter*, also selenocysteine containing, but without extensions.

Caldithrix abyssi is a bacterial species that seems to represent a novel lineage of its own (see Miroshnichenko 2003). In this species we found a *NifS-SPS* fusion, in which *SPS* is with cysteine. Additionally, we identified another *SPS* gene with selenocysteine in the same genome. This gene appears to be normal (not fused), although we couldn't find a starting Methionine. Several other selenoproteins, and Sec machinery proteins were identified in the genome, supporting the fact that this species utilizes selenocysteine.

But NifS-SPS proteins are not limited to prokaryotes, since we found also in the genomes of two protist eukaryotes: the heterolobosean *N.gruberi* (see above) and the amoeba *Acanthamoeba castellani*. Both genes include a number of (small) introns, and have a cysteine aligned to the Sec position of *SPS2* genes.

As previously said, we identified an additional *SPS* gene in the *N. gruberi* genome fused with a methyltransferase, and a few selenoproteins. In the genome of *A. castellani* we found 5 selenoproteins, similar to the *Naegleria* set: a thioredoxin reductase, two deiodinase-like proteins, a glutathione peroxidase, and selenoprotein O. Additionally a partial N-terminal SPS sequence was found in this organism, carrying an in-frame TGA in the expected place. Due to the limited availability of sequences from this organism, it is unclear whether this represents the only sequenced fragment of a real additional *SPS* gene in this organism, or if it is a relic of a gene that was lost, or even if this comes from a genomic contamination.

Considering that all species with a *NifS-SPS* fusion possess also another copy of *SPS* (possibly with the exception of *A.castellanii*), it is possible that the fused protein cannot fully perform the original *SPS* function. Speculating, we could expect that it can phosphorylate selenium only when NifS is the donor (when recycling selenocysteine).

NADH dehydrogenase-SPS fusions

The *SPS* fusion with domains similar to the NADH dehydrogenase (Ubiquinone) (complex I) was already observed in (Zhang 2008), and appeared to be very common within prokaryotes. Consistently we detected such fused genes in a wide range of bacteria, including Cyanobacteria (Prochlorales, Oscillatoriophycideae, Stigonematales), Alphaproteobacteria (Rhodobacterales, Rhodospirillales), Gammaproteobacteria (Alteromonadales, Oceanospirillales, Methylococcales, Chromatiales), Betaproteobacteria (Burkholderiales, Nitrosomonadales).

Interestingly, this fusion was detected also in several eukaryotic species, belonging to a number of diverse basal lineages (see Figure 2): *Ostreococcus tauri*, *Ostreococcus lucimarinus*, *Chlorella variabilis*, *Coccomyxa subellipsoidea* (all green algae), *Aureococcus anophagefferens* (pelagophyte), *Phaeodactylum tricornutum* (diatom), *Ectocarpus siliculosus* (brown algae), *Emiliania huxleyi* (haptophyte), *Toxoplasma gondii* (Apicomplexan), and even the metazoan *Hydra magnipapillata* (cnidaria - hydrozoan).

Figure SM2.1 shows the sequence-based predicted phylogeny of the identified NADH-dehydrogenase / SPS fusions, bacterial and eukaryotic altogether. Most of eukaryotic NADH-SPS proteins cluster together, with two exceptions: *Toxoplasma* and *Hydra* sequences are clustering within bacterial sequences, quite far one from another and also far from the other eukaryotic fusions. This supports the idea that NADH-SPS fusions emerged more than once during evolution. Actually, the *Hydra* fused protein resembles so much the bacterial homologues that it is entirely possible that this is artifactual, coming

from a bacterial genomic contamination. The lack of introns would support this. In *Toxoplasma* and most other eukaryotic species, the gene has introns so we can be confident that it is really integrated in the genome, and functional. Interestingly, the phylogenetic cluster of eukaryotic sequences contain two *Rhodospirillales* (Alphaproteobacteria) sequences (figure SM2.1). This may suggest that horizontal transfer occurred between these lineages.

Other gene extensions

Several other extensions of *SPS* genes were predicted in prokaryotes and protist eukaryotes. Typically these are found in single species. In general, due to the low number of available sequences, this makes their call much less reliable, and thus we do not report them here.

In *Plasmodium* species, we detected a 5' extension of a Cys-*SPS* which we believe to be very reliable, since we observe it conserved in all 7 investigated genomes in this lineage. This extension is about 500/550 amino acids long, and shows no homology with any known protein domain. *Plasmodiums* have a very lineage-specific selenoproteome (Lobanov 2006), with at least 4 conserved selenoproteins with no homology to any other known protein. The function of the extension remains totally unknown.

Figures in Supplementary Material S2:

Figure SM2.1: (next page)

Reconstructed protein phylogeny of all NADH dehydrogenase - SPS fused proteins identified in the tree of life. For full readability download this from big.crg.cat/SPS and visualize on screen.

Phylogeny of Selenophosphate synthetases (SPS)

(Figure SM2.1)

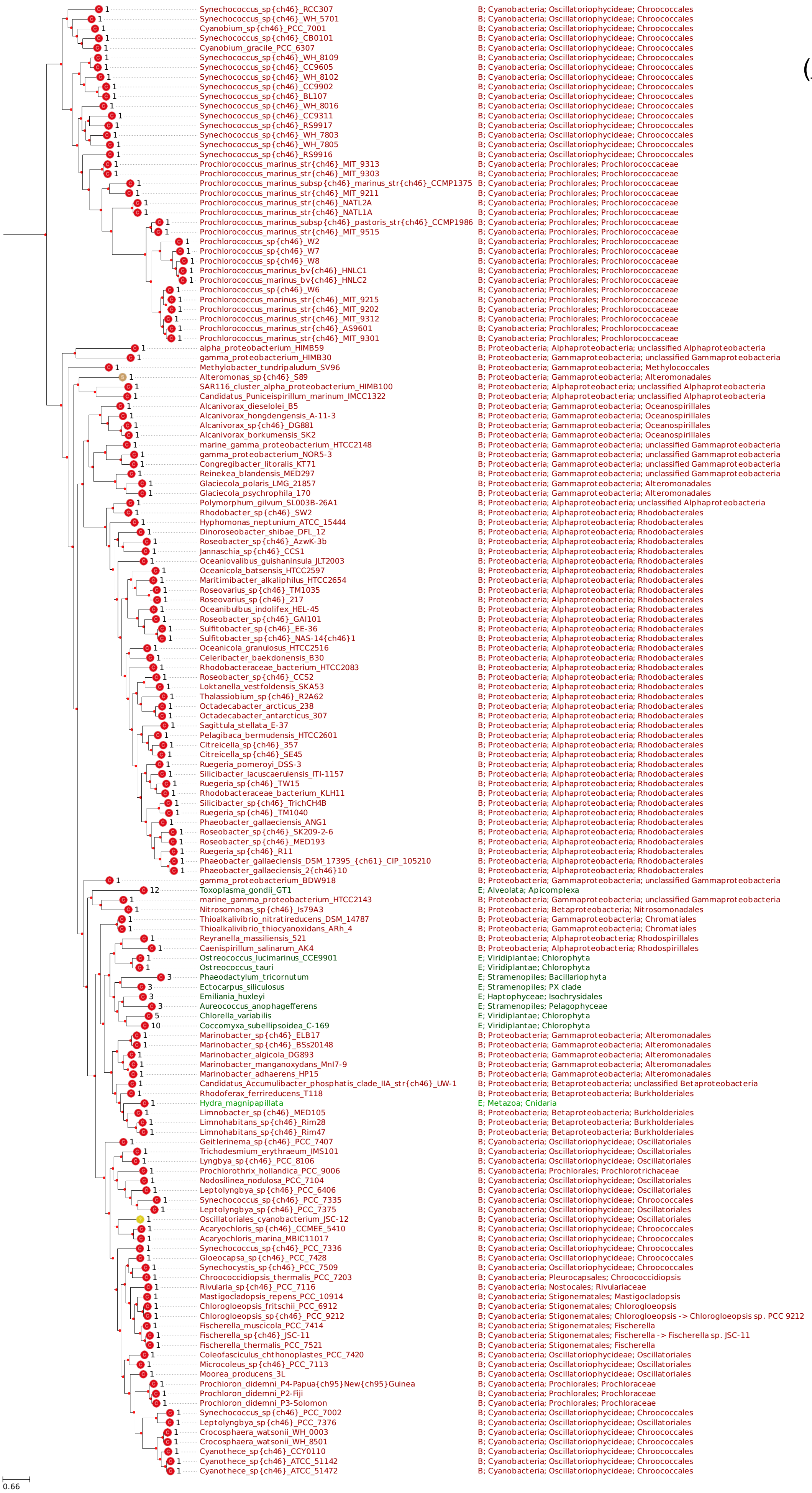

Supplement: Supplemental Material [file supp_gr.190538.115_Supplemental_Material_S2.pdf]
