# Supplementary material for: Evolution of selenophosphate synthetases: emergence and relocation of function through independent duplications and recurrent subfunctionalization
Source: Genome Res. 2015 Sep;25(9):1256–67. doi: 10.1101/gr.190538.115 (PMC4561486; doi:10.1101/gr.190538.115)

Supplementary Material S3:

Phylogeny of eukaryotic SPS proteins

We produced our *SPS* gene set by running Selenoprofiles ver. 3 on our collection of eukaryotic and prokaryotic genomes (see Methods). Then, we run our phylogenetic reconstruction procedure (see Methods) on each of these SPS protein sets: all predictions in eukaryotes; all predictions in prokaryotes; predictions in the prokaryotic reference set of species; and prokaryotic reference plus all eukaryotic predictions.
After inspecting results, we manually filtered out lots of eukaryotic predictions for a variety of reasons, producing a reference eukaryotic set. Some predictions were obvious bacterial contaminations, which we filtered out. Then, a number of duplicated predictions were removed, which are caused by the presence of the same stretch of DNA in two locations of the genome assembly (these are common just in certain species, presumably for a poor assembly strategy).
Lastly, pseudogenes were excluded; vertebrate genomes in particular were found very rich in *SPS1* retrotransposed copies, recognizable for their lack of introns, presence of in-frame stops and/or frameshifts, and lack of detectable transcription (no ESTs, no RNAseq reads).

Phylogeny of *SPS* across bacteria, archaea and eukarya

Figure SM3.1 shows a tree of prokaryotic (reference set) and eukaryotic SPS proteins pulled together (we suggest to download large images like this one from http://big.crg.cat/SPS for visualization on screen). The predicted tree of *SPS* genes follows approximately the phylogenetic relationship among the species to which the genes belong. Exceptions are found within certain basal eukaryotic lineages (protists), that possess a bacterial-like *SPS* when compared with metazoans. This includes all green algae (Chlorophyta), alveolates, but also some amoebozoa and heterolobosea (labelled as protist #1 in figure SM3.1 and SM3.2). From the phylogenetic signal, it appears that at least three distinct horizontal transfer events occurred (see figure SM3.1). All fused SPS proteins identified in eukaryotes (see Supplementary Material S2) are found in this part of the tree, clustering with bacterial sequences (note that the non-SPS region were not included in the alignment used to reconstruct phylogeny). This suggests that the eukaryotic fused *SPS* genes were horizontally transferred from bacteria. This is valid also for the only candidate *SPS* fusion in a metazoan, the *NADH/SPS* gene identified in cnidarian *Hydra magnipapillata.* Nonetheless, we suspect that this gene actually comes from a bacterial contamination (see also Supplementary Material S2).
Excluding *Hydra*, the concordance in the metazoan cluster in the tree extends considerably (see figure SM3.1). Placed as outgroup of metazoan sequences, we find several protists including choanoflagellates, close relative of metazoans. Then, archaeal *SPS* is placed nicely as outgroup of this eukaryotic cluster. These results strongly support the continuity of the *SPS* gene across the domains of life, with the last common ancestor of prokaryotes and eukaryotes possessing *SPS* and, very likely, other selenoproteins.
The continuity is apparently broken only for the protists lineages mentioned above, that possess a bacterial-like *SPS* (sometimes with gene fusions). Considering that the rest of Sec machinery in these lineages does instead show continuity (data not shown here), the

most likely explanation for this pattern is that, at some point, these organisms replaced their own ancestral *SPS* gene with a bacterial (fused) *SelD* gene, acquired by horizontal gene transfer.

SPS forms with no Sec nor Cys: *SPS1*

Figure SM3.2 shows the predicted protein phylogeny of the eukaryotic reference set.
Mostly, non-metazoan eukaryotes possess a single *SPS* gene with either selenocysteine or cysteine, like bacteria. Other forms, with something different from these two amino acids at that position, are found only in metazoans (see also Figure 2). We refer to these genes as *SPS1*. Specifically, we detected *SPS1* genes in jawed vertebrates, Clitellata annelids, insects, and certain tunicates. Through phylogenetic reconstruction (explained below), we concluded that *SPS1* genes were generated independently in these lineages by duplication of *SPS2*. The alternative, simplest explanation would be that SPS1 genes derived from a common *SPS1* ancestor already present at the root of metazoans. Yet, there are several evidences pointing to the independent duplication scenario instead. First, there is very strong phylogenetic signal to support that the human *SPS1* gene was generated at the root of vertebrates by duplication of *SPS2* (Figure SM3.2). Second, the analysis of species tree shows that if *SPS1* genes had a single common origin, then a high number of gene loss events would be implied to explain the current pattern of gene presence, since many metazoan lineages possess only *SPS2* (detailed later).
However, the result of the phylogenetic tree reconstruction does not fully match the expectations of independent duplications that we propose. In particular, insect *SPS2* sequences (as well as the annelid *Helobdella robusta SPS2* sequence) are placed closer to the root (i.e. more basal) than expected from the gene history that we predict (Figure SM3.2). We believe that this is cause by the change in divergence rate of the insect SPS2 gene after duplication (analyzed in detail in Supplementary Material S5). This effect is known to confound phylogenetic reconstruction methods by causing long branch attraction.
In the following paragraphs, we describe first in detail our analysis of vertebrate and Clitellata *SPS* genes. Then, we investigate the likelihood of the two duplication scenarios according to statistical test analyses. Finally, we proceed to explain the complex evolution of *SPS* in insects, which is complicated by many factors. Supplementary Material S4 is dedicated to the analysis on tunicate *SPS* genes. In this lineage, we observed a clear intermediate state prior to gene duplication: a single *SPS* gene producing two transcript isoforms, one SPS1-like and one SPS2-like.

SPS1-Thr in vertebrates

The great majority of vertebrates were predicted to possess exactly two SPS genes, one with selenocysteine (*SPS2*) and one with threonine (*SPS1-Thr*). Among the few exceptions, non-placental mammals (such as marsupials) possess two copies of *SPS2*, one of which is intronless. As described in (Mariotti 2012), at the root of placentals *SPS2* was functionally replaced by one of its retrotranscribed copies. Non-placental mammals still retain both copies, although it is unclear whether they are both functional.
In the bird genome assemblies (genus *Melopsittacus, Taeniopygia, Gallus, Meleagris*), only *SPS1* was found. Nonetheless, we could identify *SPS2* in some EST sequences from *Gallus gallus*, which can not be mapped back to the genome. Thus we believe birds actually possess both *SPS1* and *SPS2* in their genome, but the latter is missing from the assemblies, presumably because of characteristics of their genomic location that make sequencing difficult.

We did not find *SPS1* sequences in any jawless vertebrates (e.g. lampreys), despite recent availability of a genome and abundant ESTs.
Thus, with the only exception of non-placental mammals, we predict that all the rest of jawed vertebrates (Gnathostomata) possess the two genes *SPS1-Thr* and *SPS2*, and we ascribe their absence in few species in our prediction set to the imperfect quality of genomes.
Non-vertebrate deuterostomes (such as *Strongylocentrotus* and *Branchiostoma*) possess a single gene with selenocysteine (*SPS2*). Along with the conservation of intron positions between the two genes (see figure SM3.2), and with the strong phylogenetic signal, this supports the fact that the vertebrate SPS1-Thr gene was generated by duplication of *SPS2* approximately at the root of gnathostomes.

SPS1-Leu in Clitellata (Annelida)

In the genome of Annelida species *Helobdella robusta*, we identified two *SPS* genes, one with selenocysteine (*SPS2*) and another one carrying leucine aligned to the Sec position (*SPS1-Leu*). The only other annelidan genome in our datasets (*Capitella teleta*) appears to possess a single *SPS2* gene instead. Thus, we downloaded all EST data available at NCBI from the lineage of Annelida, and we scanned them with Selenoprofiles to detect *SPS* genes. We found two distinct situations in the two main annelidan lineages, Polychaeta and Clitellata.
In the lineage Polychaeta, we have sublineages Sipuncula (represented by ESTs of species *Sipunculus nudus*) and Scolecida (represented by the genome sequence of *Capitella teleta* and by ESTs of *Capitella teleta*, *Malacoceros fuliginosus* and *Alvinella pompejana*). In all these cases, we found a single Sec-containing *SPS* gene (*SPS2*).
The lineage Clitellata is represented in our datasets by the genome sequence of *Helobdella robusta*, and by the ESTs of *Helobdella robusta*, *Hirudo medicinalis* and *Tubifex tubifex*. In all these species, we found both *SPS2* and *SPS1-Leu*. In the genome of *H. robusta*, we can see that these two genes possess a nearly identical intron structure. Both genes have EST data support in *H. robusta* and *H. medicinalis*, with *SPS1-Leu* much more abundantly transcribed. In *T. tubifex* (for which we have relatively few EST reads, and no genome) we could not observe *SPS2*, although we think that this is due only to low sequence coverage. Notably, we observed two very similar *SPS1-Leu* proteins in the EST data of this species.

In figure SM3.3, we compiled a collection of all *SPS* sequences found in Annelida ESTs and genome sequences. Altogether, data indicates that the ancestral annelidan SPS2 gene duplicated in Clitellata, generating the *SPS1-Leu* gene, that conserved the intron structure of its parental gene. Then, this new gene may have duplicated again in the lineage of *Tubifex tubifex*, after the split of Oligochaeta (containing *T.tubifex*) with Hirudinida (containing *H.robusta* and *H.medicinalis*).

Testing duplication topologies: ancestral or lineage-specific?

The precise topology of *SPS* gene duplication and losses proved to be hard to resolve, particularly in insects. This is due mostly to the high rate of sequence evolution of *SPS2* in dipteran insects (see also Supplementary Material S5). As you can see in figure SM3.2, the phylogenetic reconstruction procedure places the dipteran SPS2 proteins basal to both vertebrate SPS1 and SPS2. The literal interpretation of this tree is that an ancestral duplication occurred, with subsequent gene losses. We think this is just an effect of high sequence diversity in Diptera, a phylogenetic artifact known as long branch attraction.

In fact, if the *SPS* duplication originating the extant insect *SPS* genes was truly ancestral to metazoans, it would imply that one of the resulting genes was lost in each of the following lineages independently: Cyclostomata, Oikopleura (non-ascidian tunicate), Echinodermata, Enteropneusta, Branchiostoma, Crustacea, Myriapoda, Arachnida, Nematoda, Polychaeta (annelid), Platyhelminthes.

Considering this, we believe that the ancestral duplication scenario is very unlikely, and that our observations are better explained by independent duplication events in the different lineages (insects, jawed vertebrates, clitellata, ascidians). However, phylogenetic reconstruction methods consistently report the ancestral duplication topology as more likely.

We attempted to quantify how different the two scenarios were in terms of computed likelihood. Thus, we built two "artificial" phylogenetic trees, representing the two possible duplication topologies for insects and vertebrates (see figure SM3.4). The ancestral duplication topology is the output of our phylogenetic reconstruction method. The independent duplication topology is a modification of that tree, in which we moved the insect *SPS2* branch together with insect *SPS1*.

On each tree, we ran the branch length optimization by phyml (Guindon 2010), and we computed the likelihood of the resulting trees. We then used the "Approximately Unbiased" test implemented in the program CONSEL (Shimodaira and Hasegawa 2001) to compare the likelihood of the trees corresponding to the two scenarios. This test resulted in *p*-value of 0.125, implying that the lowest scoring topology cannot be discarded at 5% significance level.

In simple words, this test shows that the independent duplication scenario is predicted to be, based on observations on sequences alone, almost as likely as the ancestral duplication scenario. Considering the genes losses that would be implied by the ancestral duplication scenario (see above) and considering also the high rate of divergence in certain *SPS2* genes (Supplementary Material S5), a major confounding factor for phylogenetic reconstruction, we must conclude that the independent duplication scenario is the correct one. We then worked to solve in detail the *SPS* phylogeny in insects, complicated by the occurrence of *SPS2* gene losses concomitant to selenocysteine extinctions in many lineages.

SPS phylogeny in arthropods and insects

We predicted the last common ancestor of all arthropoda to possess a single *SPS2* gene (with Sec). To resolve the gene phylogeny within arthropoda, we created an alignment of all *SPS* genes found in arthropoda, and we run our phylogenetic reconstruction pipeline. The resulting tree can be inspected in figure SM3.5. Non-insect arthropods appear to possess only a single *SPS2* gene. *Ixodes scapularis* only has a second copy of this gene, also with TGA. The protein tree suggests that this is a species-specific duplication. As the genome assembly available is quite fragmented, we cannot know whether the two genes possess a SECIS element, but we expect at least one gene to have one.

Among insects, different gene sets were identified in different lineages, including other *SPS1* proteins.

In all Diptera, Lepidoptera and Coleoptera we identified an *SPS1-Arg* gene, and these genes clearly cluster together by sequence similarity. Then, in Diptera we also observed the Sec-containing *SPS2* genes, which also cluster together, although with a higher degree of diversification. In *Drosophila willistoni* alone - the only known drosophila that lost selenoproteins - *SPS2* was not found, consistent with its function being related to selenocysteine synthesis. Analogously, the selenoprotein-less orders of Lepidoptera and Coleoptera (Chapple 2008) lack *SPS2*.

Hymenoptera were found to possess a single UGA containing *SPS* gene (*SPS1-UGA*). No convincing SECIS could be identified downstream in any hymenopteran genome, and we believe *SPS1-UGA* to be readthrough with another mechanism.
Additional gene fragments, similar to *SPS1*, were found in some hymenopteran genomes, and also in the fly genomes of *D.persimilis* and *D.pseudoobscura* (see figure SM3.5). However none of those were supported by EST data (in contrast with the readthrough gene, abundantly confirmed). Although we cannot rule out that these genes are true SPS family expansions in these lineages, it is most likely they are just non-functional retrotransposed copies, and thus we excluded them of all subsequent analysis.
Lastly, we found a very interesting situation in the basal insect group of Paraneoptera, with 3 genomes available: *Pediculus humanus* (Phthiraptera)*, Rhodnius prolixus* (Hemiptera)*, Acyrthosiphon pisum* (Hemiptera). We identified selenoproteins and complete machinery in the genomes of both *P.humanus* and *R.prolixus*. Also, we found two UGA containing *SPS* genes in these species. One had a clear SECIS, and clustered roughly with Dipteran *SPS2*. The other had no SECIS, and clustered with known insect *SPS1* genes and hymenopteran *SPS*, and with arthropod *SPS2* as outgroup (see figure SM3.5). These two genes in *R.prolixus* share a very similar intron structure, while the *SPS2* gene in *P.humanus* has no introns at all. In contrast *A.pisum,* that lost selenoproteins (Aphid Consortium 2010), contained a single *SPS* gene with arginine, also clustering with other *SPS1* genes.

All together, we think that data supports the following phylogenetic history (you may follow Figure 3 in the main paper). At the root of insects, the *SPS2* gene was duplicated conserving its intron structure. The ancestral copy retained the SECIS element, and presumably kept the SeP production function (*SPS2*). This gene started to evolve faster just after the duplication, since we see it highly divergent in all insects.
The other copy did not retain the SECIS, and we believe that it exerts its function through a Sec-independent readthrough. This gene can be seen in this state in extant hymenopterans, as well as in paraneopteran *R.prolixus* and *P.humanus.* Then, both at the root of Diptera/Lepidoptera/Coleoptera, and in the lineage of *A.pisum* (after the split with the other paraneoptera in our set), this gene mutated the UGA codon to an arginine codon, becoming what we know as (drosophila) *SPS1*. Thus, we refer to all the progeny of this SECIS-lacking, UGA-containing *SPS* as (insect) *SPS1*, using the suffix UGA to denote the genes in which the UGA is still present and readthrough (e.g. *SPS1-UGA* Hymenoptera).
As we discovered later, this phylogenetic history is also well supported by analysis of the secondary structures and motifs found near the UGA site (see Supplementary Material S6).

Figures in Supplementary Material S3:

Figure SM3.1: (next page)
Reconstructed protein phylogeny of SelD/SPS proteins in the prokaryotic and eukaryotic reference set. The full size image can be downloaded from http://big.crg.cat/SPS, and visualized on screen at the desired level of zoom. On the left, the tree shows the predicted phylogenetic topology, with speciation and duplication events displayed as red and blue branch points, respectively. Colored balls are used to indicate the type of SPS (residue found at Sec position), as illustrated in Figure 4 of main paper. In addition to the protein types in Figure 4, here there are also some predictions in which the residue in Sec position is unknown, because not aligned; those are indicated as grey balls containing "-". A few predictions contain pseudogene features (frameshifts or stop codons), and are indicated as dark grey balls containing "Ψ". Next to each colored ball, the numeric id assigned by Selenoprofiles is reported, allowing to identify uniquely this gene in the sequence set in Supplementary Material S8. Then, two columns report the species to which the gene belongs to, and a summary of their ncbi taxonomy. Both species and taxonomy are colored according to their kingdom: bacteria are in red, archaea are in blue, and eukaryotes in green (with darker green for non-metazoan eukaryotes). On the right, large colored labels were added to help the visual identification of orthologous groups even without zoom in.
One can notice that, from archaea to the bottom, the predicted protein tree is mostly in concordance with the tree of investigated species, supporting a scenario of continuity. The continuity is not respected only in the upper part of the tree, with some protists (labelled protist #1) possessing a bacterial-like *SPS*. All eukaryotic fusions are found in this cluster.

Figure SM3.2: (2 pages ahead)
Reconstructed protein phylogeny of the eukaryotic reference set of SPS proteins. The full size image can be downloaded from http://big.crg.cat/SPS, and visualized on screen at the desired level of zoom. See caption of figure SM3.1 for plot explanation. In respect to SM3.1, an additional column is present, displaying each gene as a colored rectangle. The width and position of the rectangle represents how the prediction spans the protein profile; black lines are used to indicate the intron positions, as projected in the protein alignment.

Phylogeny of Selenophosphate synthetases (SPS)

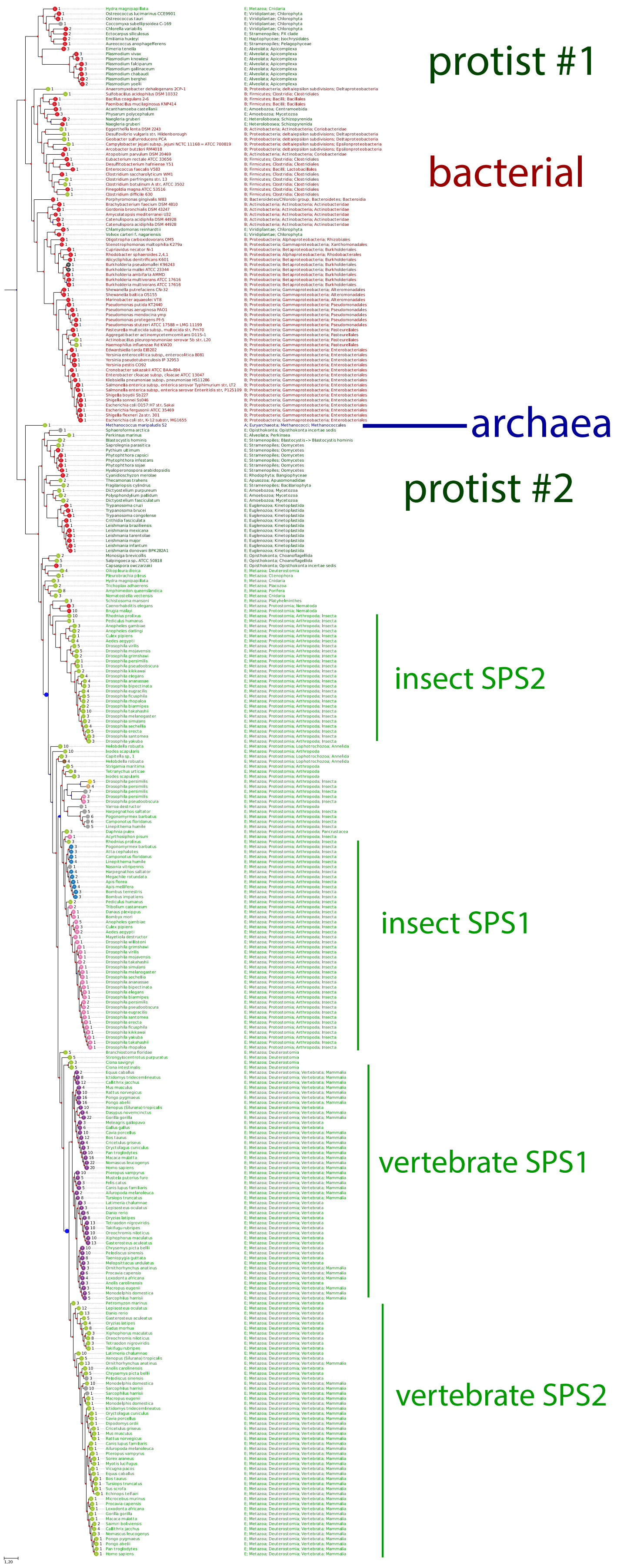

Phylogeny of Selenophosphate synthetases (SPS)

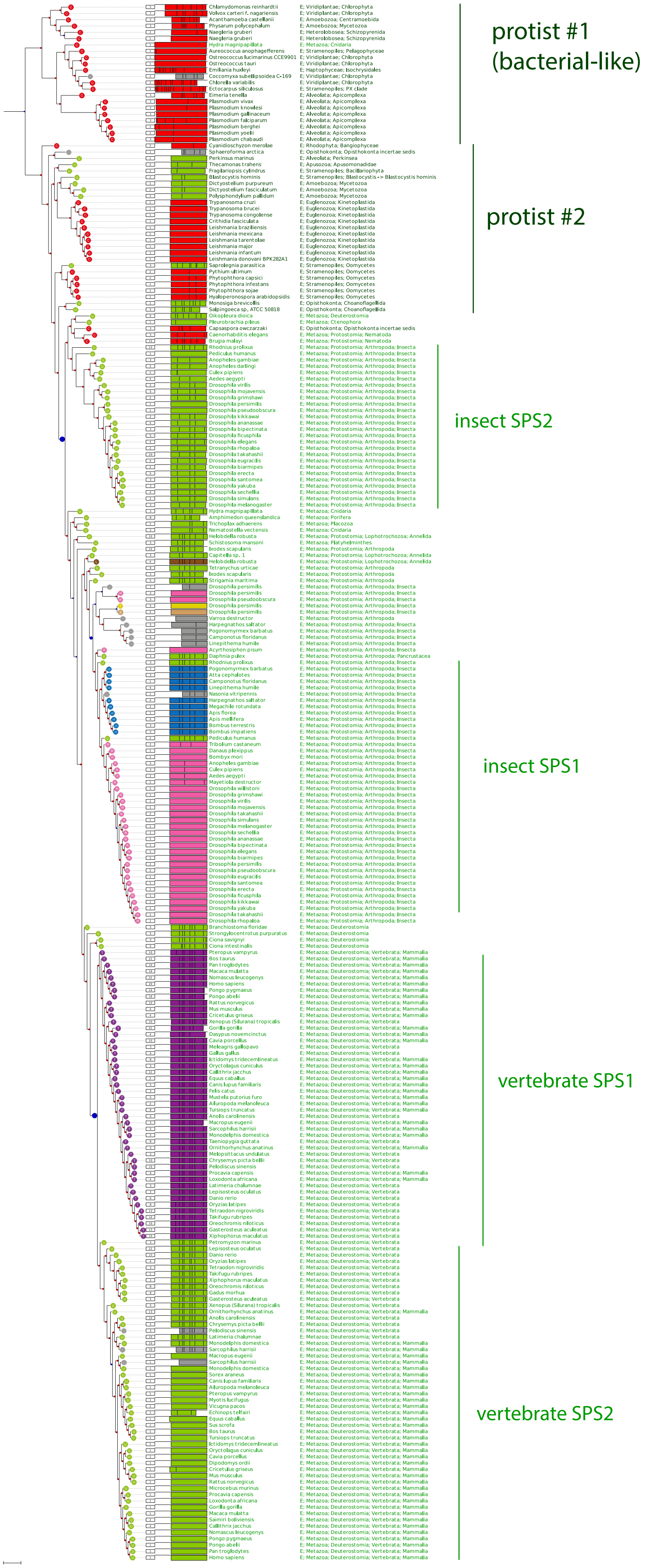

Figure SM3.3:
Alignment of SPS forms found in genomes and EST of species of Annelida. The Sec position is highlighted in purple.

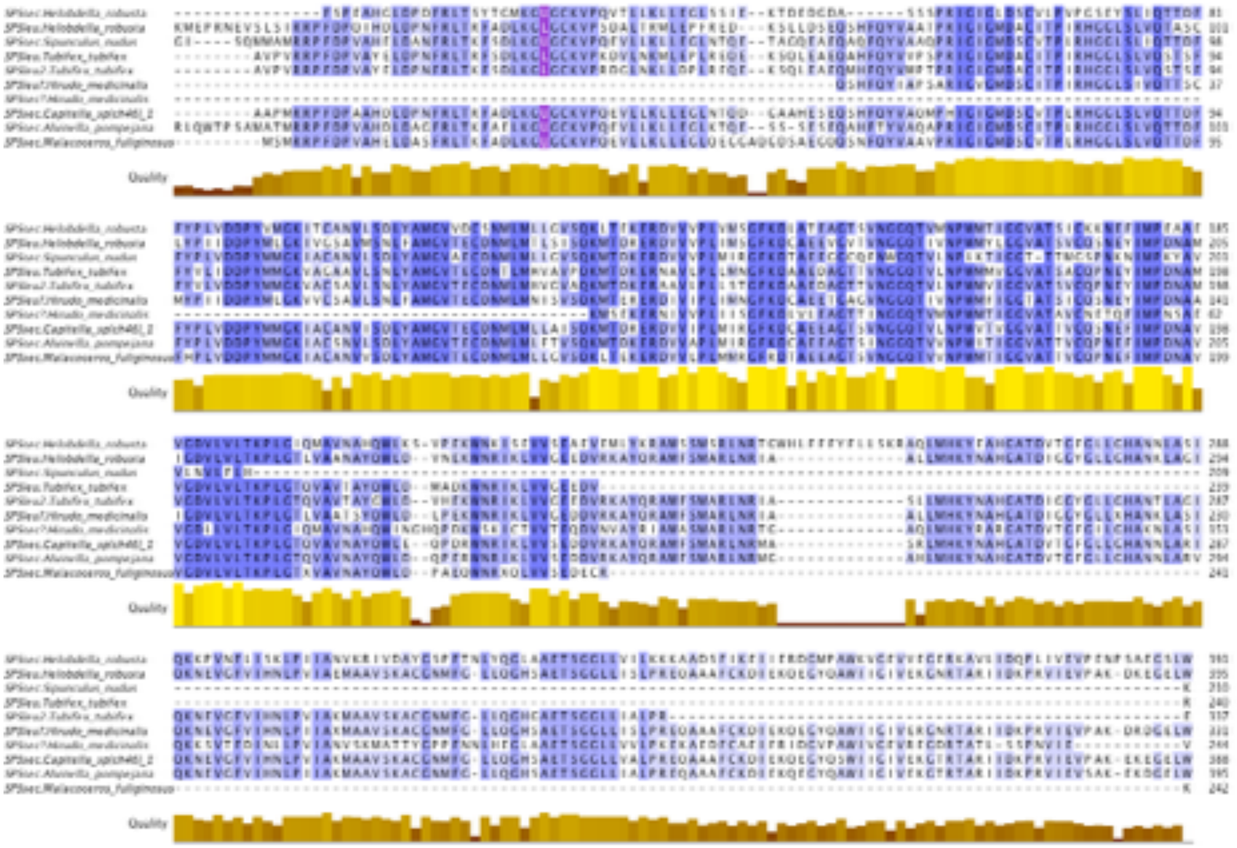

Figure SM3.4:
The two “backbone” phylogenetic trees representing the two possible topologies for vertebrate and insect *SPS* duplications: ancestral duplication (a) or independent duplications (b). Speciation nodes are colored in red, while duplication nodes are in blue. The ancestral duplication (the result of ML reconstructions) has better score. Nonetheless, its score is not statistically different than the one for the independent duplications, which is largely better supported by other observations.

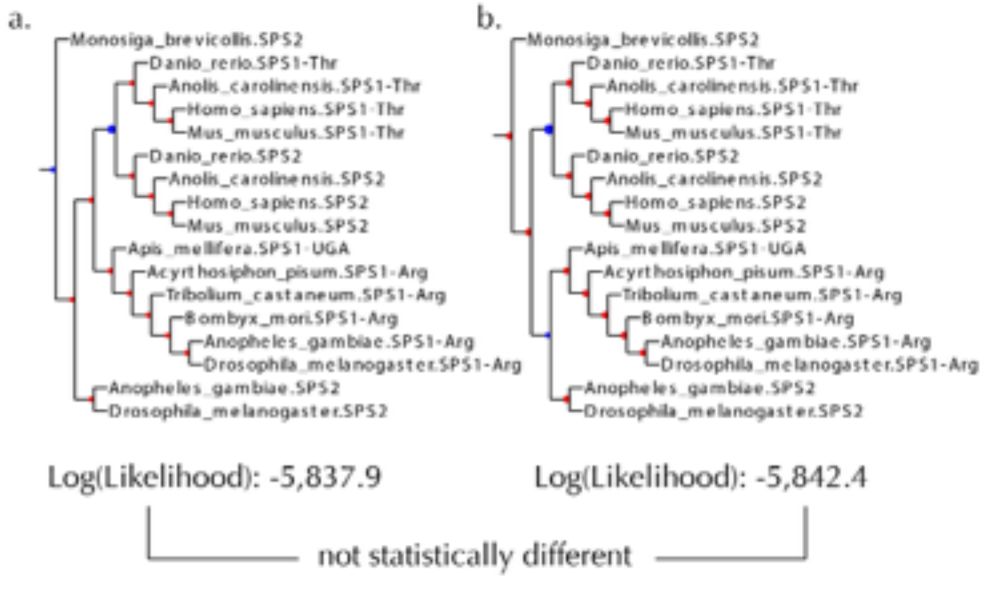

Figure SM3.5:

Reconstructed protein phylogeny of arthropoda *SPS* genes. See caption of figure SM3.1 and SM3.2 for plot explanation. UGA containing genes were classified as selenocysteine coding (green) or UGA (blue) based on phylogenetic clustering and presence of SECIS. The full size image can be downloaded from http://big.crg.cat/SPS, and visualized on screen at the desired level of zoom.

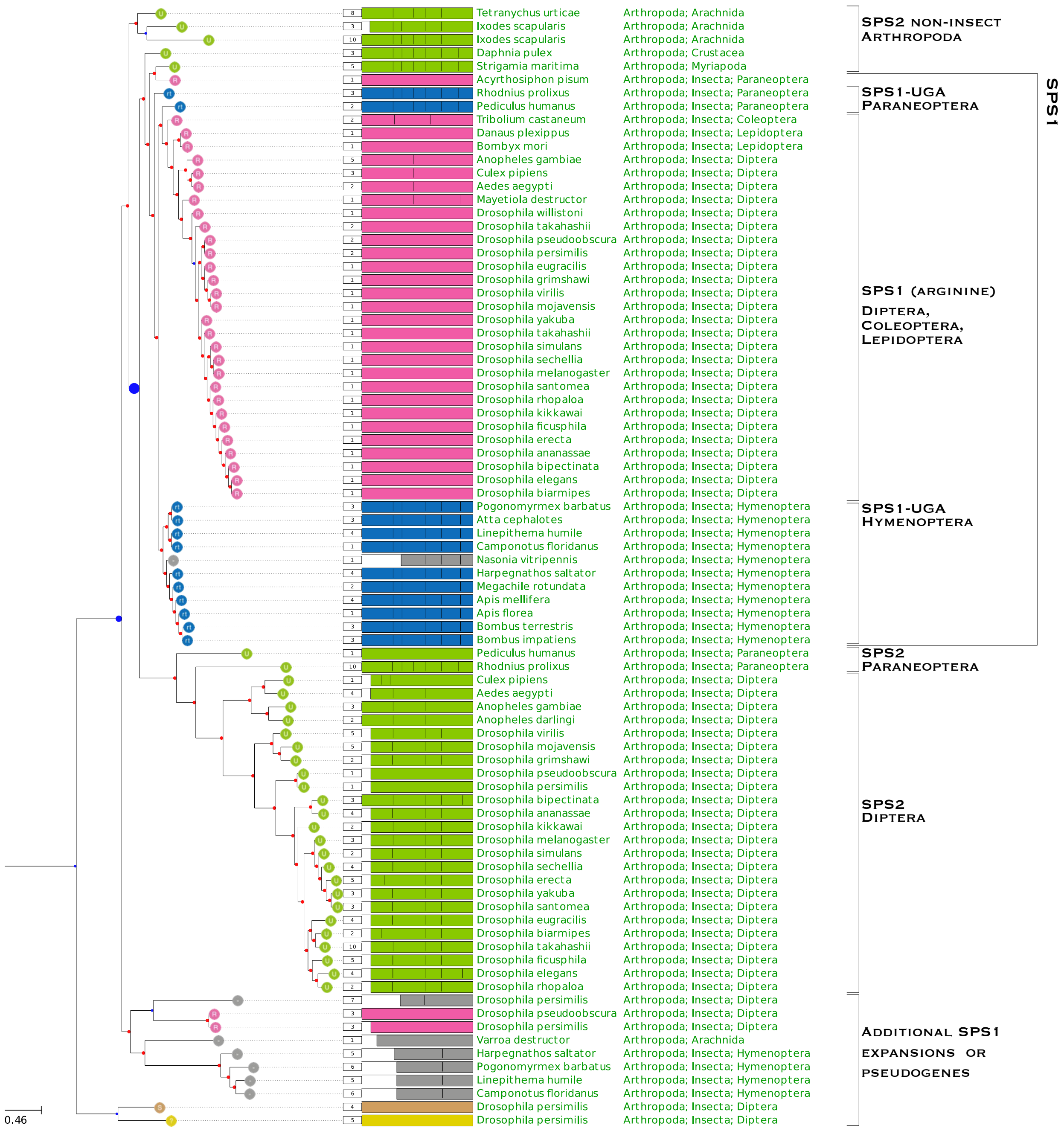

Supplement: Supplemental Material [file supp_gr.190538.115_Supplemental_Material_S3.pdf]
