# Supplementary material for: Evolution of selenophosphate synthetases: emergence and relocation of function through independent duplications and recurrent subfunctionalization
Source: Genome Res. 2015 Sep;25(9):1256–67. doi: 10.1101/gr.190538.115 (PMC4561486; doi:10.1101/gr.190538.115)

Supplementary Material S4:

Alternative isoforms sorted by gene duplication in ascidians

In our annotation of metazoan *SPS* genes we ran into an interesting case in tunicates, that constitute the most closely related outgroup to vertebrates (Delsuc 2006).
In the genome of *Ciona intestinalis*, we initially identified a single *SPS* gene, with glycine (Gly) aligned to Sec position. Later, we found that this gene actually produces two alternative forms, one with selenocysteine and one with glycine. The two forms differ only by the two first exons, and they are both supported by EST data (see figure SM4.1). Both include the same SECIS element in the 3'UTR.
Both alternative forms were found conserved in *Ciona savignyi*. In this genome assembly though, we see a single base insertion in the coding sequence, near the N-terminal, which, if real, would produce a frameshift. Nonetheless, we believe this to be artifactual, given the conservation in the rest of the region. Here we show the N-term portion of the alignment between *SPS-Gly* form of *C.intestinalis* (Query) and the corresponding genomic region in *C.savignyi* (Target); the glycine aligned to the Sec site is displayed in red.

```
Query   MALRPKFDPQSHNLDKKFRLTKYTG <---Intron---> LHGGGCKVPN ! FRAME ! DVLVKLLQELGANPYHDEQYMGGMIMPRLG <---Int
        |||||||||//| |/|||||||||| <    58nt    > |||||||||| ! SHIFT ! ||||||| |||/| ||||||/||||||||| <    93
Target  MALRPKFDPETHGLNKKFRLTKYTG                 LHGGGCKVPN    1nt    DVLVKLLHELGSNQYHDEQYIGGMIMPRLG
        agcacatgcgacgcaaatccaatag                 ccgggtagca           ggtgatccgcgtactcggctaggaaacctg
        tctgcatacacagtaaatgtcaac     gt     ag   gtaggggatca     g     atttattaatgcaaaaaaaatggtttcgt   gt
        gtagagttaaattgcaatagagtt                 gacatttagct           tcgtagctattatgtctagtcccgagaca

Query   ron---> IGLDCCVIPLRFGGLSLLQTTDFFYPLIDDPYMM <---Intron---> GKIACANVLSDLYAIGVTECDNMLMLLGVSSKFTEKERDTVI
        nt    > |||||||||||||||||||||||||||||||||| <    71nt    > ||||||||||||||/|||||||||||||||||||||||||||
Target          IGLDCCVIPLRFGGLSLLQTTDFFYPLIDDPYMM                GKIACANVLSDLYAVGVTECDNMLMLLGVSSKFTEKERDTVI ...
                agcgttgaccctggctcccaagtttccaggctaa                gaagtgagtagttggggagtgaacaccggatatagagagaga
          ag   gtgtaggttctgtggtcttaccattacttaacatt   gt     ag   gatcgcattgatactgtcagaatttttgtgcatcaaagactt
               cccgtctcattgtaccactgattctttattcgtgg                tatttcttgttgtatgaaacctgtgttcctcaccaaaaccct
```

Below, the N-term portion of the alignment between the other form (*SPSsec*) of *C.intestinalis* (Query) and the same genomic region in *C.savignyi* (Target) is shown. Notice that the two last exons shown (as well as the rest of the gene structure downstream, omitted here) are identical in the alignments above and below.

```
Query   KWDPVVHELSEEFRLTNFTGLKGUGCKVPQKVLLKLLEGLEALS--NGFQNGQLQPTPTVG <---Intron---> IGLDCCVIPLRFGGLSLLQTTDF
        ||||| | | ||||||||||||||||||||||||||||| |//|  ||| |  /||||||| <   520nt    > |||||||||||||||||||||||
Target  KWDPVFHGLQEEFRLTNFTGLKGUGCKVPQKVLLKLLEGFESVSNGNGFHNNNMQPTPTVG                IGLDCCVIPLRFGGLSLLQTTDF
        atgcgtcgccggtataatagtagtgtagccagccaccggtgtgtagagtcaaaaccacagg                agcgttgaccctggctcccaagt
        agacttagtaaatgtcatcgtaggggatcaatttattagtactcagagtaaaatacccct   gt     ag   gtgtaggttctgtggtcttaccat
        ggcgtctgcaggcgaaccttaagagcgccggtgcgtggatattctatgcctccgaaccat                cccgtctcattgtaccactgattc
                               *

Query   FYPLIDDPYMM <---Intron---> GKIACANVLSDLYAIGVTECDNMLMLLGVSSKFTEKERDTVI
        ||||||||||| <    71nt    > ||||||||||||||/|||||||||||||||||||||||||||
Target  FYPLIDDPYMM                GKIACANVLSDLYAVGVTECDNMLMLLGVSSKFTEKERDTVI    ...
        ttccaggctaa                gaagtgagtagttggggagtgaacaccggatatagagagaga
        tacttaacatt   gt     ag    gatcgcattgatactgtcagaatttttgtgcatcaaagactt
        tttattcgtgg                tatttcttgttgtatgaaacctgtgttcctcaccaaaaccct
```

To gain some phylogenetic context, we searched *SPS* genes also in all other sequenced tunicates species. Figure SM4.2 shows an alignment of results found in all non-*Ciona* tunicate ESTs. In *Oikopleura dioica*'s genome and ESTs, a single *SPS* gene was identified, with only a Sec form produced. In *Molgula tectiformis* EST data, the two forms with Sec and Gly were found, with a prevalence of Gly forms. The two forms differed only at the 5' end, as in *Ciona*. In *Halocynthia roretzi* and *Botryllus schlosseri* EST data, we found two distinct forms in each species. In these cases though, we see that the two forms differed in several positions, with divergence not limited to the N-terminal region. The differences spread across all protein length, implying that two forms are produced by two distinct genes in these species. This was confirmed by the analysis of a preliminar genome assembly of *B.schlosseri*, not yet public at the time of our search, thus not included in our dataset (Ayelet Voskoboynik, personal communication). The two *SPS* genes as predicted from EST were both found in the genome assembly, in different scaffolds. Interestingly, while *B.schlosseri SPS-Gly* was found to possess approximately the same intron structure as the *Ciona* gene, *B.schlosseri SPS-Sec* gene has no introns. A SECIS element was found downstream of *Botryllus SPS-Sec*, but not downstream of *SPS-Gly*.
Showing here the alignment between the *SPS-Gly* isoform of *C.intestinalis* (Query) and *B.schlosseri SPS-Gly* gene (Target):

```
Query   DKKFRLTKYTGLHGGGCKVPNDVLVKLLQELGANPYHDEQYMGGMIMPRLG <---Intron---> IGLDCCVIPLRFGGLSLLQTTDFFYPLIDDPYM
        / | /    /|||||||||| /|| |||// | / | /| /|||||||||| <   624bp    > ||/| ||||||||||||||||||||||/|||||
Target  NNKSKSYTISGLHGGGCKVPREVLQKLLEDFGQSQYKEEHFMGGMIMPRLG                IGMDTCVIPLRFGGLSLLQTTDFFYPLVDDPYM
        aaatattaatgccgggtagcaggccatcggtgcactaggctaggaaacccg                agagatgactctggctttcaagtttctgggcta
        aaacacactcgtaggggatcgattaattaatgagaaaaaattggtttcgt   gt      ag   gtgtacgttctgtggtcttaccattacttaacat
        ctatagtttatttattcggcaacagaaggtccgcacgaatcgcggcgaaa                ctcgcgcgaagatgcttggggtttctcaccttttg

Query   M <---Intron---> GKIACANVLSDLYAIGVTECDNMLMLLGVSSKFTEKERDTVIPLMIHGFK <---Intron---> DSAEEAGTSINGGQTVL
        | <    54bp    > |||||||||||/||/|| |||||||||||||| /| ||| |/|||| ||| <   143bp    > | |/ ||||| ||||||
Target  M                GKIACANVLSDMYAMGVIECDNMLMLLGVSSKLSETERDVVVPLMIRGFK                DCADVAGTSITGGQTVL
        a                gaagtgagtagatgaggagtgaacatcggatactgagcggggctaacgta                gtgggggataaggcagc
        t   gt      ag   gatcgcattgatactgttagaattttgtgcatcacagatttctttggta   gt      ag   agcatcgcctcggactt
        g                aatccctggctgcggaacgtccggggttctgaataaagttattggcttcg                ctctggtagcacagtgg

Query   NPWCLIGGVATTVCQQNEFIM <---Intron---> PDNAVPGDVLVLTKPLGTQVACNSHQWLEQRNDKWNRIKLVVSEDEVEKAYHDAMFNMARLNR
        ||||||||| |/||| ||||| <   645bp    > ||||||||||||||||||||||| /||||/|  ||||||||///|/| ||/|||||| |/||||
Target  NPWCLIGGVGTSVCQPNEFIM                PDNAVPGDVLVLTKPLGTQVACVAHQWLDQAADKWNRIKLIINEEECEKSYHDAMFIMSRLNR
        actttaggggatgtccagtaa                cgaggcgggcgtaaccgacggtggcctcgcgggatacaacaaagggtgattcggataatacaa
        acggttggtgcctgacaattt   gt      ag    caactcgattttcactgcatcgtcaagtaaccaagagtatttaaaagaacaaacttttcgtag
        tggcgcctccggacgacacc                ggccttcacctcagggtggaatccgtagctgcccagttcgccccgaacaggttccgtcgaaccg

Query   TA <---Intron---> AQLMHTFNSHGATDVTGFGILGHAANLAKQQRSEVNFVIHNLPCIAKMAAIAKACGNMFGLLQGTSAETSG <---Intron
        || <   181bp    > ||||| / ||||||/||||||||| || ||||/||||/||||||| ||||/||||| ||||||||||||| <   105bp
Target  TA                AQLMHRYGSHGATDITGFGILGHAQNLVKQQRNEVNFIIHNLPCINKMAAVAKACGAMFGLLQGTSAETSG
        ag                gccacctgacggagaagtgatgcgcatgaccaaggataacacctaaaaggggagtggatgctcgatggatg
        c   gt      ag   ccattagaggagccatcgtgttgacaattaaagaatatttaatcgtaatcctcacggcttgttagcccacc    gt      a
        a                gcacgtgtcccctgccgctacgcctgcgggggcacccctctgccctggccctgtctcgcgggactgcata

Query   ---> GLLICLPREQAAKFCAEIKKVEGNQAWIIGIVEAG <---Intron---> NRTARIIEKPRVIEVQTLEDGTVQSVPPTSTPRRT
           > |||||/ ||/|||/| ||||//|/||||||||| | <   386bp    > /||||||/|||/|||/||/||||||/|| /| ||/
Target       GLLICMQREEAAKYCTEIKKIDGHQAWIIGIVEKG                DRTARIIDKPRIIEVETLDDGTVQSIPPVTTTRRS
             gccatacaggggattagaaaaggccgtaagaggag                gaagaaagaccaagggacgggagcaaccgaaaaca
        g   ggtttgtagaaccaagcataatagaacgttgttaa   gt      ag   gagccgttaacgttatactaagctagtcctcccggg
            catctcgggaaggatctatggtctcatgcccttga               ccggtatccgttctacatcccccgactgttgggaac
```

Below, the alignment between *SPS-Sec* form of *C.intestinalis* (Query) and *B.schlosseri SPS-Sec* gene (Target):

```
Query   WDPVVHELSEEFRLTNFTGLKGUGCKVPQKVLLKLLEGLEALSNGFQN-GQLQPTPTVGIGLDCCVIPLRFGGLSLLQTTDFFYPLIDDPYMMGKIACAN
        |||   | | //||||//|||||||||||||||||||||||       |   |   |   ||/||||| ||||||||||||||||||||||//||| ||||||||
Target  WDPEEHGLEKKFRLTDYTGLKGUGCKVPQKVLLKLLEGLTNDGQPSQREGFPQTLPTIGIGLDSCVIPLRFGGLSLLQTTDFFYPLVNDPYAMGKIACAN
        tgcggcgcgaatccagtagcagtgtagccagccaccggcaaggcctccggtccaccaagagcgttgaccatggcttccaagtttccgagctgagaagtga
        gacaaagtaaatgtcaacgtaggggatcaatttattagtcaagaccagagtcactcctgtgtacgttctgtggtcttaccattacttaacactgatcgca
        gtcaacgaaggccgccccaaaaattactaatgcattgttcccagtaataccaggacatcatatataataatgtttatattcttcatcccgctgtactccc
                              *
```

```
Query   VLSDLYAIGVTECDNMLMLLGVSSKFTEKERDTVIPLMIHGFKDSAEEAGTSINGGQTVLNPWCLIGGVATTVCQQNEFIMPDNAVPGDVLVLTKPLGTQ
        |||||||/|||||||||||||/||||||/||| |/|/|| ||/| | ||||// |||||/|||||||||||/||||||||||| || |||||||||||||
Target  VLSDLYAMGVTECDNMLMLLGLSSKFTEEERDVVVPMMIQGFRDLAVEAGTNVTGGQTVINPWCLIGGVATSVCQQNEFIMPDQAVVGDVLVLTKPLGTQ
        gcagctgaggagtgaataccgtatatagggcggggcaaacgtagcgggggaagaggcagaacttcaggggatgtccagtaacgcgggggcgcaactgac
        ttgatactgtcagaattttgtgcatcaaagatttctttagtgatctacgcatcggacttacggttggtccctgaaaatttcaacttgattttcactgca
        gtcttctgctgactcgggatagtagtgaaacctagtggcaacacctaatcgtacgtagttctgtgtatgggtataacactgttaaccctatggtataaga

Query   VACNSHQWLEQRNDKWNRIKLVVSEDEVEKAYHDAMFNMARLNRTAAQLMHTFNSHGATDVTGFGILGHAANLAKQQRSEVNFVIHNLPCIAKMAAIAKA
         | |/ ||/ |/|  ||||| |/| //  ||| ||/ /|/|||| ||/||| | /| ||||||||||||| |||||||/|| | ||||| |/||||///|
Target  PAVNAFQWMNQKNQHWNRIKHVISAEDTIKAYSDAILHMSRLNRHAARLMHVFQAHAATDVTGFGILGHAENLAKQQRNEVTFAIHNLPVISKMAAVSRA
        cggagtctaacaacctaaaacgatgggaaagtaggatcatacaccggacacgtcgcggaggagtgatgcggatgacccaggatgacatcgataagggacg
        cctactagtaaaaaagagtaattccaactacagacttatcgtagaccgttattacacccatcgtgttgacaatcaaagaatctctaatcttcatcctggc
        gtccgtaggtagtatgtatgcttggatacagccctcatggaactcggaggcataccgtctaatccagactgtagagatcagtcctctgggagaggcgcat

Query   CGNMFGLLQGTSAETSGGLLICLPREQAAKFCAEIKKVEGNQAWIIGIVEAGNRTARIIEKPRVIEV
            ||||/||||||||||||| | |||| |/| //   ||/||||||/|| |/|/|||| /||/|||
Target  SIVNFGLLKGTSAETSGGLLIVLSREQATKYCQQMIATEGHQAWIIGVVEKGDRSARIIGRPRIIEV
        aagatgtcagatggatggccagctcgcgaattccaagaggccgtaagggggaggatgcaagacaaagg
        gttatgttagcccaccggtttttcgaaccaagaattccagaacgttgttaagagccgttggcgttat
        ccctcaggaatccgatctgacaccagatcgccgagcccaacgggtcgctaaacgtcatccggataac
```

In (Turon 2004), a phylogeny of ascidians is reported. *Halocynthia roretzi* and *Botryllus schlosseri* are in two sister lineages (Styelidae and Pyuridae). *Molgula tectiformis* is not among the specimen analyzed in this paper. This species is classified under the Molgulidae lineage, which together with Styelidae and Pyuridae constitute the order of Stolidobranchiata (NCBI taxonomy). *Ciona* is basal to all others mentioned Ascidia, within the Enterogona order. Finally, *Oikopleura dioica* is a tunicate, but not ascidian, and thus constitutes our outgroup.

Altogether, we think the data clearly strongly support the following gene history (see Figure 5 in main paper).

At the root of tunicates, a single *SPS* gene with selenocysteine was present (*SPS2*) -- as we observe in extant *O.dioica*. Presumably at the root of Ascidians, the same gene originated a secondary isoform with glycine aligned to selenocysteine, as we found today in *Ciona* and in *M.tectiformis (SPS-ae* gene). At the root of Styelidae and Pyuridae, the selenocysteine isoform retrotransposed to the genome, generating a functional, intronless copy of *SPS-Sec*.

The parental gene then lost its *SPS-Sec* isoform, thus specializing only in the *SPS-Gly* isoform. As result, the SECIS downstream of the parental gene (*SPS-Gly*) degenerated, while it was kept in the new *SPS-Sec* gene. This gene duplication is observed in species *H.roretzi* and *B.schlosseri*.

Figures in Supplementary Material S4:

Figure SM4.1: (next page)
Snapshot of the UCSC genome browser of the *Ciona intestinalis* genome at the *SPS* gene locus. The exonic structure of the coding sequence of the two isoforms is shown on top, in blue. The gene is on the negative strand, thus it is to be read from the right to the left. The two forms differ only for the first exons (top right). Just below, the localization of the SECIS element is shown as a black rectangle. Below, the aligned EST sequences available at the genome browser are shown. ESTs support the two isoforms, and show that both share the same 3’ UTR and thus the SECIS, although this is expected useless for the Gly form. For full readability download this from big.crg.cat/SPS and visualize on screen.

Phylogeny of Selenophosphate synthetases (SPS)

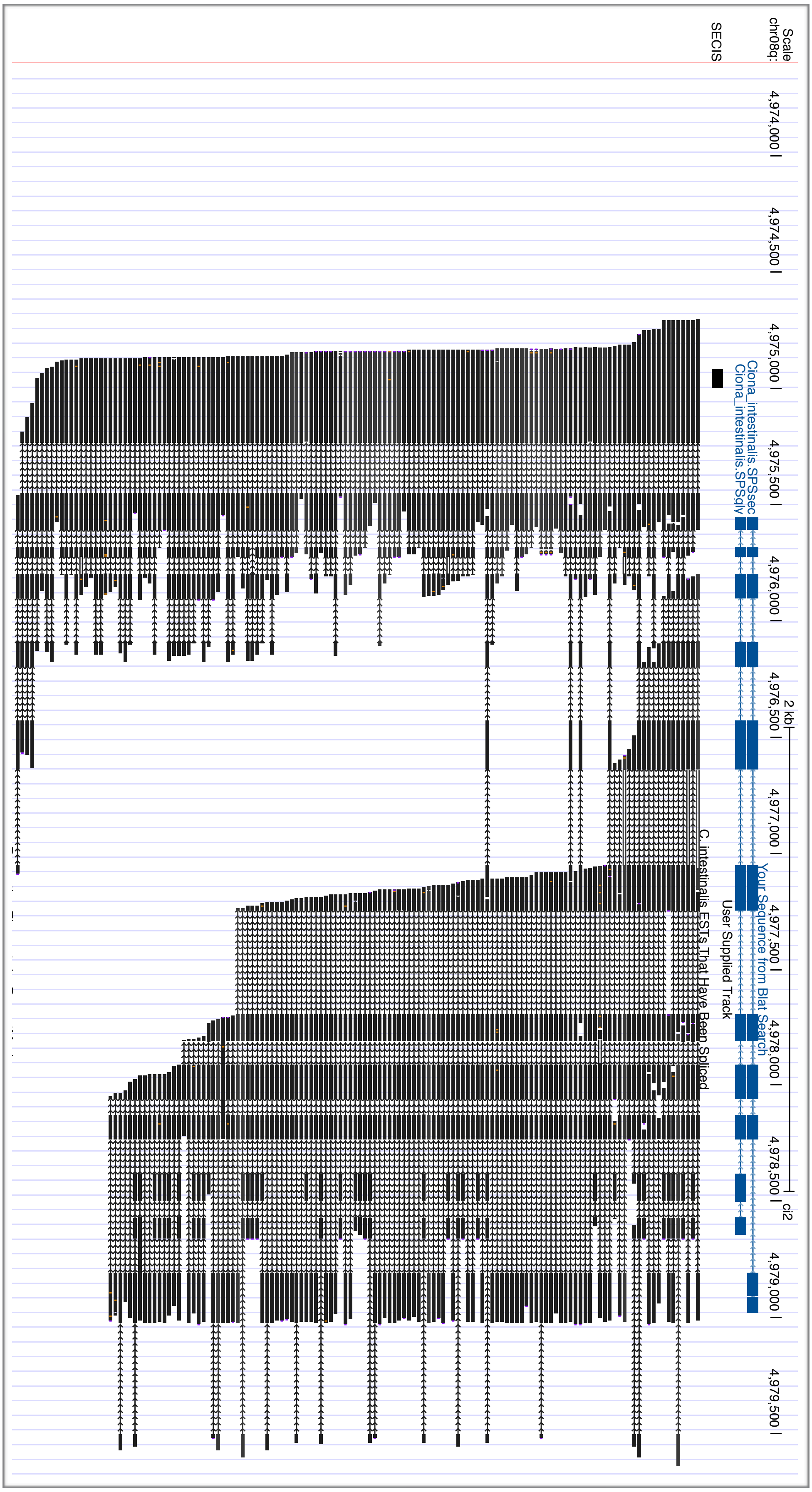

<u>Figure SM4.2:</u>

Alignment of *SPS* genes predicted with selenoprofiles on tunicate ESTs downloaded from NCBI, excluding the *Ciona* genus. The column with selenocysteine is framed in red. On the left, the protein ids assigned by Selenoprofiles allow to identify the target species. The id also contains a label after the amino acid found at the Sec column. In some cases, the label is instead "pseudo", when stop codons or frameshifts are predicted. Given the high level of gene conservation, those are probably caused just by the low quality of some ESTs. For full readability download this from big.crg.cat/SPS and visualize on screen.

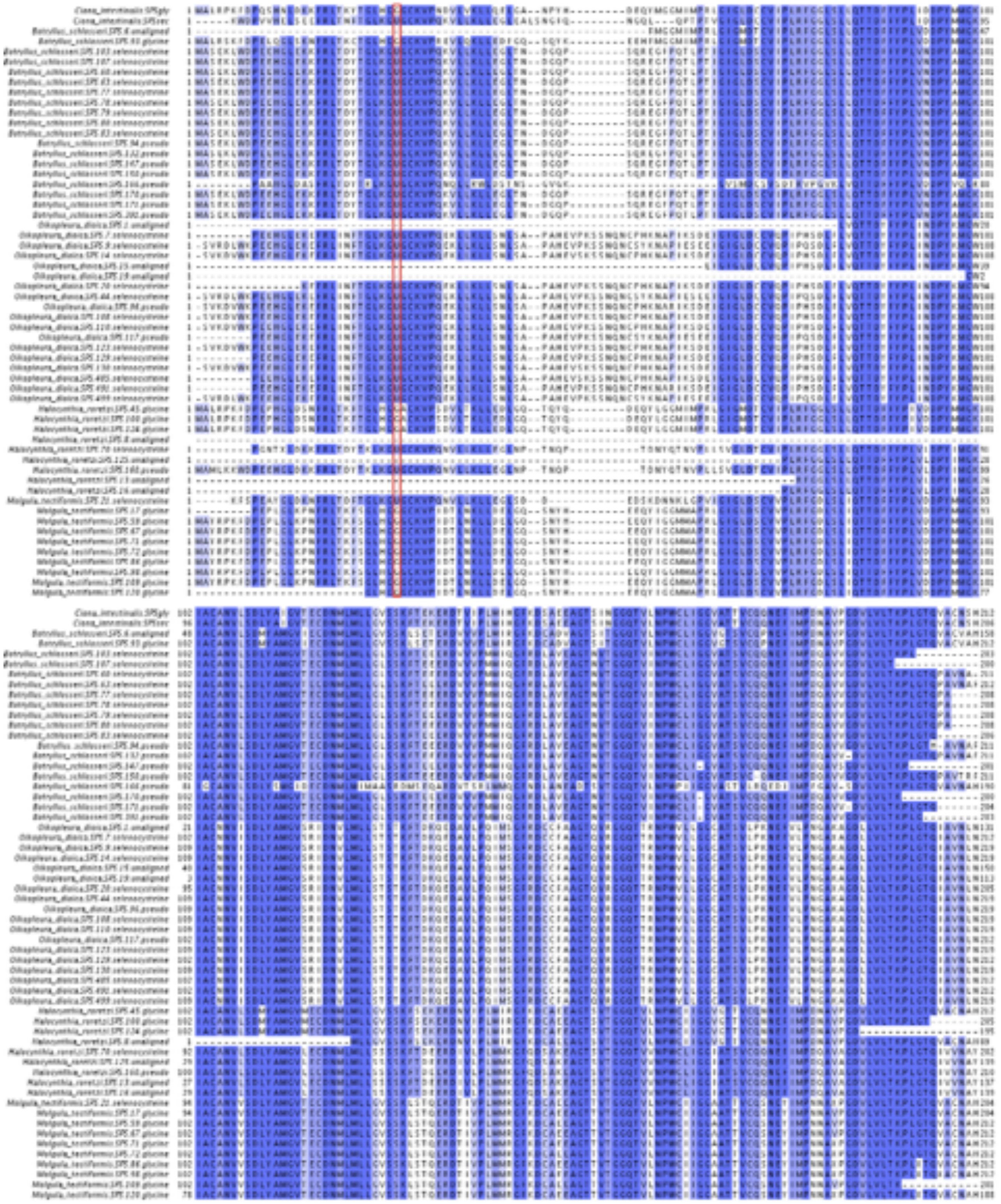

Supplement: Supplemental Material [file supp_gr.190538.115_Supplemental_Material_S4.pdf]
