# Supplementary material for: Evolution of selenophosphate synthetases: emergence and relocation of function through independent duplications and recurrent subfunctionalization
Source: Genome Res. 2015 Sep;25(9):1256–67. doi: 10.1101/gr.190538.115 (PMC4561486; doi:10.1101/gr.190538.115)

Supplementary Material S5:

Evolutionary analysis of metazoan *SPS* genes

The phylogenetic reconstruction of the history of *SPS* duplication was complicated by the high divergence of certain *SPS2* genes, particularly those of insects (see Supplementary Material S3). This caused long-branch attraction, so that in the predicted phylogeny the insect SPS2 genes appear more basal (ancient) than they actually are. This is a very common phylogenetic artifact when different genes in the same tree have different rates of evolution. Thus, we investigated the evolution of metazoan *SPS* genes using sequence-based measures of protein coding constraints.
In particular, we built manually curated alignments of *SPS* coding sequences including *SPS1* and *SPS2* before and after duplication, and we applied methods to measure the rates of non-synonymous and synonymous mutations. These methods were implemented in a package currently under development, called pycodeml, available in a provisional version in Supplementary Material S8, or at https://github.com/marco-mariotti/pycodeml. This program accept as input an alignment of coding sequences and their phylogenetic tree. Hereafter, we briefly describe the statistics that were used and their biological interpretation.

The abundance of available sequences is very heterogenous across metazoans; due to this, we could not investigate all *SPS* duplications with the same depth. For a complete evolutionary analysis, it is necessary to compare several paralogous sequences belonging to each of these classes: *SPS1*, *SPS2* after duplication, *SPS2* in a close outgroup that did not go through *SPS* duplication.
We could obtain solid representative sets for the duplications in insects and in vertebrates, but not for those in Clitellata annelids or in ascidians. However, we built a “summary set” alignment to represents all 4 duplications, with only one representative per class, to analyze general trends. All datasets are available in Supplementary Material S8.
In this document, we include figures compressed for visualization on printed pages. Larger versions of these images, which allow to inspect in detail the protein and nucleotide sequences used for evolutionary analysis, are provided at http://big.crg.cat/SPS.

Sequence statistics

The key statistic that we used is the rate of non-synonymous (non-syn) vs synonymous (syn) mutations (KaKs, also called Ka/Ks, dN/dS, ω). This is computed comparing a reference sequence to a target sequence; conceptually it can be seen as a measure of protein coding constraint during the evolution from the reference to the target sequence. Values close to zero are observed when the number of synonymous mutations fixed is orders of magnitude greater than the number of non-synonymous mutations fixed, which is indicative of very strong constraints acting on the protein sequence. Depending on which sequences are compared, this statistic has slightly different biological meaning, described below.

The first step to compute KaKs is counting the number of syn and non-syn sites in the reference sequence. Each nucleotide is counted as a single site, and all three possible mutations are considered. Depending on the effect of each mutation on the protein sequence, a site can theoretically be completely non-syn (all 3 possible mutations of this site cause an amino acid change; e.g. any of the three positions in any AUG codon), completely syn (none of the 3 possible mutations of this site change the codon translation; e.g. the third position in a ACA codon), or a defined proportion of syn and non-syn (1/3 syn and 2/3 non syn, or *vice versa*). The effective number of syn and non-syn sites in the full sequence is computed as the sum across all sites. Thus, the total number of syn and non-syn sites together is always equal to the nucleotide length of the sequence, but the counts of syn and non-syn sites are not necessarily integer numbers. For any sequence, non-syn sites are typically more numerous than syn sites.
Once the sites are counted, the target sequence is compared position by position with the reference sequence, and each mismatch is categorized either as a syn or non-syn change. The Ks value is then computed as the number of observed synonymous changes divided by the number of syn sites. The Ka value is computed analogously for non-syn sites. The KaKs value is finally computed as the ratio Ka/Ks.
In the program pycodeml, KaKs values can be computed between any pair of sequences in the input alignment, or in other words, between any two nodes in the input tree. Ancestral nodes (non-leaves) can also be considered, since the program codeml (PAML) is used to predict their sequences. For the analysis of *SPS* sequences, we used the following comparisons.

KaKs with root:
This statistic is computed as the KaKs between the ancestral sequence predicted at the root of the tree (reference) and the sequence of any single leaf (target). We used this measure to compare the distribution of rates of evolution of the different classes of *SPS* genes from their predicted common ancestor.

dKaKs:
This statistics can be computed for any node except the root, as the KaKs between its sequence (target) and its immediate parent in the tree (reference), whose sequence is necessarily inferred. It measures the protein coding constraint during the most recent evolution of this sequence.

KaKs lineage:
This statistics can be computed only for an ancestral (non-leaf) node. It is computed as the KaKs between this ancestral node (reference) and all the leaves under this node (targets). The sequences at target nodes are collated so that any specific change at a given site (syn or non-syn alike) is counted only once, even if observed in more than a leaf. When computed for the root of an orthologous group (e.g. ancestor of vertebrate *SPS1* sequences), this statistics measures the protein coding constraint during its evolution, from its origin to its current state in the extant species represented in the input alignment.

KaKs orthologous:
This statistics is computed as the KaKs between any two leaf sequences in the same orthologous group (e.g. vertebrate SPS1 sequences). The distribution of the KaKs between orthologues gives an estimate of the average and the variability in protein constraint within an orthologous group of genes, similarly to KaKs lineage. Unlike KaKs lineage however, this statistics is not affected by ancestral sequence reconstruction.

Evolutionary analysis of the insect *SPS* duplication

In order to perform evolutionary analysis, we have aligned the coding sequences of all insect *SPS* genes in our datasets, which are based on genome sequences. Besides, we also searched all EST and mRNA sequences belonging to non-insect arthropods, which were downloaded from NCBI. This allowed us to obtain additional *SPS2* genes which never duplicated (unduplicated *SPS2*, or just uSPS2), otherwise scarcely represented.
In this process, we came across certain non-insect arthropod species with an apparent *SPS* duplication, similarly to the species *Ixodes scapularis* in our dataset (see figure 2 in main paper). Phylogenetic analysis suggests that these are results of additional, independent duplications of *SPS2* in certain arthropod lineages (not shown). These genes were not included in the current analysis.

The coding sequences of all *SPS* genes considered were aligned based on their translation to amino acids. This alignment was then manually trimmed to remove the N-terminal and C-terminal regions, and also all the columns present only in a minority of sequences (i.e. insertions). This process was carried out to exclude all ambiguous positions from the final alignment, increasing the robustness of the evolutionary analysis.

We obtained the rough phylogenetic tree of all species represented in the set from NCBI taxonomy, and then we refined at every non-dicotomic node using data from literature (mainly The International Aphid Genomics Consortium, 2010). The tree of genes was designed manually, by forcing the topology of the species tree within each orthologous group (i.e. by tree reconciliation). *SPS* gene “classes” were defined and assigned a color for visualization. The classes follow the same classification and colors reported in Figure 4 in the main paper, with one exception: *SPS2* genes are split in two classes, depending on whether they underwent a duplication that generated a *SPS1* gene, or not.

Finally the program pycodeml was run, using as input the alignment of coding sequences of *SPS* genes and their gene tree. The ancestral sequences were predicted using codeml (PAML package); then, synonymous and non-synonymous changes were inferred by comparison. Results are displayed in figure SM5.1. We show 3 different KaKs statistics. In SM5.1 panel A, the KaKs lineage values for some key nodes are shown in blue font, on their respective branches in the tree on the left. Panel B and panel C show the distribution of KaKs with root, and KaKs paralogous statistics, respectively, categorized by the class of SPS gene. For robustness, we excluded the results deriving from comparison of very similar sequences (less than 75 synonymous changes).

Comparing the KaKs of *SPS1* and *SPS2* after duplication, we can see that they are radically different: 0.129 vs 0.668 (KaKs lineage). The distribution of values of the KaKs with root statistics shows the same trend. To make sure that this is not an artifact caused by the ancestral sequence reconstruction procedure, we computed also the distribution of values for the KaKs orthologous statistics, and this show the same trend as well.
After duplication, *SPS2* show a much higher rate of non-synonymous vs synonymous substitution in comparison to *SPS1*. Thanks to the availability of non-insect arthropod unduplicated *SPS2* sequences, we can also estimate the evolutionary rate of *SPS2* before duplication. Regardless of which KaKs method is used, the resulting value on unduplicated *SPS2* matches the values of SPS1 sequences.

The analysis shows that, after duplication, the *SPS2* gene accelerated its rate of protein evolution, consistent with a relaxation in the purifying selection on this gene. In contrast,

the *SPS1* gene is kept under a level of purifying selection similar to the one acting before the duplication. Interestingly, the insect *SPS1* gene shows the same evolutionary patterns in its two forms, as *SPS1-UGA* and as *SPS1-Arg*.

Evolutionary analysis of the vertebrate SPS duplication

We produced a coding sequence alignment and a gene tree representing the SPS duplication in jawed vertebrates, applying a procedure analogous to the one just described for insects. In the case of vertebrates, we could not obtain as many unduplicated *SPS2* sequences as for arthropods. However, we could still get the sequence of *SPS2* in three jawless vertebrate organisms (Cyclostomata). We then performed the same analysis reported above for insects; see figure SM5.2.

Although the KaKs values are lower for vertebrates than for insects, the comparison of the two groups *SPS1* and *SPS2* after duplication follow the same trend. All KaKs statistics tested (figure SM5.2 panels A, B, C) showed a striking difference in the two groups. *SPS2* genes feature higher values than *SPS1* genes, indicating looser protein coding constraints in *SPS2* than in *SPS1*. The estimates of KaKs in unduplicated *SPS2* genes of jawless vertebrates match again those of *SPS1*, lower than duplicated *SPS2*.

Thus, despite the different mechanisms of duplication, the same evolutionary pattern is observed for insect and vertebrate *SPS* genes: KaKs increases in *SPS2* after the duplication to generate *SPS1*. This is consistent with a relaxation in selective constraints, which fits well the subfunctionalization scenario.

Evolutionary analysis in a summary set

Due to the lack of available sequences, for annelids and tunicates we could not perform the same evolutionary analysis as for insects and vertebrates. However, we built a summary set of coding sequences, with a minimal group of genes to represent all duplications we described. For each independent, complete *SPS2* gene duplication (in vertebrates, ascidians, annelids, insects), the summary set contains the two genes resulting from the duplication (*SPS1* and *SPS2*) in a representative species, plus the unduplicated SPS2 of an outgroup species; for example, for the vertebrate duplication, the summary set contains human *SPS1* and *SPS2* sequences, and *SPS2* from sea lamprey (*Petromyzon marinus*).
The results of pycodeml on the summary set is shown in figure SM5.3. We used the dKaKs statistics to characterize the evolution of *SPS1* and *SPS2* genes after duplication. In fact, due to the topology of the gene tree, the dKaKs values in figure SM5.3 correspond to the evolution of *SPS1* and *SPS2* from their last common ancestor before the duplication. For insect and vertebrate *SPS1* and *SPS2*, we can see that these values roughly match those for the KaKs with root statistics in their respective set (figures SM5.1 and SM5.2).

Although in various degrees, the KaKs rate is higher in the *SPS2* gene than in the *SPS1* gene after each of the 4 duplications. Determining how these rates compare to the rate of evolution in the parental *SPS2* gene before duplication is difficult in this set. Nonetheless, we can simply use amino acid sequence identity as a proxy to investigate this.

As an effect of the acceleration in rate of evolution, the sequence identity of *SPS2* genes after duplication is lower than the sequence identity of *SPS1* genes in the same species. This is true for single lineages, thus at the level of phylogenetic paralogues (insects and vertebrates), but it is also true when we consider *SPS2* and *SPS1* genes across different lineages with independent duplications: in the summary set, the average protein sequence identity is 79.9% for *SPS1* genes vs 69.8% for duplicated *SPS2* genes.
Examining the sequence identity of unduplicated *SPS2* genes, we see that again they match the features of *SPS1*, rather than those of *SPS2* genes after duplication. The average protein sequence identity of the unduplicated *SPS2* genes in the summary set matches roughly that of *SPS1*: 78.6%. Also, the unduplicated *SPS2* genes are far more similar to *SPS1* genes, than to *SPS2* genes after duplication.

Summarizing, our analysis shows that there is an evolutionary pattern common to all the metazoan *SPS* duplications that we described: the ratio of non-synonymous/synonymous substitution rates per site increases in the *SPS2* gene after the duplication generating *SPS1*, consistent with a relaxation of protein coding constraints.
Intriguingly, the *SPS1* gene instead exhibits a stricter level of constraint, comparable to that of the parental *SPS2* gene before duplication. We can only speculate the reasons behind this phenomenon. A possible explanation is that, before duplication, the selective constraints acting on the parental (dual-function) *SPS2* gene are majorly ascribed to the *SPS1* function, rather than the canonical *SPS2* function (selenophosphate synthesis). If this is the case, the relocation of the *SPS1* function to a novel gene would indeed release the selective constraints on the parental gene, but not on the novel *SPS1* gene. The conclusion that the *SPS1* function may be more important than the *SPS2* function even for the ancestral *SPS2* gene may seem counterintuitive. However, we believe this is well consistent with the pattern of independent duplications. In fact, if the novel *SPS1* function was overloading the protein coding constraint in the ancestral *SPS2*, it is not so surprising that the gene took any possible chance to relocate this function.

It is striking that the we observed the same evolutionary pattern for the various independent *SPS* duplications, which were very diverse both regarding the molecular mechanisms of the duplication, as well as for the intermediate states (e.g. alternative transcript isoforms in ascidians). We are confident that future research will assess whether this is a general characteristic of the gene duplication/subfunctionalization process, or if this is peculiar to the *SPS* gene history only.

Figures in Supplementary Material S5:

<u>Figure SM5.1:</u> (next page)
Evolutionary analysis of the *SPS* duplication in insects.
Panel A) The tree on the left represents the history of genes as inferred by our phylogenetic reconstruction. The most basal section (on top, with the darkest background), contains the non-insect arthropods unduplicated *SPS2* genes; insect *SPS1* and insect *SPS2* genes are in the middle and bottom section respectively (light grey and medium grey backgrounds, respectively). In the tree, branch lengths are proportional to the ratio of synonymous changes per synonymous site fixed in each node compared to its immediate ancestor (dKs).
Above some selected nodes, the KaKs lineage values (see text) are displayed in blue font. The right section represent the full sequence alignment, trimmed to remove ambiguous positions. Larger version of this plot, including readable amino acid or nucleotide sequences, are available at http://big.crg.cat/SPS. The synonymous and non-synonymous changes are displayed in blue and red, respectively. These were called in comparison to two possible ancestral nodes. Changes with respect to the root of the tree are semi-transparent. Then, for *SPS1* and *SPS2* genes after duplication, the more recent changes with respect to the root of the orthologous group (i.e. ancestral *SPS1* or *SPS2* after duplication) were also drawn on top in opaque colors. Between the tree and the alignment, colored circles are used to display the class of every gene. This plot was generated with pycodeml.
Panel B) Distribution of KaKs values computed comparing every extant sequence with the root (KaKs with root). The fill color is used to represent the class of *SPS* gene (see panel A).
Panel C) Distribution of KaKs values computed comparing extant sequences from the same class (KaKs orthologous). The fill color is used to represent the class of *SPS* gene (see panel A).

Phylogeny of Selenophosphate synthetases (SPS)

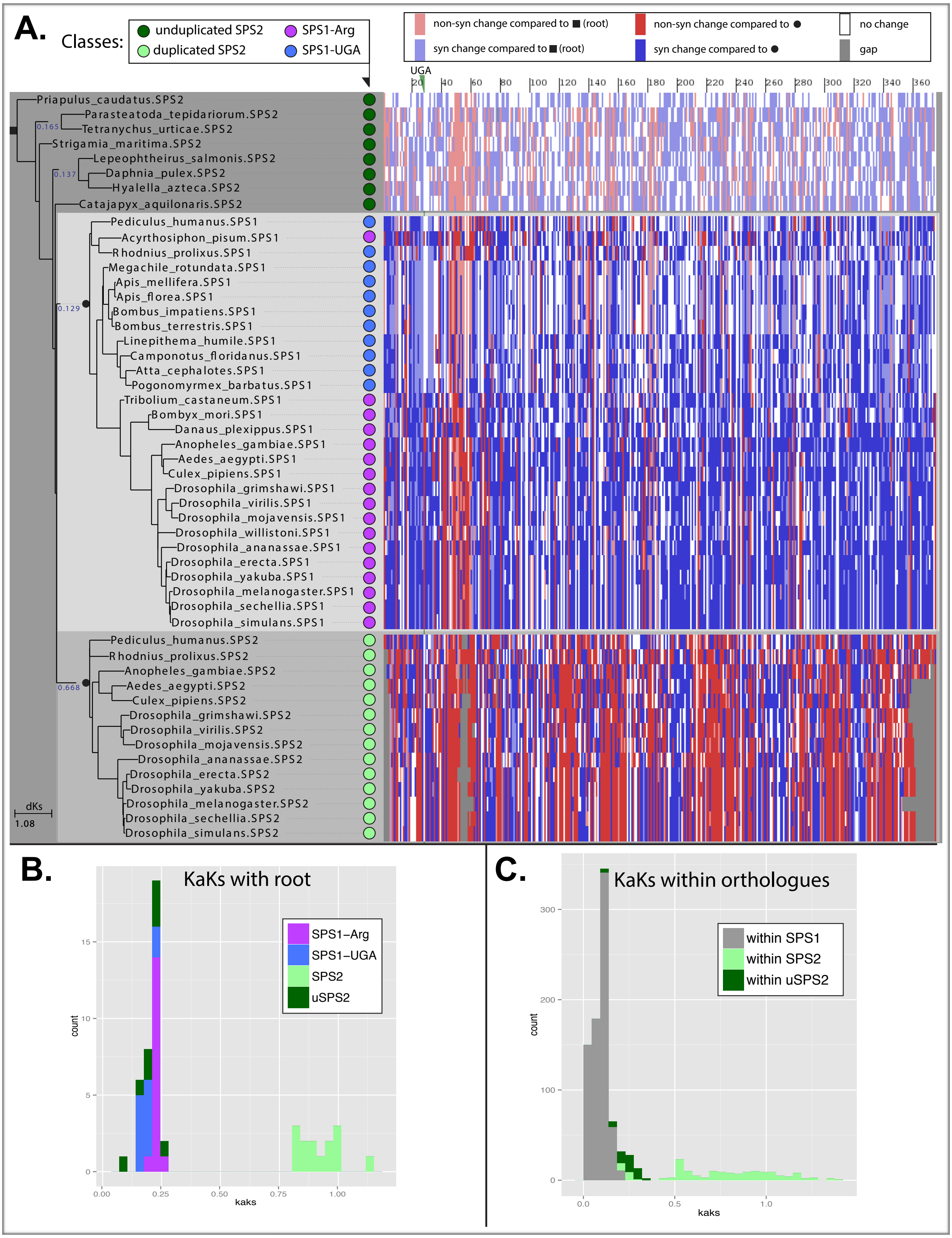

Figure SM5.2: (next page)
Evolutionary analysis of the *SPS* duplication in vertebrates.
Panel A) The tree on the left represents the history of genes as inferred by our phylogenetic reconstruction. The most basal section (on top, with the darkest background), contains the Cyclostomata unduplicated *SPS2* genes; vertebrate *SPS1* and *SPS2* genes are in the middle and bottom section respectively (light grey and medium grey backgrounds, respectively). In the tree, branch lengths are proportional to the proportion of synonymous changes per synonymous site fixed in each node compared to its immediate ancestor (dKs).
Above some selected nodes, the KaKs lineage values (see text) are displayed in blue font. The right section represent the full sequence alignment, trimmed to remove ambiguous positions. Larger version of this plot, including readable amino acid or nucleotide sequences, are available at http://big.crg.cat/SPS. The synonymous and non-synonymous changes are displayed in blue and red, respectively. These were called in comparison to two possible ancestral nodes. Changes with respect to the root of the tree are semi-transparent. Then, for *SPS1* and *SPS2* genes after duplication, the more recent changes with respect to the root of the orthologous group (i.e. ancestral *SPS1* or *SPS2* after duplication) were also drawn on top in opaque colors. Between the tree and the alignment, colored circles are used to display the class of every gene. This plot was generated with pycodeml.
Panel B) Distribution of KaKs values computed comparing every extant sequence with the root (KaKs with root). The fill color is used to represent the class of *SPS* gene (see panel A).
Panel C) Distribution of KaKs values computed comparing extant sequences from the same class (KaKs orthologous). The fill color is used to represent the class of *SPS* gene (see panel A).

Phylogeny of Selenophosphate synthetases (SPS)

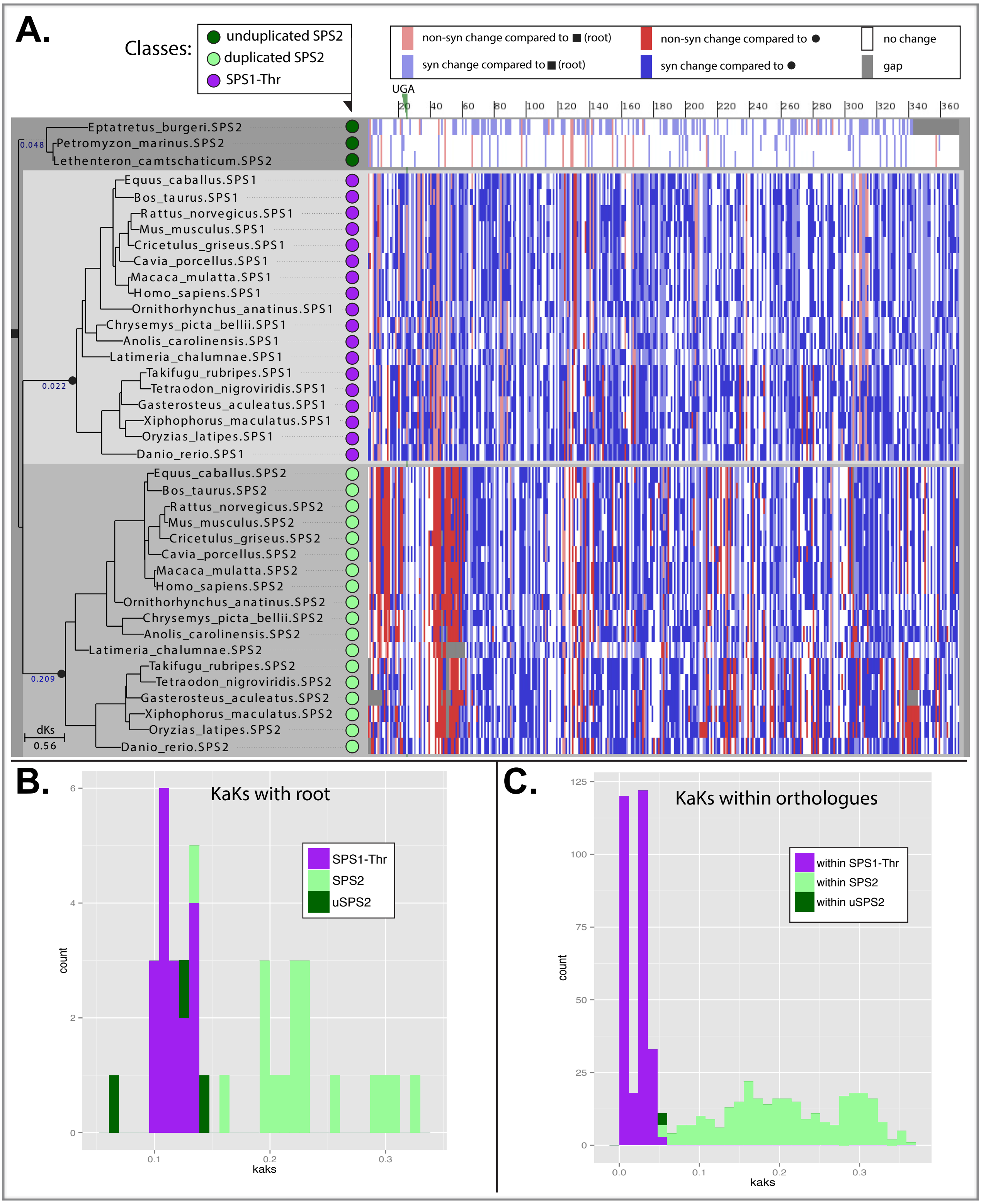

<u>Figure SM5.3:</u>
Evolutionary analysis on a summary set representing all 4 documented *SPS* duplications. One representative per duplication per *SPS* gene class is included; in total there are 4 unduplicated *SPS2* genes, 4 *SPS1* genes and 4 *SPS2* genes after duplication. The duplications represented are those in the following lineages, from top to bottom: jawed vertebrates, Styelidae and Pyuridae ascidians, Clytellata annelids, insects. The tree on the left represent the history of genes as inferred by our phylogenetic reconstruction. On the right, the alignment of coding sequences is represented, with synonymous and non-synonymous changes drawn in blue and red, respectively. The dKaKs values for *SPS1* and *SPS2* after duplication are written on the tree branches in purple font. These represent the KaKs during the evolution of *SPS1* and *SPS2* from the ancestral sequence predicted just before the duplication, to their extant state after duplication. Larger version of this plot, including readable amino acid or nucleotide sequences, are available at http://big.crg.cat/SPS.

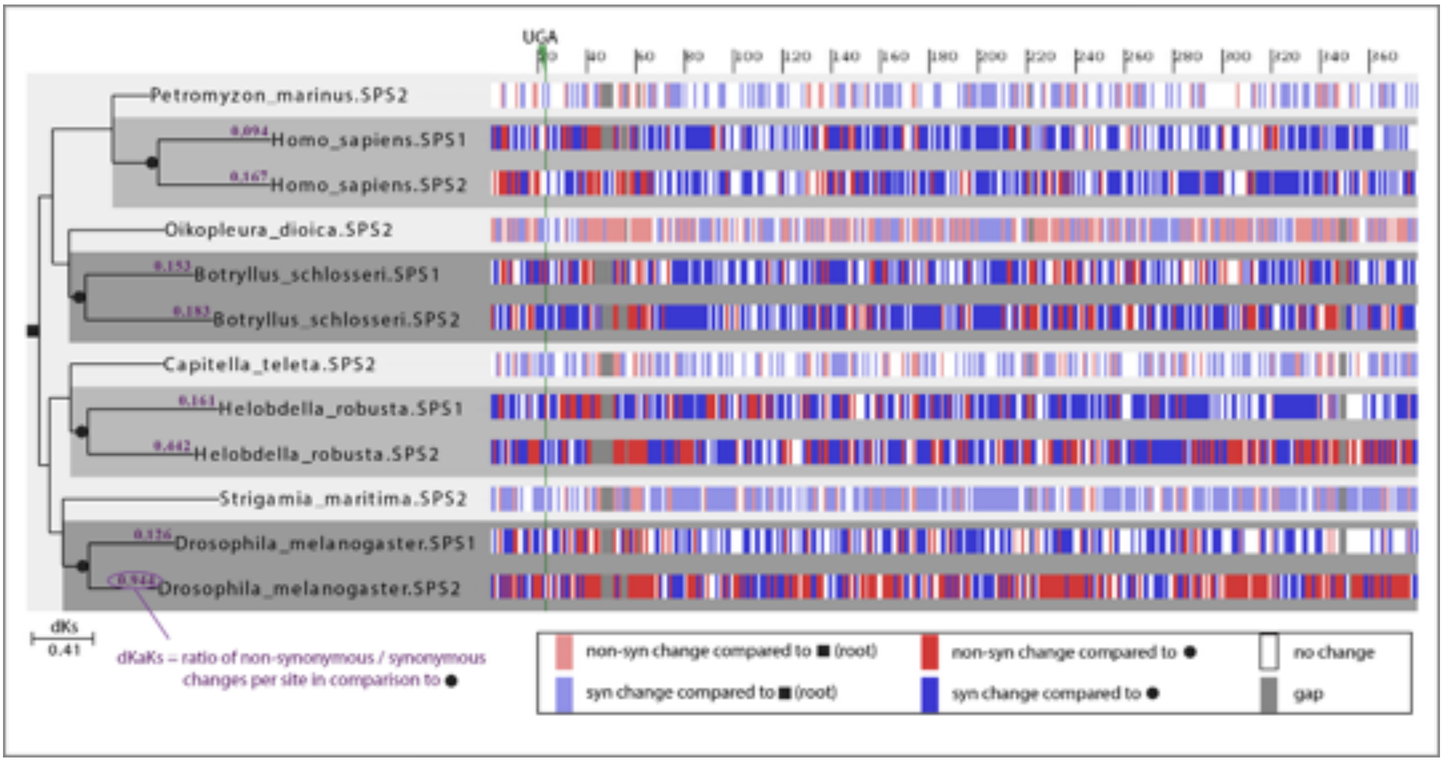

Supplement: Supplemental Material [file supp_gr.190538.115_Supplemental_Material_S5.pdf]
