# Supplementary material for: Evolution of selenophosphate synthetases: emergence and relocation of function through independent duplications and recurrent subfunctionalization
Source: Genome Res. 2015 Sep;25(9):1256–67. doi: 10.1101/gr.190538.115 (PMC4561486; doi:10.1101/gr.190538.115)

Supplementary Material S6:

Secondary structures within coding sequences of *SPS* genes

The program RNAz (Gruber 2010) predicts structured RNA motives in nucleotide alignments of homologous regions in different species. The conservation of base pairings (with particular attention to compensatory mutations), is used to infer the presence of a functional secondary structure in a set of species.
We have run RNAz 2.1 on the coding sequences of all *SPS* genes, to characterize the secondary structures overlapping or just subsequent to the TGA.

Initially, a "master alignment" was produced, including all coding sequences of *SPS* genes in our main dataset. Then, we extracted various subset alignments of *SPS* sequences along the tree of life, based on the residue found at Sec position and on the phylogenetic branching of species. Different resolutions (i.e. lineage depths) were tested; thus, certain subsets are contained in other more general subsets. The final list of lineages for which subsets were created is the following:

- prokaryotes, archaea, bacteria, clostridiales, proteobacteria, campylobacter (epsilonproteobacteria), deltaproteobacteria, pasteurella;
- non-metazoan eukaryotes, metazoa, non-bilaterian metazoa, basal bilateria (non-insecta, non-chordata), vertebrata, insecta, diptera, drosophila, hymenoptera, non-hymenopteran insecta

For each lineage, up to 4 subsets were created, depending on the type of *SPS* found: selenocysteine, cysteine, other residue, all together. The coding sequences of all genes in the filtered set of eukaryotic and prokaryotic *SelD/SPS* predictions were considered.
Then, for each lineage subset, we have trimmed off the alignment columns with more than 70% gaps. Additionally, some sequences were removed from subsets after manual inspection, for carrying large gapped regions.
Full length coding sequences alignments were input to the RNAz utility *rnazWindow.pl*, that partitioned each alignment with a sliding window, 80 or 120 bp wide, with a step size of 20. This program also reduces the number of sequences to six, selecting representatives for each window. For some large sequence subsets, we decided to try also another method to select representatives: we ran trimal (Capella-Gutiérrez 2009) on our full length alignments to select 10, 14, 18, 22, 26 or 30 representatives, and then we fed the resulting alignments to *rnazWindow.pl*. In some cases, this improved the predicted stability and probability score of RNAz hits.
The RNAz output files for all combinations of lineage, *SPS* type, and trimming procedure were parsed and inspected, to produce a reliable set of secondary structures. A few secondary structures were predicted far from the Sec TGA but still within the coding sequence, but only in certain lineages and with narrow combination of parameters. We considered those to be false positives, justified by the huge number of alignments tested. The only region that was consistently predicted to contain secondary structures was where the TGA resides, both in selenocysteine containing genes, and in hymenopteran and paraneopteran *SPS1-UGA*. For each candidate region, we carefully inspected all relevant RNAz outputs to choose the most likely structure, trying to minimize the computed fold

energy. Finally, images were produced as indicated in the RNAz manual, that is to say, using tools from the Vienna RNA package (Lorenz 2011).

Prokaryotes: bSECIS elements

A general structure from the set of all selenocysteine containing SelD proteins in prokaryotes, bacteria or archaea could not be obtained, presumably for that exceeds in diversity the detection power of RNAz. Nonetheless, we could get models for several bacterial sublineages, shown in Figure SM6.1.

In proteobacteria, all three structures (B, C, D) feature two stem-loops (stem1, stem2) downstream of the Sec TGA, separated just by a small, variable bulge. The apex of the second stem is minimal: only 3 or 4 bases are predicted to form this unpaired loop.

An additional stem, which just precedes or includes the Sec TGA, is often predicted (A, C, D). The proteobacteria structures (B, C, D) fit well the consensus model for bSECIS elements proposed in (Zhang 2005). The bSECIS predicted for Clostridiales is somehow different, in that stem2 appears to be located downstream of stem1, in contrast to the rest of predictions in which stem2 falls within the two arms of stem1. However, the features of stem2 alone still fit the bSECIS model.

No structure was predicted in archaeal Sec-*SPS* coding sequences.

Eukaryotes: SRE and HRE, and the readthrough enhancing hexanucleotide

Although rather different in sequence, the eukaryotic consensus structures are similar to the bacterial counterpart, in that they all contain stable stems starting about 2-10 bp downstream of the TGA (see Figure SM6.2). The most stable and large stems were predicted in hymenopteran sequences. As said, hymenoptera lost the ability to produce selenocysteine, and no SECIS is found in the 5'UTR of the only hymenopteran SPS gene. We believe this gene to be readthrough in a Sec independent mechanism, supported by its conservation in all hymenoptera genomes and other criteria (see main text). In respect to a bacterial SECIS, this hymenopteran readthrough element (HRE) contains an additional large upstream stem, forming a 3 stems clover structure with the TGA on the apex of the middle stem. A similar structure is predicted in basal metazoans, although stem lengths are quite different.

In all hymenoptera, we noticed a peculiar readthrough enhancing hexanucleotide (Harrell 2002) extremely conserved, right next to TGA: GGGTG[T/C].

This hexanucleotide can be seen also in the consensus structure for basal metazoa and basal bilateria (figure SM6.2). We thus searched it in all our aligned sequence set. Besides hymenopteran and paraneopteran *SPS1-UGA*, the hexanucleotide was found in a number of some metazoan *SPS2*, phylogenetically located basal to insects, vertebrates or to all bilateria. We noticed an inverse correlation between the presence of the hexanucleotide in a TGA containing *SPS* gene and the presence of a *SPS1* paralogue in the same species (see Figure 6 in the main paper).

Examining results in view of our functional hypotheses

If we inspect this data in the view of our subfunctionalization hypotheses (see paper), we see that it supports it. In fact, we predict that a novel function emerged in the ancestral *SPS2*, before the split of bilateria, and we think that the secondary function was carried out by a non-Sec readthrough isoform. Thus, for those species that possess uniquely the descendant of that *SPS2* gene (no gene duplications, losses, or conversions to Cys), we expect the production of a non-SECIS, non-Sec dependent readthrough isoform to be important. Excluding a few vertebrates and *Ciona*, we observe the presence of the hexanucleotide only in such species. Among vertebrates, the hexanucleotide is visible only in a few species, located basal to the rest. *Ciona* themselves are basal to vertebrates. Thus, the presence of the hexanucleotide in these species suggest that it was present in

their last common ancestor, and it was then lost in most vertebrates, presumably as consequence of the appearance of *SPS1-Thr*: as the secondary function was transferred to another gene, the function of SRE went back to be only a support for Sec insertion — while before it had to maintain also an acceptable level of non-Sec readthrough. Thus, we think that the hexanucleotide was basically free to degenerate after the duplication that generated *SPS1*.

Among ascidians, the hexanucleotide is found only in the most basal *Ciona* lineage, while it is mutated in the rest of species, including *Botryllus* and *Halocynthia*, that possess a retrotransposed copy of the SPS-Gly isoform (see Supplementary Material S4).

Interestingly, in Annelida we see the hexanucleotide in *Capitella sp.1* but not in *Helobdella robusta.* This is consistent with our model, since a duplication presumably transferred the second function to a Leu homologue in the latter but not in the former.

Concluding, we think that the hexanucleotide can be seen as an approximate marker for a conserved non-Sec readthrough, that together with a SECIS element, is an indicator of a double function. Nonetheless, note that we expect the readthrough to be occurring also for a few species without the hexanucleotide (e.g. *Schistosoma*, *Oikopleura*), and *vice versa* in few species we think it is just a relic and it will degenerate in time (e.g. *Ciona*, *Danio*).

Figures in Supplementary Material S6:

<u>Figure SM6.1:</u>
bSECIS elements in prokaryotic *SPS* genes. The structures obtained with sequences of Clostridiales (A), Campylobacter (B), Deltaproteobacteria (C) and Pasteurellales (D) are shown. Red base pairs are conserved in all representatives sequences. Yellow and green base pairs are supported by 2 or 3 different pairs (compensatory mutations). Pale colors indicate only partial sequence support. The Sec TGA is circled in purple. The full size image can be downloaded from http://big.crg.cat/SPS

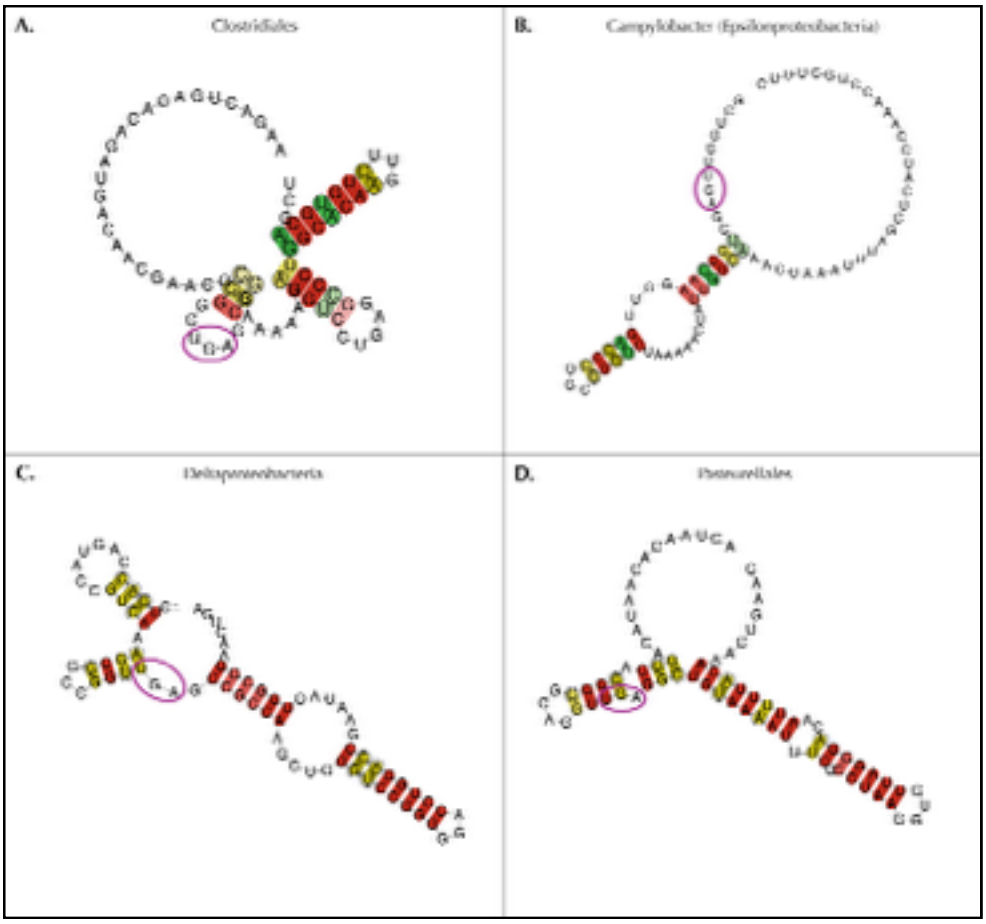

<u>Figure SM6.2:</u>

Recoding elements in eukaryotic *SPS* genes. The structures obtained with sequences of non-bilaterian metazoa (A), non-vertebrate and non-insect bilateria (B), Vertebrata (C), Hymenoptera (D) and Drosophila (E) are shown. See caption of SM6.1 for explanation of nucleotide coloring. The Sec TGA is circled in purple. The presence of readthrough enhancing hexanucleotide GGGTG[C/T] after TGA is indicated with a blue line. The full size image can be downloaded from http://big.crg.cat/SPS

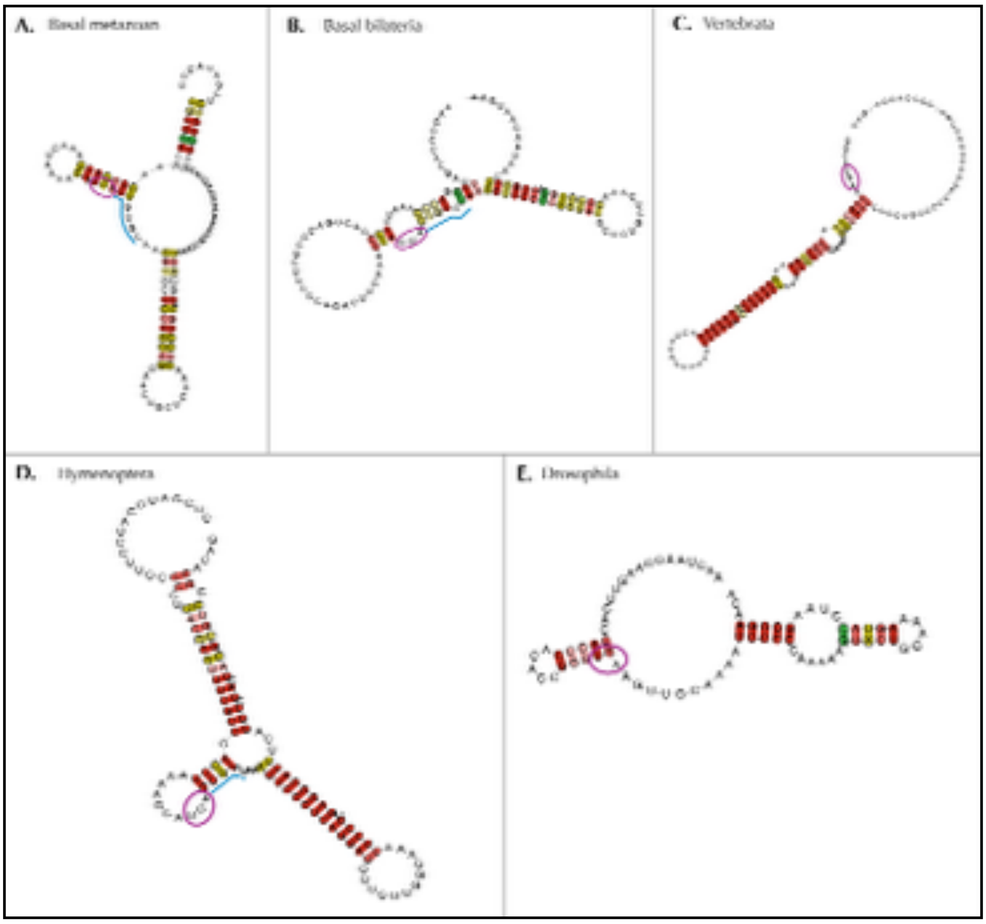

Supplement: Supplemental Material [file supp_gr.190538.115_Supplemental_Material_S6.pdf]
