# Supplementary material for: Evolution of selenophosphate synthetases: emergence and relocation of function through independent duplications and recurrent subfunctionalization
Source: Genome Res. 2015 Sep;25(9):1256–67. doi: 10.1101/gr.190538.115 (PMC4561486; doi:10.1101/gr.190538.115)

Supplementary Material S7:

Rescue experiments in Drosophila

<u>Table SM7.1:</u>
Primers used for cloning the heterologous SPS1 proteins into the *pUAST-attB* vector (see Methods).

Primer name	Primer sequence
Human_SPS1_F	ATTCGTTAACAGATCTATGTCTACGCGGGAGTCCTTTAACC
Human_SPS1_R	TAGAGGTACCCTCGAGACACCCGGGGCCACCTCTTAA
Ciona_SPS1_F	ATTCGTTAACAGATCTATGGCACTAAGACCAAAATTTGACCCCCAATC
Ciona_SPS1_R	TAGAGGTACCCTCGAGCAAATGCAACAGTTTCAGAATCATCACCAGT AA
Atta_SPS1_F	ATTCGTTAACAGATCTATGGCGGAGCTGCAGGGCA
Atta_SPS1_R	TAGAGGTACCCTCGAGTTACTTTTTCATATGAATAAAGTGCTACTTTGT CAATAAAA

Figures in Supplementary Material S7:

<u>Figure SM7.1:</u>
Schema of crosses to obtain a transgenic fly expressing an heterologous *SPS1* in a ptuf mutant background (*ptuf* = drosophila *SPS1* knock out).

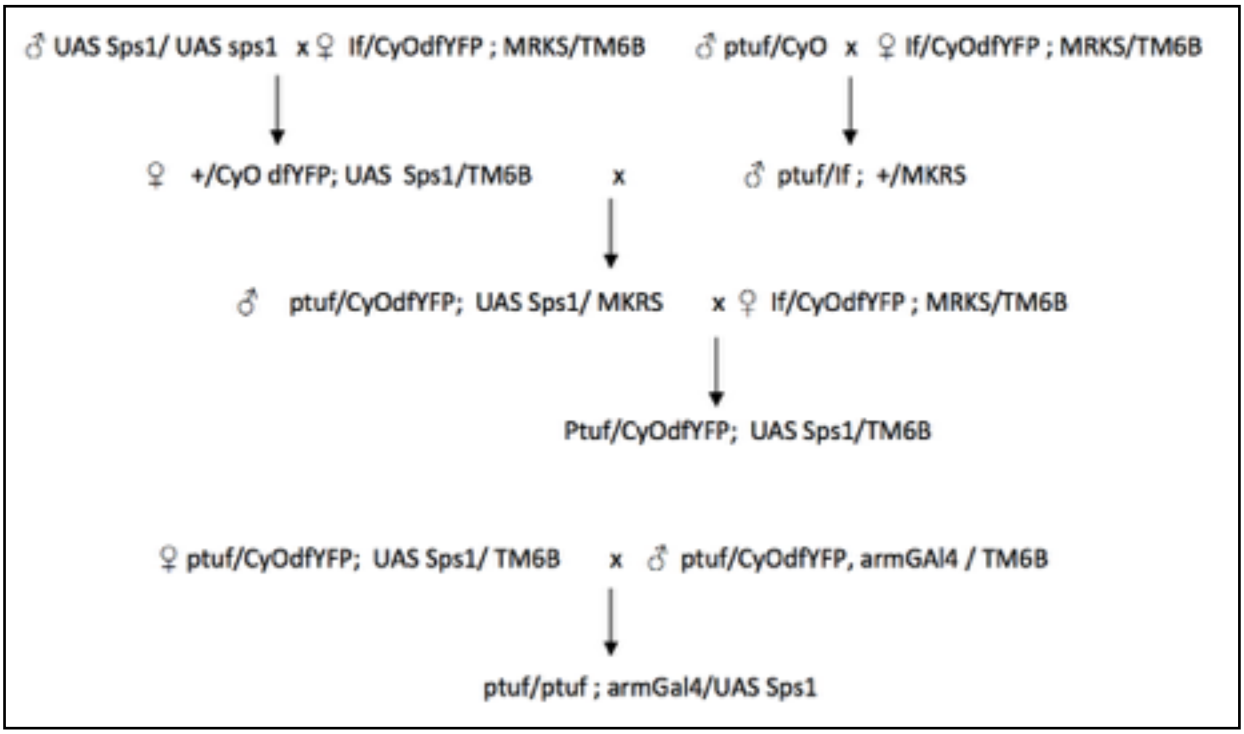

Figure SM7.2:
Expression of exogenous *SPS1* in transgenic drosophila larvae. Real time PCR of RNA extracted from larvae from UAS- lines under the control of the arm-Gal4 driver. All transgenes (Ciona *SPS-Gly*, Atta *SPS1-UGA* and human *SPS1-Thr*) are expressed, although there are differences in their relative expression levels. Control bars represent RNA extracted from larvae containing the UAS- transgenes in the absence of *arm-Gal4* driver.

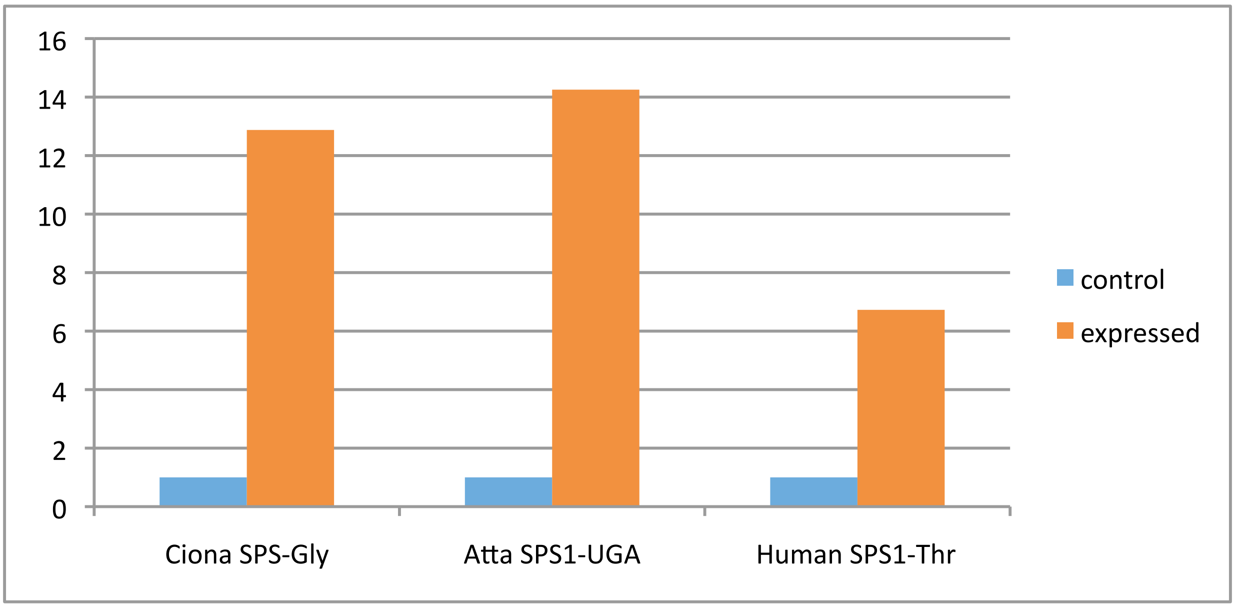

Supplement: Supplemental Material [file supp_gr.190538.115_Supplemental_Material_S7.pdf]
