# Supplementary material for: Evolution of selenophosphate synthetases: emergence and relocation of function through independent duplications and recurrent subfunctionalization
Source: Genome Res. 2015 Sep;25(9):1256–67. doi: 10.1101/gr.190538.115 (PMC4561486; doi:10.1101/gr.190538.115)
Supplement: Supplemental Material [file supp_gr.190538.115_Supplemental_Legends.docx]

Supplementary Material

Find next the following supplementary sections:

• S1: *SelD* in prokaryotes

• S2: Gene fusions and extensions

• S3: Phylogeny of eukaryotic SPS proteins

• S4: Alternative isoforms sorted by gene duplication in ascidians

• S5: Evolutionary analysis of metazoan *SPS* genes

• S6: Secondary structures within coding sequences of *SPS* genes

• S7: Rescue experiments in *Drosophila*

• S8: Datasets
